# Characterization of the small *Arabidopsis thaliana* GTPase and ADP-ribosylation factor-like 2 protein TITAN 5

**DOI:** 10.1242/jcs.262315

**Published:** 2024-08-14

**Authors:** Inga Mohr, Amin Mirzaiebadizi, Sibaji K. Sanyal, Pichaporn Chuenban, Mohammad R. Ahmadian, Rumen Ivanov, Petra Bauer

**Affiliations:** ^1^ Institute of Botany, Heinrich Heine University, 40225 Düsseldorf, Germany; ^2^ Institute of Biochemistry and Molecular Biology II, Medical Faculty and University Hospital Düsseldorf, Heinrich Heine University, 40225 Düsseldorf, Germany; ^3^ Cluster of Excellence on Plant Sciences, Heinrich Heine University, 40225 Düsseldorf, Germany; ^4^ Center for Plant Genome Engineering, Heinrich Heine University, 40225 Düsseldorf, Germany

**Keywords:** TTN5, ARL2, Endomembrane, GTPase, Plasma membrane, Vesicle

## Abstract

Small GTPases switch between GDP- and GTP-bound states during cell signaling. The ADP-ribosylation factor (ARF) family of small GTPases is involved in vesicle trafficking. Although evolutionarily well conserved, little is known about ARF and ARF-like GTPases in plants. We characterized biochemical properties and cellular localization of the essential small ARF-like GTPase TITAN 5 (TTN5; also known as HALLIMASCH, ARL2 and ARLC1) from *Arabidopsis thaliana*, and two TTN5 proteins with point mutants in conserved residues, TTN5^T30N^ and TTN5^Q70L^, that were expected to be unable to perform nucleotide exchange and GTP hydrolysis, respectively. TTN5 exhibited very rapid intrinsic nucleotide exchange and remarkably low GTP hydrolysis activity, functioning as a non-classical small GTPase being likely present in a GTP-loaded active form. We analyzed signals from YFP–TTN5 and HA_3_–TTN5 by *in situ* immunolocalization in *Arabidopsis* seedlings and through use of a transient expression system. Colocalization with endomembrane markers and pharmacological treatments suggests that TTN5 can be present at the plasma membrane and that it dynamically associates with membranes of vesicles, Golgi stacks and multivesicular bodies. Although TTN5^Q70L^ mirrored wild-type TTN5 behavior, the TTN5^T30N^ mutant differed in some aspects. Hence, the unusual rapid nucleotide exchange activity of TTN5 is linked with its membrane dynamics, and TTN5 likely has a role in vesicle transport within the endomembrane system.

## Teaser

**Summary:** The small ARF-like GTPase TTN5 has unusual rapid nucleotide exchange activity. It is likely present in a GTP-loaded form in cells where it is linked with membrane dynamics and vesicle transport within the endomembrane system.

## INTRODUCTION

Regulatory processes in signal transduction rely heavily on guanine nucleotide-binding proteins of the GTPase family. After identifying oncogenes (*HRAS*, *KRAS* and *NRAS*), the RAS superfamily of small GTPases emerged, encompassing conserved members across eukaryotes. This family is divided into five mammalian subfamilies: the Rat sarcoma (RAS), RAS homologs (RHO), RAS-like proteins in the brain (RAB), Ras-related nuclear proteins (RAN) and ADP-ribosylation factor (ARF) subfamilies (Bos, 1988; Kahn et al., 1992; Ahmadi et al., 2017). In *Arabidopsis thaliana*, only four families exist, the Rho of plants (ROP), RAB, RAN and ARF subfamilies (Vernoud et al., 2003). These subfamilies are classified by sequence identity, with conserved sequence motifs playing essential regulatory roles in cells (Kahn et al., 1992). Many mammalian small GTPases act as molecular switches in signal transduction, switching from inactive GDP-loaded to active GTP-loaded forms, enabling differential protein complex formations or acting in tethering complexes to target membranes. With typically low intrinsic GDP-to-GTP exchange and GTP hydrolysis activity, small GTPases require guanine nucleotide exchange factors (GEFs) and GTPase-activating proteins (GAPs) for regulation. GEFs are potentially recruited to inactive GTPases to their site of action and accelerate GDP-GTP exchange leading to GTPase activation. The GTP-loaded GTPases exert their function via direct effector interaction (Sztul et al., 2019; Nielsen, 2020; Adarska et al., 2021) until inactivation occurs through GAP-stimulated GTP hydrolysis. ARF GTPases are often involved in vesicle-mediated endomembrane trafficking in mammalian cells and yeast (Just and Peränen, 2016). In plants, small GTPase activities and their cellular functions are not well understood. Although *Arabidopsis* has 12 ARF and seven ARF-like (ARL) and associated SAR1 proteins, plant ARF proteins are poorly described (Singh et al., 2018). The best-studied plant ARF GTPases, SAR1 and ARF1, act in anterograde and retrograde vesicle transport between the endoplasmic reticulum (ER) and the Golgi. SAR1 is involved in COPII-mediated trafficking from the ER to Golgi, whereas ARF1 participates in the COPI pathway (Singh et al., 2018; Nielsen, 2020). Another ARF-like protein, ARL1, might function in endosome-to-Golgi trafficking (Latijnhouwers et al., 2005; Stefano et al., 2006). These roles of ARF1 and SAR1 in vesicle formation are well conserved in eukaryotes, suggesting that other plant ARF members might also function in the endomembrane system. A recent study has shown Golgi-related localization for some ARF and ARF-like proteins (Niu et al., 2022), promoting a general involvement of ARF proteins in the endomembrane system.

TITAN 5 [TTN5; also known as HALLIMASCH (HAL), ARL2 and ARLC1] is essential for plant development, and was initially identified in two independent screens for abnormal embryo mutants. *ttn5* loss-of-function mutants arrest soon after cell division of the fertilized egg cell, indicating a fundamental, potentially housekeeping, role in cellular activities (Mayer et al., 1999; McElver et al., 2000; Lloyd and Meinke, 2012). The TTN5 sequence is closely related to that of human ARL2 (hsARL2), which has high nucleotide dissociation rates, up to 4000-fold faster than RAS (Hanzal-Bayer et al., 2005; Veltel et al., 2008). HsARL2 is associated with various cellular functions, including microtubule development (Bhamidipati et al., 2000; Fleming et al., 2000; Radcliffe et al., 2000; Antoshechkin and Han, 2002; Tzafrir et al., 2002; Mori and Toda, 2013), adenine nucleotide transport in mitochondria (Sharer et al., 2002) and control of phosphodiesterase activity in cilia (Ismail et al., 2011; Fansa and Wittinghofer, 2016), and yeast and *Caenorhabditis* homologs have been identified (Radcliffe et al., 2000; Antoshechkin and Han, 2002). With regard to TTN5, the cellular roles remain unknown. Its molecular function and the GTPase characteristics of TTN5 have not yet been demonstrated.

Here, we show, by means of stopped-flow fluorimetry kinetic assays, that TTN5 is a functional small GTPase with conserved GTP hydrolysis and rapid nucleotide exchange characteristics. Fluorescence microscopy combined with pharmacological treatments suggests that TTN5 might be located at the plasma membrane (PM) and within the endomembrane system. Our study enables future investigation of cellular and physiological functions of this small GTPase.

## RESULTS

### TTN5 exhibits the atypical characteristics of rapid nucleotide exchange and slow GTP hydrolysis

**Fig. 1.**
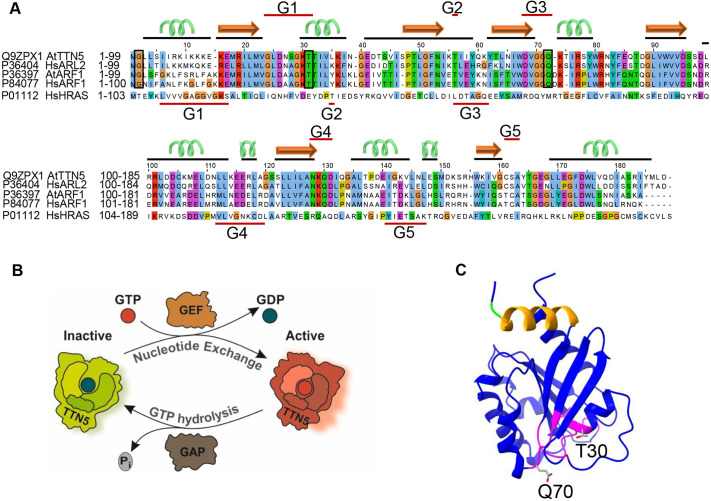
**TTN5 a predicted functional small ARF-like GTPase with nucleotide exchange capacity.** (A) Sequence alignment of TTN5 with HsARL2, HsARF1, HsHRAS and AtARF1 created with Jalview (Waterhouse et al., 2009). Conserved G-motifs (G1–G5; red lines) are defined for TTN5 and HRAS. The TTN5 secondary structure is depicted by black lines and corresponding cartoon (α-helix green; β-sheet orange). Conserved residues in ARF and ARL proteins are highlighted by boxes (G2, and mutated T30 and Q70). TTN5^T30N^ is expected to have a low nucleotide exchange capacity, whereas TTN5^Q70L^ is expected to have a low GTPase hydrolysis activity. (B) Model of the predicted GTPase nucleotide exchange and hydrolysis cycle of TTN5. TTN5 switches from an inactive GDP-loaded to an active GTP-loaded form. GDP to GTP nucleotide exchange and GTP hydrolysis might be aided by GEFs and GAPs. (C) Predicted protein structural model of TTN5; GTP-binding pocket (magenta); N-terminal amphipathic helix (orange); conserved G2 (green); mutagenized T30 and Q70 (sticks). The model was generated with AlphaFold (Jumper et al., 2021), and adapted with UCSF ChimeraX 1.2.5 (Goddard et al., 2018).

TTN5 has higher sequence similarity with HsARL2 than it does with *Arabidopsis* ARF or ARL proteins (Fig. 1A) (McElver et al., 2000; Vernoud et al., 2003). Its ubiquitous gene expression and regulation during plant development, particularly in the root epidermis, as revealed in public RNA-seq datasets of organ and single cell analysis of roots (Fig. S1A,B, and see Materials and Methods), reflect its crucial role. *TTN5* is strongly expressed during early embryo development where cell division, elongation and differentiation take place (Fig. S1C), suggesting that it has function in fundamental processes, especially when cells grow and divide.

**Fig. 2.**
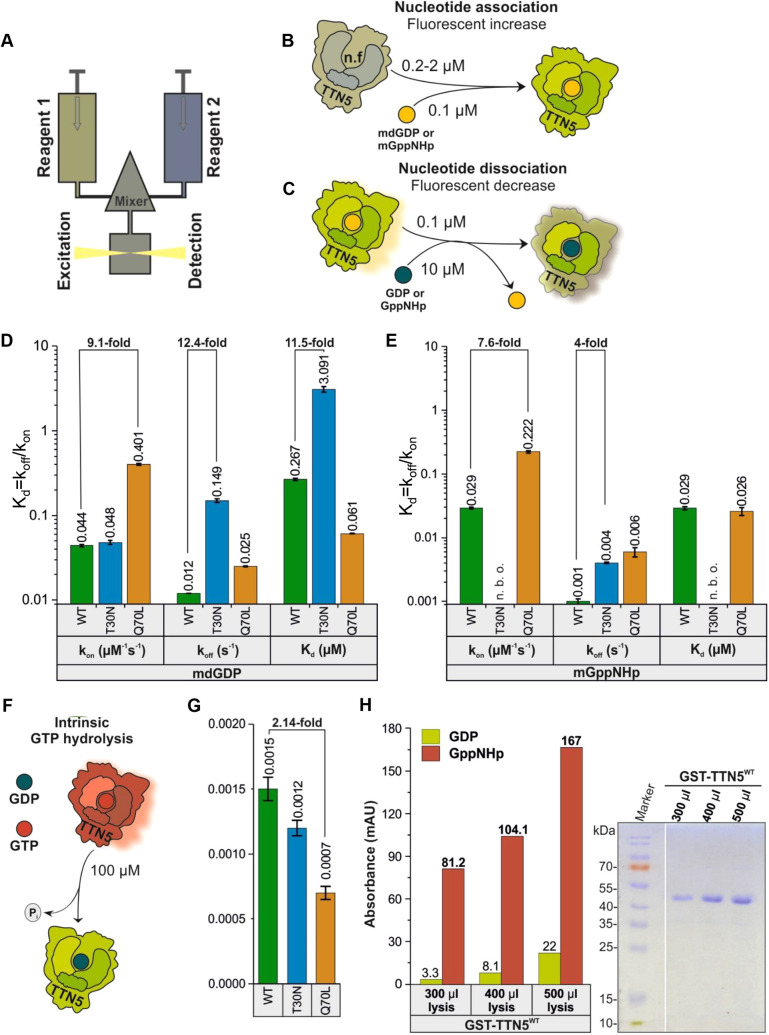
**Biochemical properties of TTN5 suggest its presence in a GTP-loaded active form in cells.** (A) Schematic illustration of the stopped-flow fluorescence device for monitoring nucleotide-binding kinetics of heterologously expressed and purified TTN5 protein (Fig. S2A–D). It consists of two motorized thermostated syringes, a mixing chamber and a fluorescence detector. Two different reagents, 1 and 2, with one containing a fluorescent reporter group (mdGDP or mGppNHp to mimic GDP and GTP) are rapidly mixed and transferred to a fluorescence detection cell. (B) Schematic illustration of nucleotide association. Nucleotide-free TTN5 (for preparation see Fig. S2E) was rapidly mixed with mdGDP. A fluorescence increase is expected upon association of mdGDP with TTN5. Similar measurements were performed with mGppNHp. (C) Schematic illustration of intrinsic nucleotide dissociation. mdGDP-bound TTN5 is mixed with a molar excess of GDP. A fluorescence decrease is expected upon mdGDP dissociation from TTN5 and binding of free unlabeled GDP. Similar measurements were performed with mGppNHp. (D,E) Kinetics of association and dissociation of fluorescent nucleotides mdGDP (D) or mGppNHp (E) with TTN5 proteins (WT, TTN5^T30N^, TTN5^Q70L^). Association rate constants (*k*_on_ in µM^−1^s^−1^) were determined from the plot of increasing observed rate constants (*k*_obs_ in s^−1^) against corresponding TTN5 protein concentrations (as denoted in A,B; for full data, see Figs S3A–F, S4A–E). Intrinsic dissociation rates (*k*_off_ in s^−1^) were determined from the plot of fluorescent decrease upon exchange from mdGDP-bound or mGppNHp-bound TTN5 to GDP-bound TTN5 (as denoted in A,C; for full data, see Figs S3G–I, S4F–H). The nucleotide affinity (dissociation constant *K*_d_ in µM) of the corresponding TTN5 proteins was calculated from the *k*_off_/*k*_on_ ratio. When mixing mGppNHp with nucleotide-free TTN5^T30N^, no binding was observed (n.b.o.) under these experimental conditions. *k*_on_ and *k*_off_ values presented as bar graphs are calculated from the average of four to six measurements and presented as mean±s.d. (F,G) GTP hydrolysis of TTN5 proteins determined by HPLC. (F) Schematic illustration of GTP hydrolysis measurement. (G) GTP-bound TTN5 proteins incubated at different time points before injecting them on a reversed-phase HPLC system. Evaluated data (Fig. S5) resulted in determination of GTP hydrolysis rates (*k*_cat_ in s^−1^). Each bar represents the *k*_cat_ value obtained from a single experiment per condition, comprising six data points. Error bars indicate the standard errors of the fitted values as determined by Origin software. (H) TTN5 accumulation in a GTP-loaded form by HPLC values and Coomassie Blue-stained SDS-PAGE. GST–TTN5^WT^ (46.5 kDa) was purified from bacterial cell lysates at three different volumes in the presence of free GppNHp. Presence of much higher amounts of GppNHp-bound versus GDP-bound GST-TTN5 protein indicates that TTN5 rapidly exchanged bound nucleotide and accumulated in this state. This was a confirmatory experiment performed once.

Based on sequence similarity and structural predictions, TTN5 is presumed to function as a molecular switch. (Fig. 1B,C). Although HsARL2 shows fast GDP-GTP exchange characteristics (Hanzal-Bayer et al., 2005; Veltel et al., 2008), it was unclear whether plant TTN5 shared these characteristics. We here characterized the nucleotide binding and GTP hydrolysis properties of TTN5^WT^ and two mutants, TTN5^T30N^ and TTN5^Q70L^, using heterologously expressed proteins and *in vitro* biochemical assays (experimental workflow illustrated in Fig. S2A–E), as previously established for human GTPases (Eberth and Ahmadian, 2009). TTN5^T30N^ was chosen because it is assumed to preferentially bind GEFs, sequestering them from their proper context, whereas TTN5^Q70L^ was chosen because it is thought to be defective in hydrolyzing GTP; equivalent mutants have been frequently used and characterized dominant-negative and constitutively active (Scheffzek et al., 1997; Zhou et al., 2006; Newman et al., 2014). We monitored the real-time kinetics of interactions of fluorescent guanine nucleotides using stopped-flow fluorimetry suited for very rapid enzymatic reactions (Fig. 2A–C). 2-deoxy-3-O-N-methylanthraniloyl-deoxy-GDP (mdGDP) and GppNHp (mGppNHp), a non-hydrolyzable GTP analog, were used to mimic GDP and GTP binding to TTN5. This approach allowed us to monitor real-time kinetics and determine and quantify nucleotide association (*k*_on_) and dissociation (*k*_off_) characteristics of small GTPases, such as has been done for HsARL2 and HsARL3 (Hillig et al., 2000; Hanzal-Bayer et al., 2005; Veltel et al., 2008; Zhang et al., 2018). The *k*_on_ value is defined as the rate of nucleotide binding to GTPases, to form the GTPase–nucleotide complex (Fig. 2B), whereas the *k*_off_ value describes the rate of nucleotide dissociation from GTPases (Fig. 2C). TTN5 proteins were able to bind both nucleotides, except for mGppNHp binding by TTN5^T30N^ (Figs S3A–F, S4A–E). TTN5^Q70L^ revealed the highest *k*_on_ value for mGDP binding (0.401 µM^−1^s^−1^), being 9-fold higher compared to that of TTN5^WT^ (0.044 µM^−1^s^−1^) and TTN5^T30N^ (0.048 µM^−1^s^−1^), respectively (Fig. 2D, Fig. S3D–F). *k*_on_ values for mGppNHp binding were about half for TTN5^WT^ (0.029 µM^−1^s^−1^) and TTN5^Q70L^ (0.222 µM^−1^s^−1^) compared to those for mGDP binding (Fig. 2E; Fig. S4C,D). The differences in *k*_on_ for the respective nucleotide binding were small. However, TTN5^Q70L^ showed a 7.5-fold faster mGppNHp binding than TTN5^WT^. Remarkably, we were not able to monitor mGppNHp association with TTN5^T30N^ but observed its dissociation (*k*_off_=0.026 s^−1^; Fig. 2E). To confirm the binding capability of TTN5^T30N^ with mGppNHp, we measured the mGppNHp fluorescence in real-time before and after titration of nucleotide-free TTN5^T30N^ and binding occurred too fast to resolve the association rate (Fig. S4B).

We next measured the dissociation (*k*_off_) of mdGDP and mGppNHp from TTN5 proteins with excess amounts of GDP and GppNHp, respectively (Fig. 2C) with interesting differences (Fig. 2D,E; Figs S3G–I,S4F–H). First, TTN5^WT^ showed a 100-fold faster *k*_off_ value (mGDP, 0.012 s^−1^) (Fig. 2D; Fig. S3G), compared to classical small GTPases, including RAC1 (Haeusler et al., 2006) and HRAS (Gremer et al., 2011), but very similar to the *k*_off_ value of HsARF3 (Fasano et al., 2022). Second, *k*_off_ values for mGDP and mGppNHp were in a similar range for TTN5^WT^ (mGDP, 0.012 s^−1^; mGppNHp, 0.001 s^−1^) and TTN5^Q70L^ (mGDP, 0.025 s^−1^; mGppNHp, 0.006 s^−1^), but *k*_off_ values differed 10-fold between the two nucleotides in TTN5^WT^ (Fig. 2D,E; Figs S3G,I, S4F,H). Thus, mGDP dissociated from proteins 10-fold faster than mGppNHp. Third, mGDP dissociation from TTN5^T30N^ (0.149 s^−1^) was 12.5-fold faster than that of TTN5^WT^ and 37-fold faster than mGppNHp dissociation of TTN5^T30N^ (0.004 s^−1^; Fig. 2D,E; Figs S3H, S4G). Mutants of CDC42, RAC1, RHOA, ARF6, RAD, GEM and RAS GTPases that are equivalent to TTN5^T30N^ display decreased nucleotide binding affinity and therefore tend to remain in a nucleotide-free state, complexed with their cognate GEFs (Erickson et al., 1997; Ghosh et al., 1999; Radhakrishna et al., 1999; Jung and Rösner, 2002; Kuemmerle and Zhou, 2002; Wittmann et al., 2003; Nassar et al., 2010; Huang et al., 2013; Chang and Colecraft, 2015; Fisher et al., 2020; Shirazi et al., 2020). Given that TTN5^T30N^ exhibited fast nucleotide dissociation, these results suggest that TTN5^T30N^ might act in either a dominant-negative or fast-cycling manner as reported for other GTPase mutants (Fiegen et al., 2004; Wang et al., 2005; Fidyk et al., 2006; Klein et al., 2006; Soh and Low, 2008; Sugawara et al., 2019; Aspenström, 2020).

The dissociation constant (*K*_d_) is calculated from the ratio *k*_off_/*k*_on_, which inversely indicates the affinity of interactions between proteins and nucleotides (higher *K*_d_=lower affinity). Interestingly, TTN5^WT^ binds mGppNHp (0.029 µM) 10-fold tighter than mGDP (0.267 µM), a difference, which was not observed for TTN5^Q70L^ (mGppNHp, 0.026 µM; mGDP, 0.061 µM; Fig. 2D,E). The lower affinity of TTN5^WT^ for mdGDP compared to mGppNHp brings us closer to the hypothesis that classifies TTN5 as a non-classical GTPase prone to remain in the active, GTP-bound state (Jaiswal et al., 2013). The *K*_d_ value for the TTN5^T30N^–mGDP interaction was 11.5-fold higher (3.091 µM) than for TTN5^WT^, suggesting that this mutant exhibited faster nucleotide exchange and lower affinity for nucleotides and might behave in a dominant-negative manner in signal transduction, similar to what occurs with other GTPases with a T30N exchange (Vanoni et al., 1999).

To get hints on TTN5 functionalities during the GTPase cycle, it is crucial to determine its ability to hydrolyze GTP. Accordingly, the catalytic rate of intrinsic GTP hydrolysis (*k*_cat_), was determined by incubating GTP-bound TTN5 proteins and analyzing the samples at various time points (Fig. 2F; Fig. S5). Determined *k*_cat_ values were quite remarkable in two respects (Fig. 2G). First, all TTN5 proteins, TTN5^WT^, TTN5^T30N^ and TTN5^Q70L^, showed quite similar *k*_cat_ values (0.0015 s^−1^, 0.0012 s^−1^, 0.0007 s^−1^; Fig. 2G; Fig. S5). TTN5^Q70L^ GTP hydrolysis activity was unexpectedly high given that such glutamine mutations typically impair hydrolysis and result in constitutively active GTPases (Hodge et al., 2020; Matsumoto et al., 2021)*.* Second, the *k*_cat_ value of TTN5^WT^ (0.0015 s^−1^) although comparatively low to other GTPases (Jian et al., 2012; Esposito et al., 2019), was 8-fold lower than the determined *k*_off_ value for mGDP dissociation (0.012 s^−1^; Fig. 2E). This means that a fast intrinsic GDP/GTP exchange versus a slow GTP hydrolysis can have drastic effects on TTN5 activity in resting cells, given that TTN5 can accumulate in its GTP-bound form, unlike classical GTPases (Jaiswal et al., 2013). To investigate this scenario, we pulled down GST–TTN5 protein in the presence of an excess amount of GppNHp and measured the nucleotide-bound form of GST–TTN5. Isolated GST–TTN5 bound to increasing amounts of GppNHp, indicating that the bound nucleotide is rapidly exchanged for free nucleotide (here GppNHp; Fig. 2H), which is in contrast to what is seen for classical GTPases, which remain in their inactive GDP-bound forms under the same experimental conditions (Walsh et al., 2019; Hodge et al., 2020).

In summary, TTN5 contains conserved regions required for nucleotide binding and binds nucleotides. Interestingly, the slow intrinsic GTP hydrolysis rates in combination with high GDP dissociation rates indicates that TTN5 tends to exist in a GTP-loaded form, as opposed to the classical GTPases. This might have drastic effects on TTN5 activity in cells under resting conditions (Jaiswal et al., 2013). By contrast, the TTN5^Q70L^ mutant, which we originally suspected would be constitutively active, still has intrinsic GTPase activity, whereas the T30N variant exhibits a low affinity for mGDP. Therefore, we propose that TTN5 exhibits typical functions of a small GTPase based on *in vitro* biochemical activity studies, including guanine nucleotide association and dissociation, and emphasize its divergence among the ARF GTPases because of its kinetics.

### TTN5 may be a highly dynamic protein and localize to different intracellular compartments

**Fig. 3.**
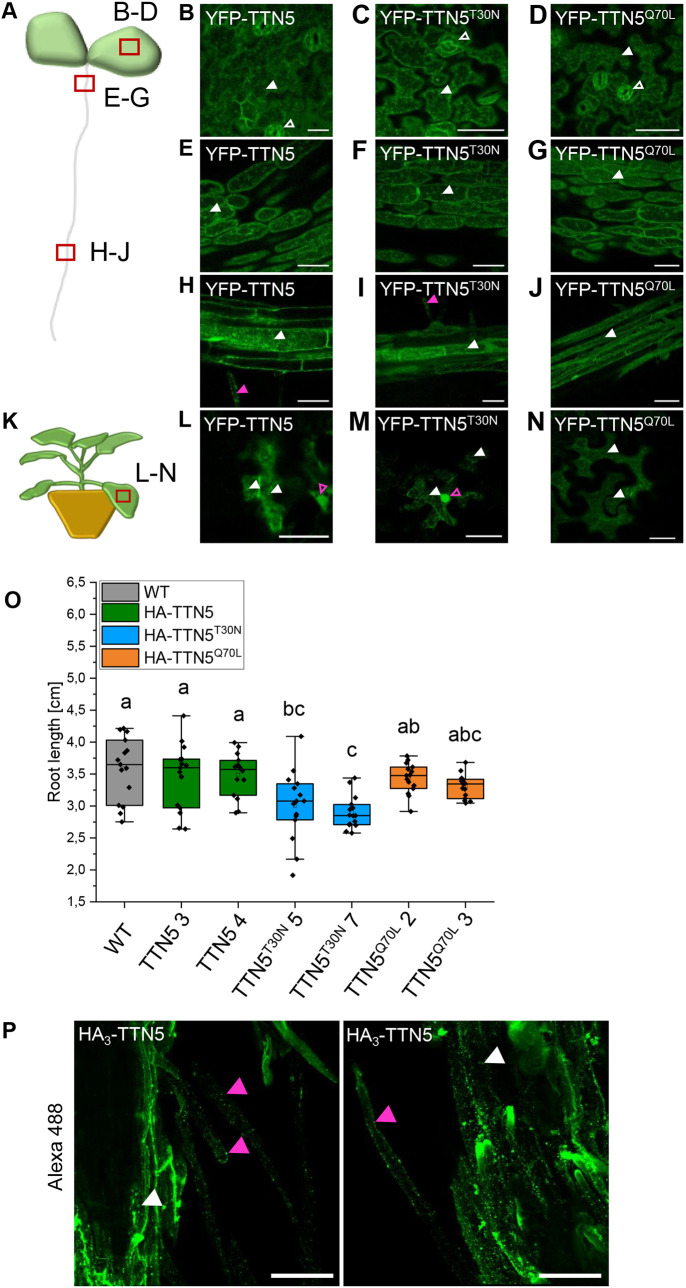
**TTN5 might be present in punctate structures in seedlings.** Microscopy observations of YFP fluorescence were made in a plane underneath the PM at the cell peripheries. (A) Schematic representation of an *Arabidopsis* seedling with indicated imaged positions (red rectangle). (B–J) Analysis of YFP–TTN5, YFP–TTN5^T30N^ and YFP-TTN5^Q70L^ Arabidopsis seedlings via fluorescent confocal microscopy. (B–D) Fluorescence signals observed in stomata (empty white arrowhead) and in the epidermis of cotyledons in punctate structures (filled white arrowhead). (E–G) Localization in hypocotyls showed a similar pattern of punctate structures. (H–J) Signals were present in punctate structures in the root hair zone and root hairs (filled magenta arrowhead). (K) Schematic representation of a *N. benthamiana* plant, used for transient expression with indicated imaged position (red rectangle). (L–N) YFP signals in *N. benthamiana* leaf epidermal cells expressing YFP–TTN5, YFP–TTN5^T30N^ and YFP–TTN5^Q70L^. Signals were present in punctate structures (white arrowheads) and in nuclei (empty magenta arrowheads). Experiments were repeated twice with two seedlings (*n*=2) or one plant (*n*=1). (O) Root length measurement of 10-day-old HA_3_–TTN5, HA_3_–TTN5^T30N^ and HA_3_–TTN5^Q70L^ *Arabidopsis* seedlings in comparison with wild-type (WT). Only HA_3_–TTN5^T30N^ showed slightly reduced root length compared to WT. Analysis was conducted in replicates (*n*=14). The box represents the 25–75th percentiles, and the median is indicated. The whiskers show the 5–95th percentiles. One-way ANOVA with Tukey post-hoc test was performed. Different letters indicate groups that have a statistical significance difference at *P*<0.05. (P) Maximum intensity projection of whole-mount immunostaining of HA_3_–TTN5 roots in the differentiation zone (anti-HA primary antibody, Alexa-488-labeled secondary antibody). Alexa Fluor 488 signals were present in punctate structures in root cells (filled white arrowhead) and root hairs (filled magenta arrowhead), which is comparable to what was seen for YFP signals (H–J). Experiment was repeated three times with two seedlings (*n*=2). Scale bars: 50 µm.

Several eukaryotic ARF GTPases function in vesicle transport and are located at various membranous sites linked with the endomembrane compartments (Vernoud et al., 2003). Localization had not been comprehensively studied for TTN5. To obtain hints as to where in a cell TTN5 localizes to, we first created transgenic *Arabidopsis* lines constitutively expressing YFP-tagged TTN5 (pro35S::YFP–TTN5) and its two mutant forms (pro35S::YFP–TTN5^T30N^, pro35S::YFP–TTN5^Q70L^) and investigated the localization in 6-day-old seedlings in the epidermis of cotyledons, hypocotyls, root hair zone and in root tips (Fig. 3A; Fig. S6A). Microscopy observations were made in different planes of the tissues, for example, inside the cells across the vacuoles (Fig. S6) and underneath the PM at the cell peripheries (Fig. 3). We chose to investigate YFP–TTN5 in the epidermis, as *TTN5* transcripts were detected there in plants (Fig. S1B). YFP signals in epidermal cotyledon cells of YFP–TTN5 seedlings were detected in nuclei and cytoplasm and/or in close proximity to the PM (Fig. S6B). Similar localization patterns were found for mutant YFP–TTN5 signals (Fig. S6C,D). YFP signals in YFP–TTN5, YFP–TTN5^T30N^ and YFP–TTN5^Q70L^ seedlings were also present in a similar pattern in stomata (Fig. 3B–D). In hypocotyls, an intracellular YFP signal was observed in nuclei and in close proximity to or at the PM with all three YFP–TTN5 forms (Fig. S6E–G). Investigation of the root hair zone showed YFP signals in the cytoplasm and at the PM of root hairs (Fig. S6H–J). In the root tip, YFP signal was detectable inside the cytoplasm and in nuclei (Fig. S6K). The pattern was similar for YFP–TTN5^T30N^ and YFP–TTN5^Q70L^ (Fig. S6L,M). Fluorescence signal in YFP–TTN5, YFP–TTN5^T30N^ and YFP–TTN5^Q70L^ seedlings inside the cytoplasm was confined to punctate structures, indicating that fluorescence was present in vesicle-like structures together with free signal. This localization pattern was also present in leaf epidermal cells of the cotyledons (Fig. 3B–D), in the hypocotyls (Fig. 3E–G) and in cells of the root hair zones and in root hairs (Fig. 3H–J). These observed structures point to an association of TTN5 with vesicle and endomembrane trafficking. A closer inspection of the dynamics of these structures in the leaf epidermis of cotyledons showed high mobility of fluorescent signals (Movies 1–3) as well as in hypocotyl cells (Movie 4). Interestingly, the mobility of these punctate structures differed within the YFP–TTN5^T30N^ hypocotyl cells, but not in the leaf epidermis cells (Movie 5, compare with Fig. S2), which was not the case for the YFP–TTN5^Q70L^ mutant (Movie 6, compare with Fig. S5). We detected that movement of YFP–TTN5^T30N^ was slow or completely arrested for approximately half of the cells within the hypocotyl epidermis compared to movements for YFP–TTN5 and YFP–TTN5^Q70L^ (Movies 4–6). This loss of fluorescence signal mobility in YFP–TTN5^T30N^ seedlings might be a consequence of a missing effector interaction. We did not observe the blocked mobility for fluorescence signals in cells expressing YFP–TTN5, YFP–TTN5^T30N^ or YFP–TTN5^Q70L^ in the root elongation zone (Movies 7–9). No mobility of YFP fluorescence signal was visible in root tip cells for any YFP–TTN5 form (Movies 10–12).

To evaluate the *Arabidopsis* data and to better visualize YFP–TTN5, we expressed YFP–TTN5 constructs transiently in *Nicotiana benthamiana* leaf epidermis cells. Fluorescence signals in YFP–TTN5-, YFP–TTN5^T30N^- and YFP-TTN5^Q70L^-expressing cells were all localized at or in close proximity to the PM and in several cytosolic punctate structures, apart from nuclei, similar to what was seen in *Arabidopsis* cotyledons, hypocotyls and root hair zones (Fig. 3K–N; Fig. S6N–Q). Additionally, YFP signals were detected in a net-like pattern typical for ER localization (Fig. 3M,N). This indicates that the fluorescent signal localization is similar in both *Arabidopsis* epidermis cells and *N. benthamiana* leaf epidermis.

It should be noted that the 35S promoter-driven YFP–TTN5 constructs did not complement the *ttn5-1* embryo-lethal phenotype (Fig. S7A,B). Western blot analysis with anti-GFP antibody using YFP–TTN5 *Arabidopsis* seedlings revealed three weak YFP bands ranging between 26 and 35 kDa, besides the expected and strong 48 kDa YFP–TTN5 band (Fig. S7C). We cannot explain the presence of these small protein bands. They might correspond to free YFP, proteolytic products or potentially to proteins produced from aberrant transcripts with perhaps alternative translation start or stop sites. On the other hand, a 35S promoter-driven triple hemagglutinin-tagged HA_3_–TTN5 did complement the *ttn5-1* embryo-lethal phenotype (Fig. S7D,E). Western blot analysis with anti-HA antibody performed with HA_3_–TTN5 seedlings showed a single, correctly sized band, but no band that was 13 to 18 kDa smaller (Fig. S7D). Hence, the inability of YFP–TTN5 to complement the embryo-lethal phenotype is presumably due to the relatively large YFP tag in comparison with the small GTPase and smaller HA_3_ tag. Interestingly, HA_3_–TTN5^T30N^ seedlings presented a shorter root length phenotype, which might be due to the atypical biochemical TTN5^T30N^ characteristics, whereas HA_3_–TTN5 and HA_3_–TTN5^Q70L^ seedlings had no obvious phenotypic difference from wild-type (Fig. 3O).

To verify that the localization patterns observed with YFP–TTN5 constructs are representative of a functional TTN5, we performed immunofluorescence staining against the HA_3_ tag in HA_3_–TTN5 roots and compared the localization patterns (Fig. 3P). Alexa Fluor 488-labeled anti-HA antibody staining reflected HA_3_–TTN5 localization, and signals were visible in root cells and root hairs as expected. Signals were mostly present in punctate structures close to the PM and in the cytosol (Fig. 3P), fitting the fluorescence signals obtained with YFP–TTN5.

**Fig. 4.**
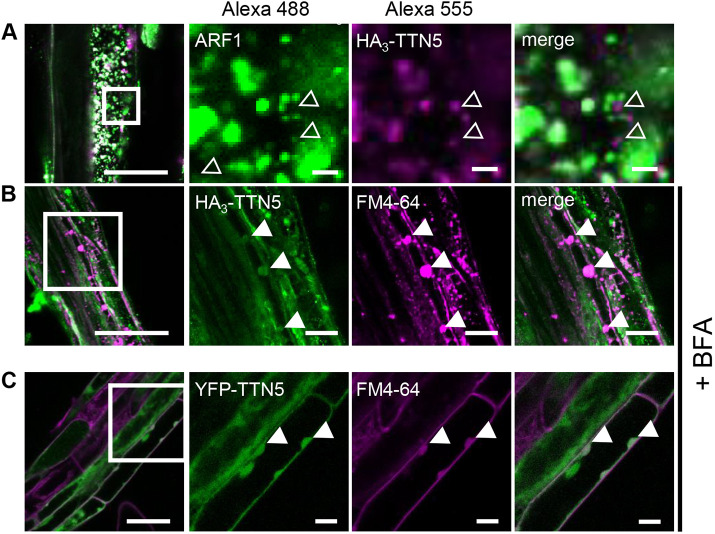
**Whole-mount immunolocalization hints at TTN5 presence in BFA bodies.** (A,B) Colocalization of HA_3_–TTN5 seedlings by whole-mount immunostaining. (A) Detection of HA_3_–TTN5 (anti-HA primary antibody, Alexa Fluor 555-labeled secondary antibody) with Golgi and TGN marker ARF1 (anti-ARF1 primary antibody, Alexa Fluor 488-labeled secondary antibody). Both fluorescence signals were detected in vesicle-like structures in root cells in close proximity to each other but mostly not colocalizing. The experiment was repeated twice with three seedlings (*n*=3). (B) Detection of HA_3_–TTN5 (anti-HA primary antibody, Alexa Fluor 488-labeled secondary antibody) and staining with membrane dye FM4-64 after BFA treatment (10 mM FM4-64 FX and 72 µM BFA for 1 h). Alexa Fluor 488 signals colocalized with FM4-64 in BFA bodies in root cells. The experiment was repeated three times with three seedlings (*n*=3). (C) YFP fluorescence in YFP–TTN5 seedlings, co-analyzed with FM4-64 after BFA treatment. YFP fluorescence signals colocalized with FM4-64 in BFA bodies similar to what was seen in B. The experiment was performed once with three independent YFP–TTN5 lines (*n*=3). Colocalization indicated by filled white arrowheads, non-colocalized HA_3_–TTN5 Alexa Fluor 488-labeled signals is indicated with empty white arrowheads. Scale bars: 50 µm (overview images on left), 10 µm (magnifications).

For a more detailed investigation of the HA_3_–TTN5 subcellular localization, we performed co-immunofluorescence staining with an Alexa Fluor 488-labeled anti-ARF1 antibody, which recognizes the Golgi and trans-Golgi network (TGN), together with Alexa Fluor 555-labeled HA_3_–TTN5 (Robinson et al., 2011; Singh et al., 2018) (Fig. 4A). ARF1–Alexa Fluor 488 staining was clearly visible in punctate structures representing presumably Golgi stacks (Fig. 4A) (Singh et al., 2018). Similar structures were seen with HA_3_–TTN5–Alexa Fluor 555 staining, but these did not colocalize with the ARF1-labeled structures, although they were in close proximity to each other (Fig. 4A). We hypothesized that the HA_3_–TTN5 structures might be connected to intracellular trafficking steps and performed brefeldin A (BFA) treatment, a commonly used tool in cell biology for preventing dynamic membrane trafficking events and vesicle transport involving the Golgi. BFA is a fungal macrocyclic lactone that leads to a loss of cis-cisternae and accumulation of Golgi stacks, known as BFA-induced compartments, and Golgi–ER fusion (Ritzenthaler et al., 2002; Wang et al., 2016). For better BFA body identification, we simultaneously used the membrane dye FM4-64, which can emit fluorescence in a lipophilic membrane environment. FM4-64 marks the PM only a few minutes after application to the cell, and can then be endocytosed; in the presence of BFA, accumulates in BFA bodies (Bolte et al., 2004). We observed BFA bodies positive for both HA_3_–TTN5–Alexa Fluor 488 and FM4-64 signals (Fig. 4B). Similar patterns were observed for YFP–TTN5-derived signals in YFP–TTN5-expressing roots (Fig. 4C). Hence, HA_3_–TTN5 and YFP–TTN5 are present in similar subcellular membrane compartments.

In HA_3_–TTN5 immunostaining, we did not observe any staining in nuclei or ER (Figs 3P, 4A,B), in contrast to the fluorescence signals in YFP–TTN5-expressing cells. This might indicate that either the nuclear and ER signals seen with YFP–TTN5 correspond to the smaller proteins detected, or that immunostaining was not suited to detect this localization. Hence, we focused on analysis of the area where there were overlapping localization patterns between fluorescence with YFP-labeled TTN5 and HA_3_-TTN5 immunostaining, such as the specific signal patterns seen for punctate membrane structures.

Taken together, our results show that signals of YFP–TTN5 and HA_3_–TTN5 were located in multiple membrane compartments in the epidermis of different *Arabidopsis* organs and of *N. benthamiana* leaves, including particular ring-like punctate structures and vesicles. Fluorescence signals in YFP–TTN5- and YFP-TTN5^Q70L^-expressing seedlings displayed high mobility in cells, as expected from an active GTPase functioning in dynamic processes, such as vesicle trafficking. In contrast, fluorescence signals for YFP–TTN5^T30N^ were less mobile, consistent with the root length phenotype conferred by HA_3_–TTN5^T30N^, speaking in favor of the observed TTN5^T30N^ kinetics, with a very fast nucleotide exchange rate and nucleotide affinity loss. Altogether, the TTN5 intracellular localization indicates that TTN5 might have multiple cellular functions as an active GTPase as it can associate with different intracellular structures of the endomembrane system.

### TTN5 might associate with components of the cellular endomembrane system

The overlapping localization of HA_3_–TTN5 and YFP–TTN5 signals prompted us to better resolve the membrane structures and compartments of the highly dynamic endomembrane system. Well-established fluorescent markers and pharmacological treatments help to determine the nature of individual components in cells in parallel to colocalization studies with proteins of interest, such as TTN5. We conducted colocalization experiments in *N. benthamiana* leaf epidermis as the fluorescence signals were comparable to those for *Arabidopsis* cotyledons and root epidermis. Moreover, it represents an established system for functional association of fluorescent proteins with multiple endomembrane components and optimal identification of membrane structures (Brandizzi et al., 2002; Hanton et al., 2009).

**Fig. 5.**
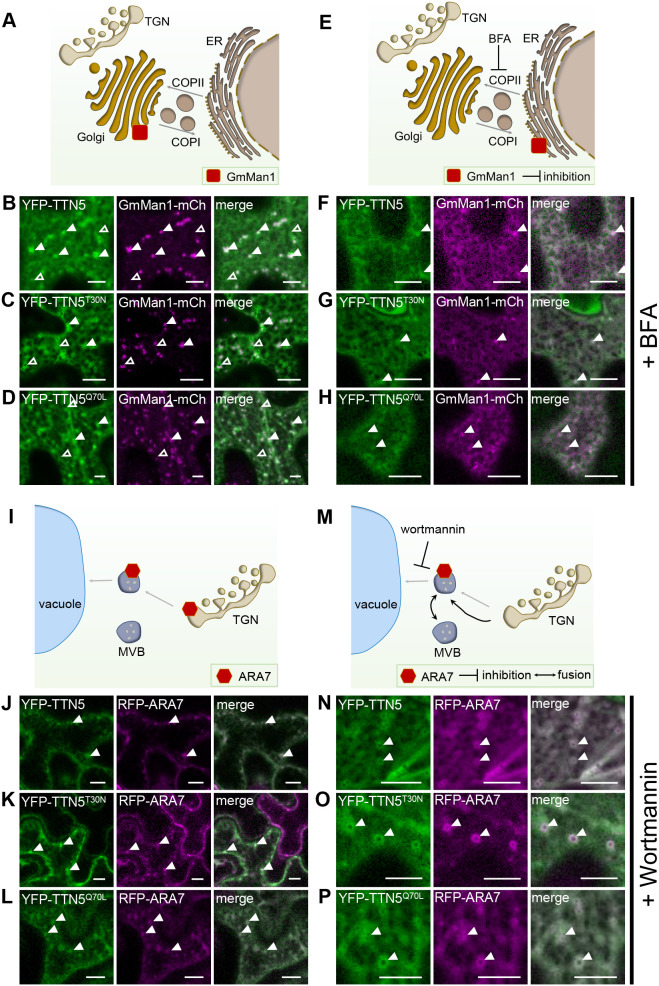
**TTN5 might be associated with the endomembrane system in *N. benthamiana* pavement cells.** YFP signals were detected in *N. benthamiana* pavement cells transiently expressing YFP–TTN5, YFP–TTN5^T30N^ and YFP–TTN5^Q70L^ with specific endomembrane markers via fluorescence confocal microscopy. (A) Schematic representation of GmMan1 localization at the cis-Golgi site. (B–D) Partial colocalization of the YFP signal with the Golgi marker GmMan1–mCherry at cis-Golgi stacks (filled white arrowheads). Additionally, YFP fluorescence signals were detected in non-colocalizing punctate structures with depleted fluorescence in the center (empty white arrowheads). (E) Schematic representation of GmMan1 localization at the ER upon BFA treatment. BFA blocks ARF GEF proteins, leading to a loss of Golgi cis-cisternae and formation of BFA-induced compartments due to an accumulation of Golgi stacks up to redistribution of the Golgi to the ER by fusion (Renna and Brandizzi, 2020). (F–H) GmMan1–mCherry and YFP fluorescence were present in the ER and in colocalizing punctate structures upon BFA treatment (36 µM for ∼30–60 min). (I) Schematic representation of ARA7 localization at the TGN and MVBs. (J–L) Colocalization of YFP signals with the MVB marker RFP–ARA7. (M) Schematic representation of ARA7 localization in swollen MVBs upon wortmannin treatment. Wortmannin inhibits PI3K function leading to TGN/EE fusion to swollen MVBs (Renna and Brandizzi, 2020). (N–P) ARA7-RFP colocalized with YFP signal in swollen MVBs upon wortmannin treatment (10 µM for ∼30–10 min). Colocalization indicated with filled arrowheads; non-colocalized YFP signal is indicated with with empty arrowheads. Corresponding colocalization analysis data is presented in Fig. S8. Experiments were repeated three times with two plants (*n*=2). Scale bars: 10 μm.

At first, we further investigated the ER–Golgi connection as a characteristic site of association with small GTPases, like the tested ARF1, involved in COPI vesicle transport (Just and Peränen, 2016). We used the soybean (*Glycine max*) protein α-1,2 mannosidase 1 (GmMan1) as a marker; this protein is a glycosidase that acts on glycoproteins at the cis-Golgi, facing the ER (Fig. 5A) and is visible as nearly round punctuate structures throughout the whole cell (Nelson et al., 2007; Wang et al., 2016). Fluorescence signals in leaf discs transiently expressing YFP–TTN5 and its mutant variants partially colocalized with GmMan1–mCherry signals at Golgi stacks (Fig. 5B–D). We also observed YFP fluorescence in form of circularly shaped ring structures with a fluorescence-depleted center; such structures can be of vacuolar origin as described for similar fluorescent rings for ANNI–GFP (Tichá et al., 2020). Furthermore, quantitative analysis reflected the visible colocalization of GmMan1 marker and YFP fluorescence with Pearson coefficients 0.63 (YFP–TTN5), 0.65 (YFP–TTN5^T30N^) and 0.68 (YFP–TTN5^Q70L^) (Fig. S8A; similar obtained overlap coefficients), indicating a strong correlation between the signals. We performed an additional object-based analysis to compare overlapping YFP fluorescence in YFP–TTN5-expressing leaves with GmMan1–mCherry signals (YFP/mCherry ratio) and vice versa (mCherry/YFP ratio). We detected 24% overlapping YFP fluorescence signals for TTN5 with Golgi stacks, whereas in YFP-TTN5^T30N^ and YFP-TTN5^Q70L^-expressing leaves, signals only shared 16 and 15% overlap with GmMan1–mCherry-positive Golgi stacks (Fig. S8B). Some YFP signals did not colocalize with the GmMan1 marker; these signals were more prominent in leaves expressing YFP–TTN5^T30N^ and less prominent for leaves expressing YFP-TTN5^Q70L^ compared to YFP–TTN5 expression (Fig. 5B–D). Indeed, we identified 48% GmMan1–mCherry signal overlapping with YFP-positive structures in YFP–TTN5^Q70L^ leaves, whereas this was 43% and 31% for YFP fluorescence signals in YFP–TTN5 and YFP–TTN5^T30N^-expressing leaves, respectively (Fig. S8B), indicating a smaller amount of GmMan1-positive Golgi stacks colocalizing with YFP signals for YFP–TTN5^T30N^. Hence, the GTPase-active TTN5 forms are likely present at cis-Golgi stacks at higher levels compared to TTN5^T30N^.

Next, we evaluated the Golgi localization by BFA treatment, resulting in a corresponding redistribution of GmMan1–mCherry (Ritzenthaler et al., 2002; Wang et al., 2016) (Fig. 5E). We found that upon BFA treatment, the GmMan1–mCherry signal was present in the ER and in BFA-induced compartments with partially matching localization of YFP signal of YFP–TTN5 constructs, suggesting a connection of TTN5 to Golgi localization (Fig. 5F–H). Hence, colocalization with GmMan1–mCherry and BFA treatment is indicative of YFP signals localizing to Golgi stacks upon YFP–TTN5 expression, whereas there was lower association of the YFP–TTN5^T30N^ mutant form with this membrane compartment.

Second, we investigated localization to the endocytic compartments, endosomes of the TGN and multivesicular bodies (MVBs) using RFP–ARA7 (RABF2B), a small RAB-GTPase present there (Kotzer et al., 2004; Lee et al., 2004; Stierhof and El Kasmi, 2010; Ito et al., 2016) (Fig. 5I). These compartments play a role in sorting proteins between the endocytic and secretory pathways, with MVBs developing from the TGN and representing the final stage in transport to the vacuole (Valencia et al., 2016; Heucken and Ivanov, 2018). Colocalization studies revealed that the YFP signal in YFP–TTN5-expressing leaves was present at RFP–ARA7-positive MVBs (Fig. 5J). Noticeably, overlaps between RFP–ARA7 and YFP fluorescence signals upon TTN5^T30N^ expression were lower than for the other TTN5 forms (Fig. 5J–L; Fig. S8C,D). We obtained a Pearson coefficient for YFP fluorescence from YFP–TTN5 or YFP–TTN5^Q70L^ expression with RFP–ARA7 of 0.78, whereas a coefficient of only 0.59 was obtained with YFP–TTN5^T30N^, confirming the visual observation (Fig. S8C; similar obtained overlap coefficients). Object-based analysis showed that, RFP–ARA7-positive structures had an overlap with YFP fluorescence in YFP–TTN5-expressing leaves of 29%, and even more with YFP–TTN5^Q70L^ (75%) unlike with YFP–TTN5^T30N^ (21%) (Fig. S8D). Based on this, signals of YFP–TTN5^Q70L^ and YFP–TTN5 tended to colocalize better with ARA7-positive compartments than YFP–TTN5^T30N^.

To test MVB localization, we treated plant cells with wortmannin, a fungal metabolite that inhibits phosphoinositide 3-kinase (PI3K) function and thereby causes swelling of the MVBs (Cui et al., 2016), a common approach to study endocytosis events (Fig. 5M). RFP–ARA7-expressing cells showed the typical wortmannin-induced formation of doughnut-like shaped MVBs (Jaillais et al., 2008). YFP fluorescence in YFP–TTN5-expressing leaves partially colocalized with these structures (Fig. 5N–P) indicating that fluorescence signals from expression of YFP–TTN5 and its two mutants are present in MVBs. YFP signals in YFP–TTN5^Q70L^-expressing leaf discs were located even to a greater extent to MVBs than for YFP–TTN5 and much more than for YFP–TTN5^T30N^-expressing cells, suggesting an active role for YFP–TTN5^Q70L^ in MVBs, for example in the lytic degradation pathway or the recycling of proteins, similar to the role of ARA7 (Kotzer et al., 2004).

**Fig. 6.**
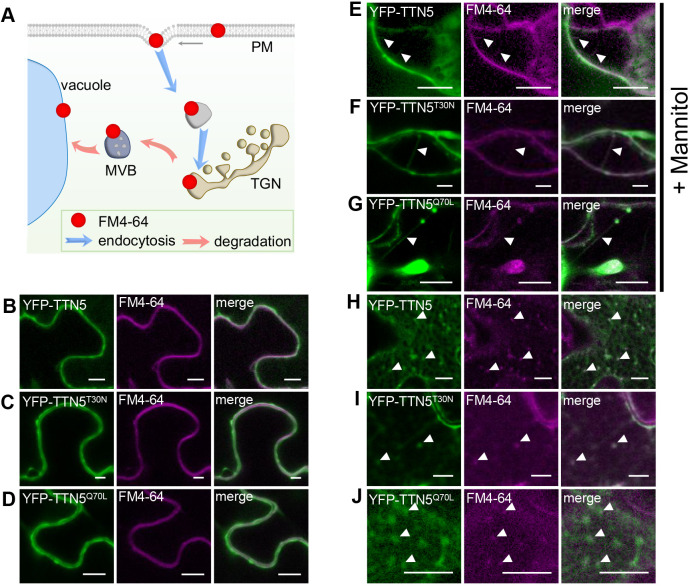
**TTN5 might colocalize with endocytosed PM material.** (A) Schematic representation of the progressive stages of lipophilic membrane dye FM4-64 localization and internalization in cells. After infiltration, it first localizes in the PM, and later in intracellular vesicles and membrane compartments, reflecting the endocytosis process (Bolte et al., 2004). (B–J) YFP fluorescence colocalized with FM4-64 in *N. benthamiana* leaf epidermal cells as observed by confocal microscopy, following transient expression of YFP–TTN5, YFP–TTN5^T30N^ and YFP–TTN5^Q70L^. (B–D) YFP signals colocalized with FM4-64 at the PM. (E–G) PM localization of YFP fluorescence was evaluated after mannitol-induced plasmolysis (1 M for ∼15–30 min). Formation of Hechtian strands is a sign of PM material and fluorescence staining there (filled arrowheads). (H–J) Internalized FM4-64 was present in vesicle-like structures that showed YFP signals. Colocalization indicated with filled arrowheads. Experiments were repeated three times with two plants (*n*=2). Scale bars: 10 μm.

Finally, to investigate a possible connection of TTN5 with the PM, we determined the colocalization of YFP signals from YFP–TTN5 constructs with FM4-64 (Fig. 6A). Fluorescence signals for all three YFP–TTN5 forms colocalized with FM4-64 at the PM in a similar manner (Fig. 6B–D). To further investigate PM localization, we performed mannitol-induced plasmolysis. YFP signals for all YFP–TTN5 constructs were located similarly to FM4-64-stained Hechtian strands, thread-like structures attached to the apoplast visible upon plasmolysis and surrounded by PM (Fig. 6E–G).

In summary, these colocalization experiments show that YFP signals upon YFP–TTN5 expression are found in different membrane sites of the endomembrane system, including the Golgi, MVBs and PM. We hypothesize that, similar to other ARF proteins, this pattern indicates that TTN5 participates in a highly dynamic vesicle trafficking process. Indeed, recorded dynamic YFP signal movement of YFP–TTN5 and YFP–TTN5^Q70L^ in *N. benthamiana* pavement cells colocalized with GmMan1–mCherry signals, and revealed high motion over time, whereas this was less the case for the YFP–TTN5^T30N^ construct (Movies 13–15).

One potential cellular trafficking route is the degradation pathway to the vacuole. We, therefore, investigated fluorescence localization upon transient expression of YFP–TTN5 together with FM4-64 in late endosomal compartments, which might be involved in vacuolar targeting. FM4-64 is used as a marker for membranes of late endosomal compartment and vacuole targeting, because following PM visualization FM4-64-stained endocytic vesicles become apparent at later stages, as well as tonoplast staining (Ueda et al., 2001; Emans et al., 2002; Dhonukshe et al., 2007; Ivanov and Vert, 2021). Next to YFP colocalization in YFP–TTN5-expressing leaves with FM4-64 at the PM, we detected colocalization with intracellular fluorescent compartments; a similar expression was seen for both mutant forms (Fig. 6H–J). This indicates that YFP–TTN5 might be involved in targeting of endocytosed PM material, irrespective of the mutations.

In summary, YFP signals upon YFP–TTN5 and YFP–TTN5^Q70L^ expression were dynamic and colocalized with endomembrane structures, whereas fluorescence signal in YFP–TTN5^T30N^-expressing leaf discs tended to be less mobile and dynamic and colocalized less with such structures.

## DISCUSSION

**Fig. 7.**
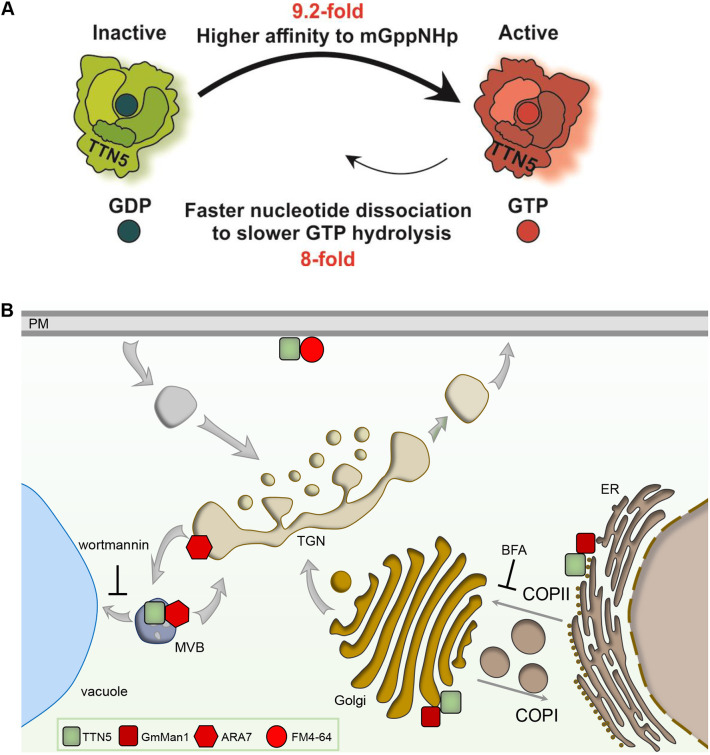
**Schematic models summarizing TTN5 kinetic GTPase activities and potential localization within cells.** (A) Model of the predicted GTPase nucleotide exchange and hydrolysis cycle of TTN5 based on the biochemical investigation. The TTN5 affinity for mGppNHp is 9.2-fold higher than it is for mGDP resulting in fast switching from inactive GDP-loaded to active GTP-loaded form. mGppNHp dissociation is 8-fold faster than GTP hydrolysis, but both processes were much slower than nucleotide association. TTN5 kinetics identified TTN5 as a non-classical GTPase that tends to stay in a GTP-loaded form even under resting conditions. (B) Presumed TTN5 locations within the cell. TTN5 (green square) can be present at the PM similar as FM4-64 (red circle) or in the endomembrane compartments of the TGN or MVB as found by ARA7-colocalization (red hexagon). Additionally, TTN5 might colocalize with GmMan1-positive (red square) Golgi stacks.

This work provides evidence that the small ARF-like GTPase TTN5 has very rapid intrinsic nucleotide exchange capacity with a conserved nucleotide-switching mechanism. TTN5 might primarily be present in a GTP-loaded active form in cells as a dynamic protein with respect to its localization to membrane structures, potentially associating it with vesicle transport and different endomembrane processes (Fig. 7). The active TTN5^Q70L^ mutant was capable of nucleotide switching and appeared to be mostly similarly localized to wild-type TTN5. The TTN5^T30N^ mutant, on the other hand, has a lower nucleotide exchange capacity, and differed significantly in localization properties and its dynamics, albeit depending on cell types, and conferred a root length phenotype. Therefore, the GTP-bound state that we presume for TTN5 is most likely crucial for correct protein localization and dynamics.

### TTN5 exhibits characteristic GTPase functions

TTN5 is classified as an ARL2 homolog based on its high sequence identity with human HsARL2 (McElver et al., 2000), which is reinforced by structural prediction of a nucleotide-binding pocket. TTN5, TTN5^T30N^ and TTN5^Q70L^ can all bind guanine nucleotides. *k*_on_ values for TTN5^T30N^ and TTN5 were nearly identical, indicating that the mutation has no effect on GDP-binding characteristics, as would usually expected in the absence of a GEF. The TTN5^Q70L^ *k*_on_ value was clearly higher compared to that of the wild type, indicating that this mutant can bind GDP faster. Compared to other Ras superfamily members, these values are in the range of HRAS (Hanzal-Bayer et al., 2005) and around ten times slower than the fast association of RAC1 (Jaiswal et al., 2013). Well-studied RAS proteins, like RAC1, RAC2 and RAC3, have an intrinsic nucleotide exchange reaction rates of ∼40,000 s^−1^ (Haeusler et al., 2006), whereas TTN5 has a remarkably fast rate of nucleotide exchange that is very similar to that of HsARL2 (Hanzal-Bayer et al., 2005; Veltel et al., 2008). This suggests that TTN5 quickly replaces GDP for GTP and transforms from an inactive to an active state presumably without the need of GEF interaction. This could also be an explanation for what is seen with TTN5^Q70L^. Small GTPases with mutations in the glutamine residue of switch II region (e.g. Q71 for HsARF1 and ARL1, and Q61 for HRAS) are constitutively active (Zhang et al., 1994; Van Valkenburgh et al., 2001; Karnoub and Weinberg, 2008). Accordingly, TTN5^Q70L^ is likely to exchange GDP rapidly to GTP and switch itself to stay in an active form, as suggested by the fast-intrinsic nucleotide exchange rate. Interestingly, TTN5^T30N^ resulted in an even higher dissociation rate constant (*k*_off_.). The calculated *K*_d_ confirmed the higher nucleotide-binding affinity for GDP of TTN5 and TTN5^Q70L^ compared with TTN5^T30N^. Reports on HsARL2, HsARF6 and HsARL4D show that their corresponding T30N mutants led to a similar decreased affinity for GDP (Macia et al., 2004; Hanzal-Bayer et al., 2005; Li et al., 2012).

Interestingly, a comparison of mdGDP with mGppNHp revealed that all three versions had higher GTP affinity than with GDP, with the highest for TTN5^Q70L^. These high GTP affinities in combination with fast GDP exchange rates and extremely slow hydrolysis pinpoint TTN5 as being GTP-loaded, even in resting conditions, which is very unusual. This atypical behavior has already been reported for a few non-classical RHO GTPases like RHOD and RIF (Jaiswal et al., 2013). This unusual GTP-bound active state along with it lacking *N*-myristoylation and the phylogenetic distance (Boisson et al., 2003; Vernoud et al., 2003) strengthens that there are major differences between TTN5 and other ARF proteins. The similarity in binding affinity between wild-type and TTN5^Q70L^ is consistent with a previous report on HsARL2 (Hanzal-Bayer et al., 2005). Additionally, an equivalent ratio of nucleotide affinity was found between HRAS and HRAS^Q61L^, but with a much higher affinity, more typical for small GTPases (Der et al., 1986). Given that Q70 is important for GAP-stimulated GTP hydrolysis (Cherfils and Zeghouf, 2013), we assume nucleotide exchange activity is unaffected.

The *Arabidopsis* genome encodes only the two large of five mammalian ARF-GEF subgroups, BFA-inhibited GEF (BIG) and the Golgi BFA-resistance factor 1 (GBF/GNOM) family (Memon, 2004; Wright et al., 2014; Brandizzi, 2018), but no TTN5 GEF protein has been reported. Potential interactions with GEFs are of high interest given the potential role of TTN5 as a co-GEF, similar to what is seen for HsARL3 and HsARL2, where their effector BART stabilizes the active GTPase (ElMaghloob et al., 2021). Especially, interactions at the nucleotide-binding site, which are prevented in the TTN5^T30N^ mutant, will be of great interest to study further for TTN5.

Taken together, the categorization as a non-classical GTPase has three implications. First, very slow hydrolysis rates predict the existence of a TTN5 GAP. Second, TTN5^T30N^ might function as a dominant-negative mutant and, in the presence of a GEF, it cannot bind GDP. Third, the TTN5^Q70L^ hydrolysis rate is not decreased.

### TTN5 might act in the endomembrane system

The localization data on YFP– and HA_3_–TTN5 suggest that it might be localized at different cellular membrane compartments, typical for the ARF-like family (Memon, 2004; Sztul et al., 2019), and supports potential involvement of TTN5 in endomembrane trafficking. Even though YFP–TTN5 did not complement the *ttn5-1* embryonic lethality, we made several observations that suggest that the YFP–TTN5 signals seen at various membrane sites are meaningful. YFP–TTN5 might not complement due to differences in TTN5 levels and interactions in some cell types, which were hindered specifically for YFP–TTN5 but not HA_3_–TTN5. In a previous study, overexpression of ARF1 did not affect the intracellular localization compared to endogenous tagged-ARF1 but did cause the formation of tubulated structures (Bottanelli et al., 2017). Alhough constitutively driven, YFP–TTN5 expression might be delayed or insufficient at early embryonic stages resulting in the lack of embryonic lethal complementation. On the other hand, the fast nucleotide exchange activity might be hindered by the large YFP compared to the small HA_3_ tag, given that HA_3_–TTN5 rescued the embryo lethality. The lack of complementation represents a challenge for determining the localization of small GTPases with rapid nucleotide exchange in plants. Despite these limitations, we made relevant observations that made us believe that YFP signals in YFP–TTN5-expressing cells at membrane sites are meaningful. First, using pharmacological treatments and colocalization with organellar markers, we noted that various particular membrane compartments showed YFP signals, such as the punctate small ring-like structures, resembling previously reported ANNI–GFP staining (Tichá et al., 2020), the large wortmannin-induced ring-like structures and the BFA bodies, all of which are meaningful for vesicle transport and PM protein regulation processes (Wang et al., 2009; Suo et al., 2021). Furthermore, fluorescence signals obtained with YFP–TTN5 constructs also depended on T30 and Q70 residues; particularly YFP–TTN5^T30N^ had partly quite distinct fluorescence localization patterns, reduced mobility in certain cells and differing degrees of colocalization with the utilized markers. Next to this, HA_3_–TTN5^T30N^ seedlings showed reduced root growth which might be due to similar reasons. Given that TTN5^T30N^ has a very fast nucleotide exchange rate and less affinity to nucleotides compared to TTN5, these differing YFP fluorescence patterns of YFP–TTN5^T30N^ at membrane sites and the effect on root growth are not unexpected. Hence, we considered these specific YFP localizations at membrane sites as valid, especially when supported by HA_3_–TTN5 immunodetection.

Following up, colocalization analysis showed that both cis-Golgi and MVB-positive structures colocalized with a higher proportion with YFP signals from YFP–TTN5^Q70L^ than from YFP–TTN5^T30N^. This could indicate the site of TTN5 action, considering our knowledge of ARF family activation in other organisms with high TTN5 sequence similarity. Small GTPases are usually recruited or move to their place of action upon interaction with their specific GEF and nucleotide exchange-dependent activation (Sztul et al., 2019; Nielsen, 2020; Adarska et al., 2021). Although our biochemical data implies no need for a typical GTPase–GEF interaction for activation, it can be still important for localization. Most of effector–GTPase interactions take place with GTPases in their GTP-bound form (Sharer and Kahn, 1999; Hanzal-Bayer et al., 2005). One exception is the role of ARL2–Alp41-GDP in microtubule dynamics by interaction with Cofactor D–Alp1^D^ (Bhamidipati et al., 2000; Mori and Toda, 2013). Another possibility is a hindrance of dimerization by the T30N mutation. ARF1 protein dimer formation is important for the formation of free vesicles (Beck et al., 2009; Beck et al., 2011) and is associated with cell mobility, which was disturbed in YFP–TTN5^T30N^-expressing cells. Colocalization of YFP fluorescence upon YFP–TTN5 expression with ARA7-positive structures, even in the wortmannin-induced swollen state, might indicate that TTN5 has similar functions to ARA7. ARA7 is involved in endocytic cargo transport to the vacuole, for example, endocytosis of PM material (Ueda et al., 2001; Sohn et al., 2003; Kotzer et al., 2004; Ebine et al., 2011). Colocalization of FM4-64-labeled intracellular structures with fluorescence in YFP–TTN5-expressing cells might indicate the TTN5 has a role in endocytosis and the possible degradation pathway into the vacuole. Our data on colocalization with the different markers support the hypothesis that TTN5 might have functions in vesicle trafficking.

A potential explanation of YFP localization to similar compartments upon YFP–TTN5 and YFP–TTN5^Q70L^ expression compared to fluorescence signal of YFP–TTN5^T30N^ expression can be made based on a special feature of TTN5 in the ARF family. ARF GTPases are mostly myristoylated on G2, which is essential for their membrane binding. TTN5, as well as HsARL2 and HsARL3, lack this myristoylation, although G2 is present (Boisson et al., 2003; Kahn et al., 2006). HsARL2 and HsARL3 are still able to bind membranes, probably only through their N-terminal amphipathic helix, as was established for SAR1, with HsARL2 membrane binding efficiency being nucleotide independent (Lee et al., 2005; Kapoor et al., 2015). We suggest similar behavior for TTN5, as detected YFP signals localized to membranous compartments. Based on the varying colocalization degrees, with signals of YFP–TTN5^T30N^ construct being less prominent at the Golgi and MVBs compared to YFP–TTN5 and YFP–TTN5^Q70L^, we hypothesize that different membrane localization could be associated with a nucleotide- or nucleotide exchange-dependent process. In a nucleotide-free or GDP-bound state, TTN5 might be predominantly present close to the PM, whereas in an active GTP-bound state, which according to enzyme kinetics is expected to be predominant, it would be dynamically linked with the endomembrane system. Interestingly, with respect to the intracellular dynamics, we observed that the TTN5^T30N^ mutant had a different behavior in different organ types. This could be due to differing GEFs. Likewise, it is conceivable that constitutively expressed TTN5 has different effector binding partners.

The broad diversity of biological functions for proteins that have related sequences to TTN5, such that they are associated with a variety of signaling cascades, is also reflected by these related proteins having very different protein partners. Few orthologs of HsARL2 interaction partners are present in *Arabidopsis*. It is therefore exceedingly interesting to identify interacting proteins to determine whether TTN5 performs similar functions to HsARL2 or what other role it might play, especially with regard to the potential GTP dependence of TTN5 essential function, which fits with already known functions of other ARF GTPases (Sztul et al., 2019; Nielsen, 2020; Adarska et al., 2021). In addition, ARF proteins are affected by a similar set of GEFs and GAPs, indicating an interconnected network of ARF signaling. ARF double knockdowns have revealed specific phenotypes, suggesting redundancy in the ARF family (Volpicelli-Daley et al., 2005; Kondo et al., 2012; Nakai et al., 2013; Adarska et al., 2021). Investigation of the role of TTN5 within the ARF family might reveal a missing link in ARF signaling and cell traffic.

### Conclusion

In this study, we identified TTN5 as a functional ARF-like GTPase that not only shares sequence similarity with HsARL2, but also the very fast nucleotide exchange capacity, in contrast to other characterized ARF and ARL proteins. TTN5 has a fast nucleotide dissociation and a slow GTP hydrolysis rate, and a higher affinity for GTP than GDP. Thus, TTN5 is a non-classical GTPase that most likely accumulates in a GTP-bound state in cells in line with certain cellular phenotypes and protein localization data. The nucleotide exchange capacity affected the localization and dynamics of YFP-tagged TTN5 forms, and the association of TTN5 with the endomembrane system. In the future, identification of a potential TTN5 GEF, GAP and effector proteins as well as other interaction partners, and particularly potential PM target proteins as cargo for vesicle transport, will be of great interest to clarify potential roles of TTN5 in endomembrane trafficking and whole-plant physiological contexts.

## MATERIALS AND METHODS

### *Arabidopsis* plant material and plant growth conditions

The *Arabidopsis ttn5-1* (Stock Number: CS16077) mutant was previously described (McElver et al., 2000). Heterozygous seedlings were selected by genotyping using the primers TTN5 intron1 fwd and pDAP101 LB1 (Table S1). For pro35S::YFP-TTN5 and pro35S::HA_3_-TTN5 constructs, *TTN5* coding sequences was amplified with B1 and B2 attachment sites for Gateway cloning (Life Technologies) using the primer TITAN5 n-ter B1 and TITAN5 stop B2 (Table S1). The obtained PCR fragments were cloned via BP reaction (Life Technologies) into pDONR207 (Invitrogen). pro35S::YFP-TTN5 and pro35S::HA_3_-TTN5 constructs were created via LR reaction (Life Technologies) with the destination vector pH7WGY2 (Karimi et al., 2005) (VIB-UGent Center for Plant Systems Biology, Vector ID:1_48) and pALLIGATOR2 (Bensmihen et al., 2004), respectively. Agrobacteria were transformed with obtained constructs and used for stable *Arabidopsis* transformation (method adapted from Clough and Bent, 1998). *Arabidopsis* seeds were sterilized with sodium hypochlorite solution (6% sodium hypochlorite and 0.1% Triton X-100) and stored for 24 h at 4°C for stratification. Seedlings were grown upright on half-strength Hoagland agar medium [1.5 mM Ca(NO_3_)_2_, 0.5 mM KH_2_PO_4_, 1.25 mM KNO_3_, 0.75 mM MgSO_4_, 1.5 µM CuSO_4_, 50 µM H_3_BO_3_, 50 µM KCl, 10 µM MnSO_4_, 0.075 µM (NH_4_)_6_Mo_7_O_24_, 2 µM ZnSO_4_, 50 μM FeNaEDTA and 1% sucrose, pH 5.8, supplemented with 1.4% Plant agar (Duchefa)] in growth chambers (CLF Plant Climatics) under long-day conditions (16 h light at 21°C, 8 h darkness at 19°C). Seedlings were grown for 6 days (6-day system) or 10 days (10-day system) or 17 days with the last 3 days on fresh plates (2-week system).

Seed clearing was undertaken by incubating seeds in a chloral hydrate-glycerol clearing solution [chloral hydrate:glycerol:water, 8:1:2 (g:ml:ml)] for 4 h up to overnight. Imaging was done using an Axio Imager.M2 (Zeiss).

Root length measurement were performed using JMicroVision with the image analysis toolbox for measuring and quantifying components of high-definition images [version 1.3.4; https://jmicrovision.github.io; Roduit, N.]

*Nicotiana benthamiana* plants were grown on soil for 2–4 weeks in a greenhouse facility under long-day conditions (16 h of light, 8 h of darkness).

### Point mutant generation of *TTN5*

pDONR207:TTN5 was used as a template for site-directed *TTN5* mutagenesis. Primers T5T30Nf and T5T30Nr (Table S1) were used to amplify the entire vector generating the TTN5^T30N^ coding sequence and primers TQ70Lf and T5Q70Lr (Table S1) were used to amplify the entire vector generating the TTN5^Q70L^ coding sequence. The PCR amplifications were run using the following conditions: 95°C, 30 s; 18 cycles of 95°C for 30 s, 55°C for 1 min, and 72°C for 8 min; then 72°C for 7 min. The completed reaction was treated with 10 units of DpnI endonuclease for 1 h at 37°C and then used for *Escherichia coli* transformation. Successful mutagenesis was confirmed by Sanger sequencing.

### *In vitro* GTPase activity assays

An overview of protein expression and purification is shown in Fig. S2A. Recombinant pGEX-4T-1 bacterial protein expression vectors (Amersham, Germany) containing coding sequences for *TTN5*, *TTN5^T30N^* and *TTN5^Q70L^* were transferred into *E. coli* BL21 (DE3) Rosetta strain (Invitrogen, Germany). Following induction of GST–TTN5 fusion protein expression according to standard procedures (Hemsath et al., 2005), cell lysates were obtained after cell disruption with a probe sonicator (Bandelin sonoplus ultrasonic homogenizer, Germany) using a standard buffer [300 mM NaCl, 3 mM dithiothreitol (DTT), 10 mM MgCl_2_, 0.1 mM GDP, 1% glycerol and 50 mM Tris-HCl, pH 7.4]. GST fusion proteins were purified by loading total bacterial lysate on a preequilibrated glutathione Sepharose column (Sigma, Germany) using fast performance liquid chromatography system (Cytiva, Germany) (Step 1, affinity-purified GST-TTN5 protein fraction). GST-tagged protein fractions were incubated with thrombin (Sigma, Germany) at 4°C overnight for cleavage of the GST tag (Step 2, GST cleavage) and applied again to the affinity column (Step 3, yielding TTN5 protein fraction). Purified proteins were concentrated using 10 kDa ultra-centrifugal filter Amicon (Merck Millipore, Germany). The quality and quantity of proteins were analyzed by SDS-PAGE (Bio-Rad), a UV/Vis spectrometer (Eppendorf, Germany) and high-performance liquid chromatography (HPLC) using a reversed-phase C18 column (Sigma, Germany) and a pre-column (Nucleosil 100 C18, Bischoff Chromatography) as described previously (Eberth and Ahmadian, 2009) (Fig. S2B–D).

Nucleotide-free TTN5 protein was prepared from the TTN5 protein fraction (Eberth and Ahmadian, 2009) as illustrated in Fig. S2E. 0.5 mg TTN5 protein was combined with 1 U of agarose bead-coupled alkaline phosphatase (Sigma-Aldrich, Germany) for degradation of bound GDP to GMP and Pi in the presence of a 1.5-fold molar excess of non-hydrolyzable GTP analog GppCp (Jena Bioscience, Germany). After confirmation of GDP degradation by HPLC, 0.002 U snake venom phosphodiesterase (Sigma-Aldrich, Germany) per mg TTN5 was added to cleave GppCp to GMP, G and Pi. The reaction progress of degradation of nucleotides was analyzed by HPLC using 30 µM TTN5 in a 30 µl injection volume (Beckman Gold HPLC, Beckman Coulter). After completion of the reaction, in order to remove the agarose bead-coupled alkaline phosphatase, the solution was centrifuged for 10 min at 10,000 ***g***, 4°C, which was followed by snap freezing and thawing cycles to inactivate the phosphodiesterase. mdGDP (2-deoxy-3-O-N-methylanthraniloyl GDP)- and mGppNHp 2′/3′-O-(N-methyl-anthraniloyl)-guanosine-5′-[(β,γ)-imido]triphosphate)-bound TTN5, TTN5^T30N^ and TTN5^Q70L^ were prepared by incubation of nucleotide-free forms with fluorescent nucleotides (Jena Bioscience, Germany) in a molar ratio of 1 to 1.2. The solution was purified from the excess amount of mdGDP and mGppNHp by using prepacked gel-filtration NAP-5 Columns (Cytiva, Germany) to remove unbound nucleotides. Protein and nucleotide concentration were determined using the Bradford reagent (Sigma-Aldrich, Germany) and HPLC, respectively.

All kinetic fluorescence measurements including nucleotide association and dissociation reactions were monitored on a stopped-flow instrument system SF-61, HiTech Scientific (TgK Scientific Limited, UK) and SX20 MV (Applied Photophysics, UK) at 25°C using nucleotide exchange buffer (10 mM K_2_HPO_4_/KH_2_PO_4_, pH 7.4, 5 mM MgCl_2_, 3 mM DTT, 30 mM Tris-HCl, pH 7.5) (Eberth and Ahmadian, 2009). Fluorescence was detected at 366 nm excitation and 450 nm emission using 408 nm cut-off filter for mant-nucleotides (Hemsath and Ahmadian, 2005).

To determine the intrinsic nucleotide exchange rate, *k*_off_, 0.2 µM mdGDP- and mGppNHp-bound proteins were combined with a 200-fold molar excess of 40 µM non-fluorescent GDP in two different set of experiments, respectively. The decay of the fluorescence intensity representing mdGDP and mGppNHp dissociation and replacement by non-fluorescent nucleotide were recorded over time (Fig. S2G). Moreover, to determine the nucleotide association rate, *k*_on_, of mdGDP and mGppNHp to the nucleotide-free GTPase, 0.2 µM fluorescent nucleotides were mixed with different concentrations of nucleotide-free TTN5 variants. The increase in the fluorescent intensity was obtained by analyzing the conformational change of fluorescent nucleotides after binding to the proteins (Fig. S2H).

The data provided by the stopped-flow assay, were applied to obtain the observed rate constants. Dissociation rate constants or nucleotide exchange rates (*k*_off_ in s^−1^) and pseudo-first-order rate constants or observed rate constants (*k*_obs_ in s^−1^) at the different concentrations of the protein were obtained by non-linear curve fitting using Origin software (version 2021b). The slopes obtained from plotting *k*_obs_ against respective concentrations of proteins were used as the second-order association rate constants (*k*_on_ in µM^−1^s^−1^). The equilibrium constant of dissociation (*K*_d_ in µM) was calculated from the ratio of *k*_off_/*k*_on_. In order to investigate the intrinsic GTP-hydrolysis rate of TTN5 variants, the HPLC method is used as described previously (Eberth and Ahmadian, 2009). As an accurate strategy, HPLC provides the nucleotide contents over time. The GTPase reaction rates were determined by mixing 100 μM nucleotide-free GTPase and 100 μM GTP at 25°C in a standard buffer without GDP. The GTP contents were measured at different times and the data were fitted with Origin software to get the observed rate constant.

### *Nicotiana benthamiana* leaf infiltration

*N. benthamiana* leaf infiltration was performed with the Agrobacterium (*Agrobacterium radiobacter*) strain C58 (GV3101) carrying the respective constructs for confocal microscopy (LSM 780, Zeiss). Agrobacteria cultures were grown overnight at 28°C, centrifuged for 5 min at 4°C at 5000 ***g***, resuspended in infiltration solution (5% sucrose, a pinch of glucose, 0.01% Silwet Gold, 150 µM Acetosyringone) and incubated for 1 h at room temperature. The bacterial suspension was adjusted to an OD_600=_0.4 and infiltrated into the abaxial side of *N. benthamiana* leaves.

### Subcellular localization of fluorescent protein fusions

Cloning of YFP-tagged TTN5 constructs is described in the section ‘*Arabidopsis* plant material and growth conditions’ above. Localization studies were carried out by laser-scanning confocal microscopy (LSM 780 or LSM880, Zeiss) with a 40× C-Apochromat water immersion objective. YFP constructs and Alexa Fluor 488 stainings were excited at 488 nm and detected at 491–560 nm. mCherry, Alexa 555 and FM4-64 fluorescence was excited at 561 nm and detected at 570–633 nm.

Wortmannin (10 µM, Sigma-Aldrich), BFA (36 µM, Sigma-Aldrich) and plasma membrane dyes FM4-64 (165 µM, Thermo Fisher Scientific) were infiltrated into *N. benthamiana* leaves or used for incubation bath of *Arabidopsis* seedlings. FM4-64 was detected after 5 min incubation. Wortmannin and BFA were incubated for 25 min before checking the treatment effect. Plasmolysis was induced by incubating leaf discs in 1 M mannitol solution for 15 min. Signal intensities were increased for better visibility.

RFP–ARA7 clones were a gift from Dr Thierry Gaude (Ecole Normale Supérieure, Lyon, France).

### Whole-mount immunostaining

Whole-mount immunostaining by immunofluorescence was performed according to Pasternak et al. (2015). Briefly, *Arabidopsis* seedlings were grown in the standard condition in Hoagland medium for 4–6 days. Methanol or formaldehyde (4%) was used to fix the seedlings. The seedlings were transferred to a glass slide and resuspended in 1× microtubule-stabilizing buffer (MTSB). Seedlings were digested with 2% Driselase dissolved in 1× MTSB at 37°C for 40 mins. Following digestion, a permeabilization step was performed by treating the seedlings with permeabilization buffer (3% IGEPAL C630, 10% DMSO in 1× MTSB buffer) at 37°C for 20 mins. Then blocking was performed with a buffer consisting of 5% BSA for 30 min at room temperature. They were incubated overnight with different primary antibodies (detailed information is listed below). After two washes with 1× MTSB, seedlings were incubated with a respective Alexa Fluor secondary antibody for 2 h at 37°C. After five steps of washing with 1x PBS, coverslips were mounted on slides with the antifade reagent (Prolong glass Antifade Mountant with NucBlue Stain, Invitrogen, P36985). Fluorescence microscopy was conducted as described in the previous section.

Immunodetection was conducted with following antibody combinations: HA detection was performed using anti-HA antibody (1:100 dilution, rabbit Abcam ab9110 or chicken AGRISERA, AS20 4463, Lot: 2303) followed by Alexa Fluor 488 or Alexa Fluor 555-labeled secondary antibodies (1:200 anti-rabbit-IgG, Thermo Fisher Scientific, A32731, Lot: 2541675 and 1:500 goat anti-chicken-IgY, Thermo Fisher Scientific A32932). ARF1 (Golgi and TGN marker) was detected using primary anti-ARF1 antibody (1:200 dilution, rabbit, Agrisera, AS08 325, Lot: 2208), in combination with Alexa Fluor™ Plus 488-labeled secondary antibody.

For initiation of BFA bodies, seedlings were first treated with BFA (72 µM, Sigma-Aldrich) and fixable plasma membrane dye FM4-64 FX (10 mM, Thermo Fisher Scientific, F34653) for 1 h, before formaldehyde fixation.

### Immunoblot detection

After total protein extraction from *Arabidopsis* plants grown for 6 days or in the 2-week system, sample separation by SDS-PAGE and immunodetection were performed as previously described (Le et al., 2015). In brief, plant material was ground under liquid nitrogen and proteins were extracted with SDG buffer (62 mM Tris-HCl, pH 8.6, 2.5% SDS, 2% DTT, 10% glycerol). Samples were separated on 12% SDS-PAGE gels. Following electrophoresis, the proteins were transferred to a Protran nitrocellulose membrane (Amersham).

Membranes were blocked for 1 h in 5% milk-TBST solution (20 mM Tris-HCl, pH 7.4, 180 mM NaCl and 0.1% Tween 20), followed by 1 h antibody incubation (anti-GFP, monoclonal mouse antibody, Roche, catalog no. 11814460001, 1:1000). After three washes with TBST for 10 min each, membranes were incubated in secondary antibody (anti-mouse-IgG conjugated to HRP, polyclonal goat antibody, Sigma-Aldrich, cat. no. SAB3701159, 1:5000) for 1 h. HA detection was performed with a directly coupled anti-HA antibody (anti-HA–HRP, high-affinity monoclonal rat antibody, 3F10, Roche, catalog no. 12013819001, 1:1000). Immunodetection was performed after three washes with TBST for 10 min each, using the enhanced chemiluminescence system (GE Healthcare) and the FluorChem Q System for quantitative western blot imaging (ProteinSimple) with the AlphaView software. Full images of uncropped western blot from this study can be found in Fig. S9.

### JACoP based colocalization analysis

Colocalization analysis was carried out with the ImageJ (Schneider et al., 2012) Plugin Just Another Colocalization Plugin (JACoP) (Bolte and Cordelières, 2006) and a comparison of Pearson's and Overlap coefficients was performed. Object-based analysis was performed for punctate structures (method adapted from Ivanov et al., 2014). Colocalization for both channels was calculated based on the distance between geometrical centers of signals and presented as a percentage. Analysis was done in three replicates each (*n*=3).

### Structure prediction

TTN5 structure prediction was performed by AlphaFold (Jumper et al., 2021). The molecular graphic was edited with UCSF ChimeraX (1.2.5, Goddard et al., 2018), developed by the Resource for Biocomputing, Visualization and Informatics at the University of California, San Francisco, with support from the National Institutes of Health R01-GM129325 and the Office of Cyber Infrastructure and Computational Biology, National Institute of Allergy and Infectious Diseases.

### *In silico* tool for gene expression analysis

RNA-seq data was analyzed from previously published studies and was visualized with the AtGenExpress eFP at https://bar.utoronto.ca/eplant/ (Nakabayashi et al., 2005; Schmid et al., 2005; Ryu et al., 2019; Waese et al., 2017).

### Accession numbers for sequence data

Sequence data used in this article can be found in the TAIR and GenBank data libraries under accession numbers: *ARA7* (TAIR: AT4G19640), *ARF1* (TAIR: AT1G23490), *GmMan1* (Uniprot: Q0PKY2) and *TTN5* (TAIR: AT2G18390).

### Statistical analysis

One-way ANOVA was used for statistical analysis and performed in OriginPro 2019. Fisher's least significant difference or a Tukey's test was chosen as a post-hoc test with *P*<0.05.

## Supplementary Material



10.1242/joces.262315_sup1 Supplementary information

Table S1. Primer list.
