# Supplementary material for: Characterization of the small Arabidopsis thaliana GTPase and ADP-ribosylation factor-like 2 protein TITAN 5
Source: J Cell Sci. 2024 Aug 14;137(15):jcs262315. doi: 10.1242/jcs.262315 (PMC11361645; doi:10.1242/jcs.262315)
Supplement: Supplementary information [file joces-137-262315-s1.pdf]

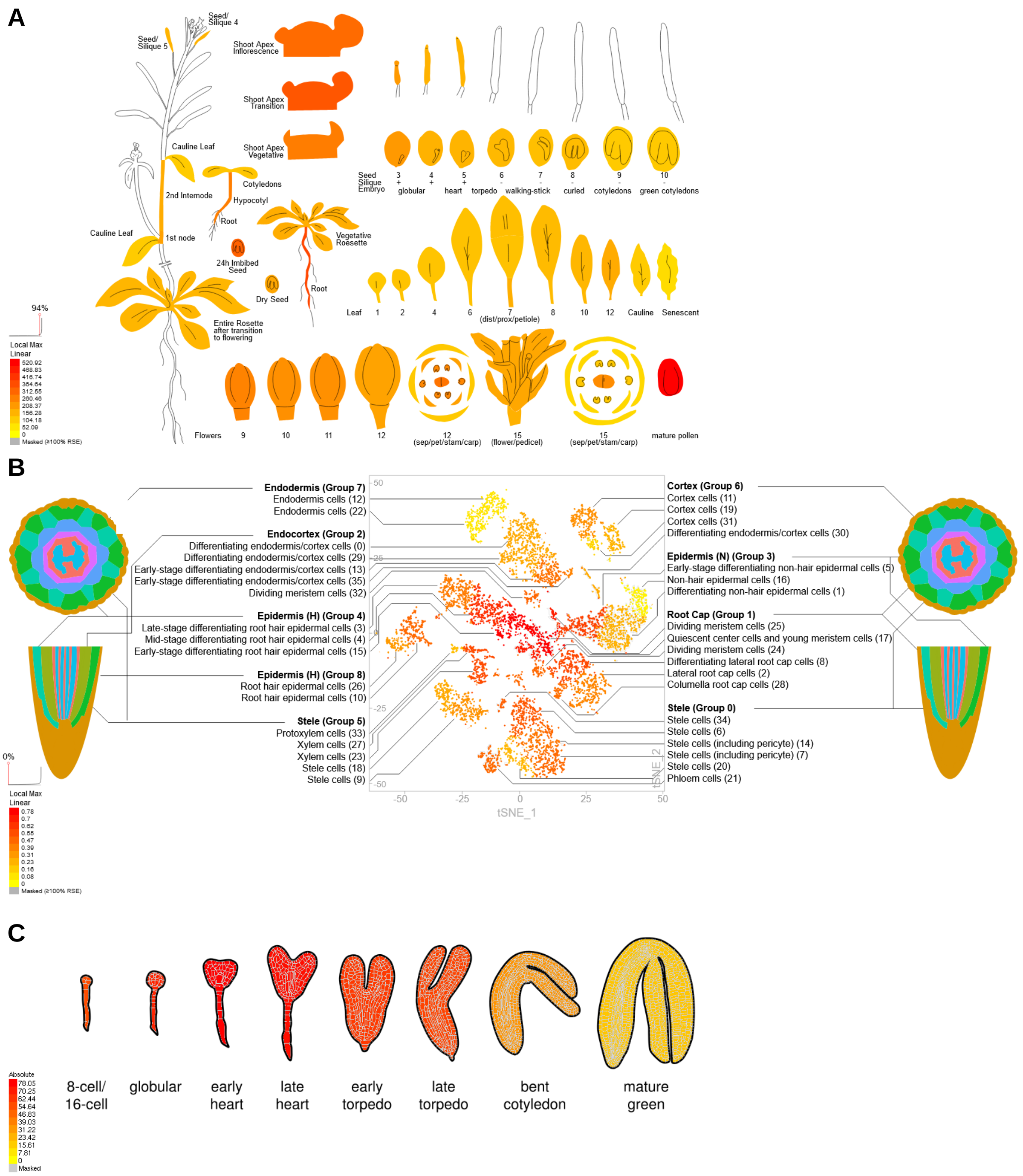

Fig. S1. Visualization of *TTN5* gene expression levels during plant development based on transcriptome data. Expression levels in (A) different types of aerial organs at different developmental stages; from left to right and bottom to top are represented different seed and plant growth stages, flower development stages, different leaves, vegetative to inflorescence shoot apex, embryo and silique development stages; (B) seedling root tissues based on single cell analysis represented in form of a uniform manifold approximation and projection plot; (C) successive stages of embryo development. As shown in (A) to (C), *TTN5* is ubiquitously expressed in these different plant organs and tissues. In particular, it should be noted that *TTN5* transcripts were detectable in the epidermis cell layer of roots that we used for

Journal of Cell Science • Supplementary information

localization of tagged TTN5 protein in this study. In accordance with the embryo-lethal phenotype, the ubiquitous expression of *TTN5* highlights its importance for plant growth. Original data were derived from (Nakabayashi et al. 2005, Schmid et al. 2005) (A); (Ryu et al. 2019) (B); (Waese et al. 2017) (C). Gene expression levels are indicated by local maximum color code, ranging from the minimum (no expression) in yellow to the maximum (highest expression) in red.

Journal of Cell Science • Supplementary information

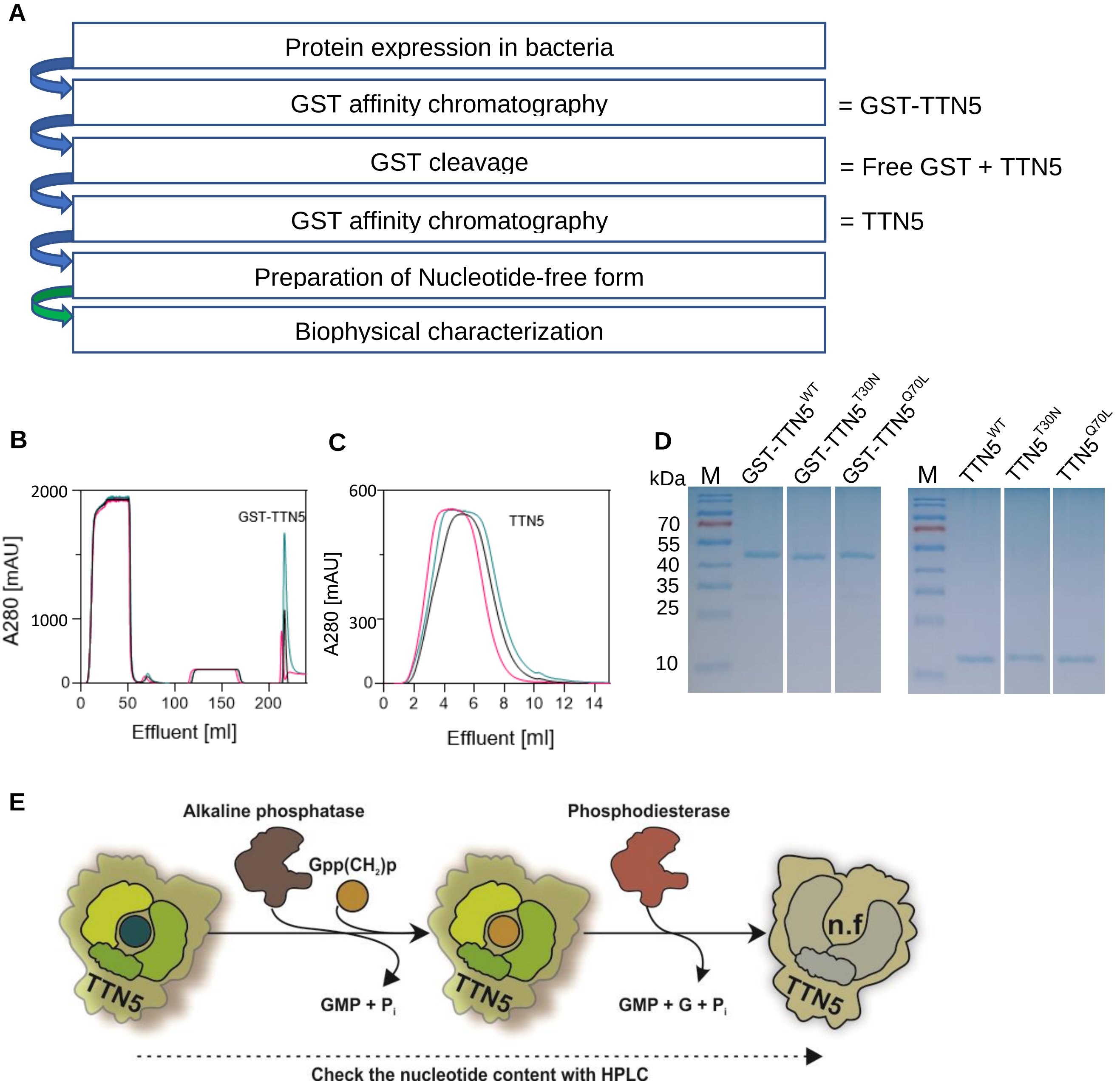
A
Protein expression in bacteria
GST affinity chromatography
= GST-TTN5
GST cleavage
= Free GST + TTN5
GST affinity chromatography
= TTN5
Preparation of Nucleotide-free form
Biophysical characterization
B
A280 [mAU]
GST-TTN5
Effluent [ml]
C
A280 [mAU]
TTN5
Effluent [ml]
D
kDa
M
GST-TTN5WT
GST-TTN5T30N
GST-TTN5Q70L
TTN5WT
TTN5T30N
TTN5Q70L
70
55
40
35
25
10
E
Alkaline phosphatase
Gpp(CH2)p
Phosphodiesterase
GMP + Pi
GMP + G + Pi
TTN5
n.f
Check the nucleotide content with HPLC

Fig. S2. Heterologous expression and purification of TTN5 protein as well as preparation of its nucleotide-free form. (A) Overview of protein purification and preparation of nucleotide-free form of TTN5 in the presence of excess GDP. (B-C) The chromatograms represent the GST-affinity chromatography to obtain GST-TTN5 and TTN5 variants, before and after GST-cleavage by thrombin. (D) Coomassie Blue SDS-PAGE of GST-TTN5 (left panel; 46.5 kDa) and TTN5 after GST-cleavage by thrombin and a second GST-affinity chromatography (right panel; 21 kDa). (E) Schematic illustration of nucleotide-free TTN5 preparation. In the first step, the purified GDP-bound TTN5 is incubated with alkaline phosphatase in the presence of 1.5-fold molar excess of Gpp(CH_2)p, which is a non-hydrolyzable GTP analog and, unlike GDP, resistant to alkaline phosphatase. After GDP is completely degraded, phosphodiesterase is added to the reaction to degrade Gpp(CH_2)p to GMP, G and Pi. The nucleotide content is monitored in each step by HPLC. The solution with nucleotide-free TTN5 is deep-frozen and thawed twice, aliquoted and stored at -80°C.

Journal of Cell Science • Supplementary information

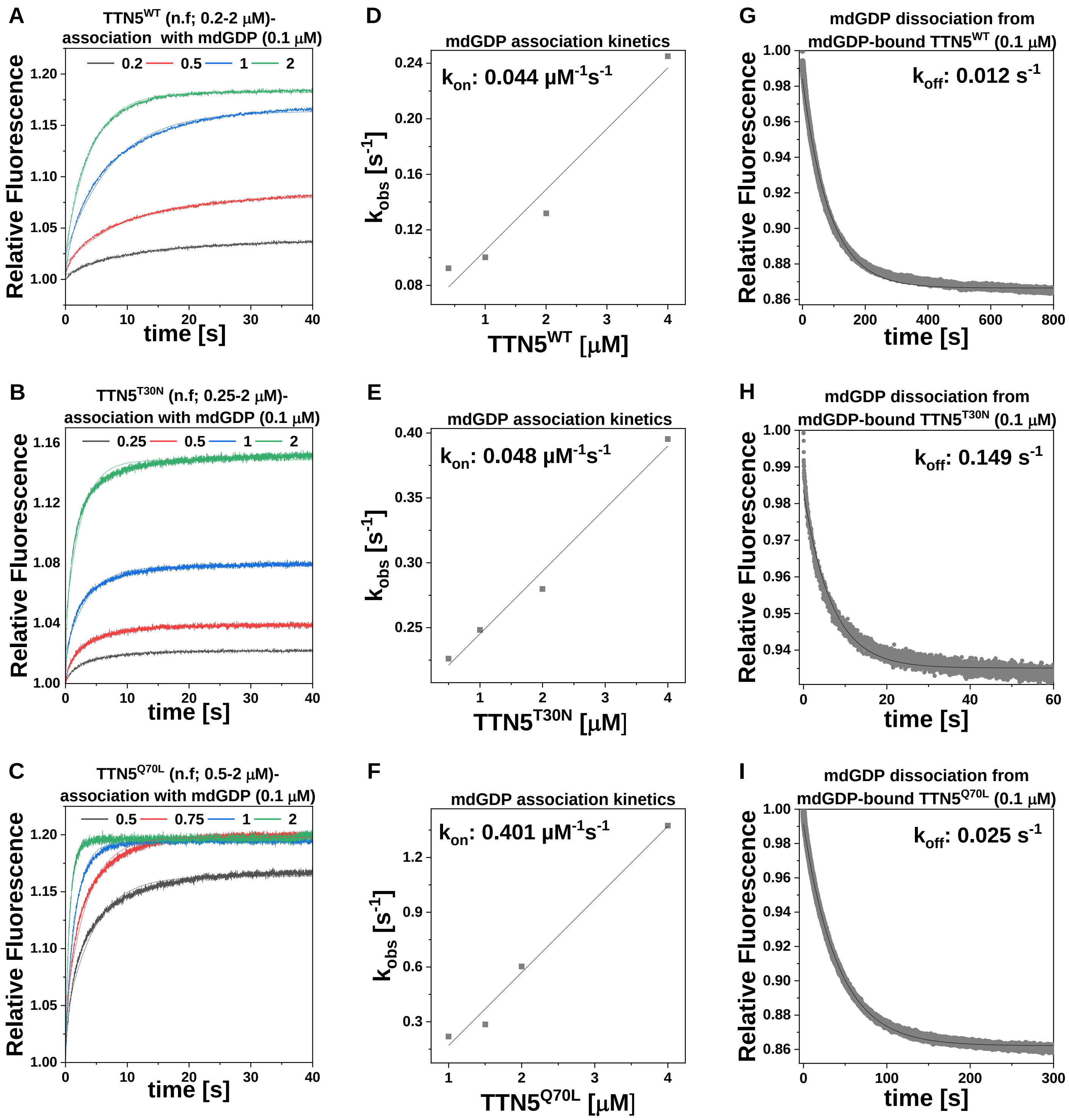

Journal of Cell Science • Supplementary information

Fig. S3. Kinetic measurements of mdGDP interaction with TTN5 proteins. (A-C) Association of mdGDP (0.1 µM) with increasing concentrations of $TTN5^{WT}$ (A) $TTN5^{T30N}$ (B) and $TTN5^{Q70L}$ (C). (D-F) Association rate constant (k_{on}) is determined by plotting the k_{obs} values, obtained from the exponential fits of the mdGDP association data (A-C) against the TTN5 (D), $TTN5^{T30N}$ (E) and $TTN5^{Q70L}$ (F) protein concentrations. (G-I), Dissociation of mdGDP from TTN5 (G), $TTN5^{T30N}$ (H) and $TTN5^{Q70L}$ (I) proteins (0.1 µM) is determined in the presence of excess amounts of unlabeled GDP (20 µM). The dissociation rates (k_{off}) are obtained from the exponential fitting of the data by Origin software. All results are shown as bar charts in Figure 2D. The principles of the assays are illustrated in Figure 2A-C.

Journal of Cell Science • Supplementary information

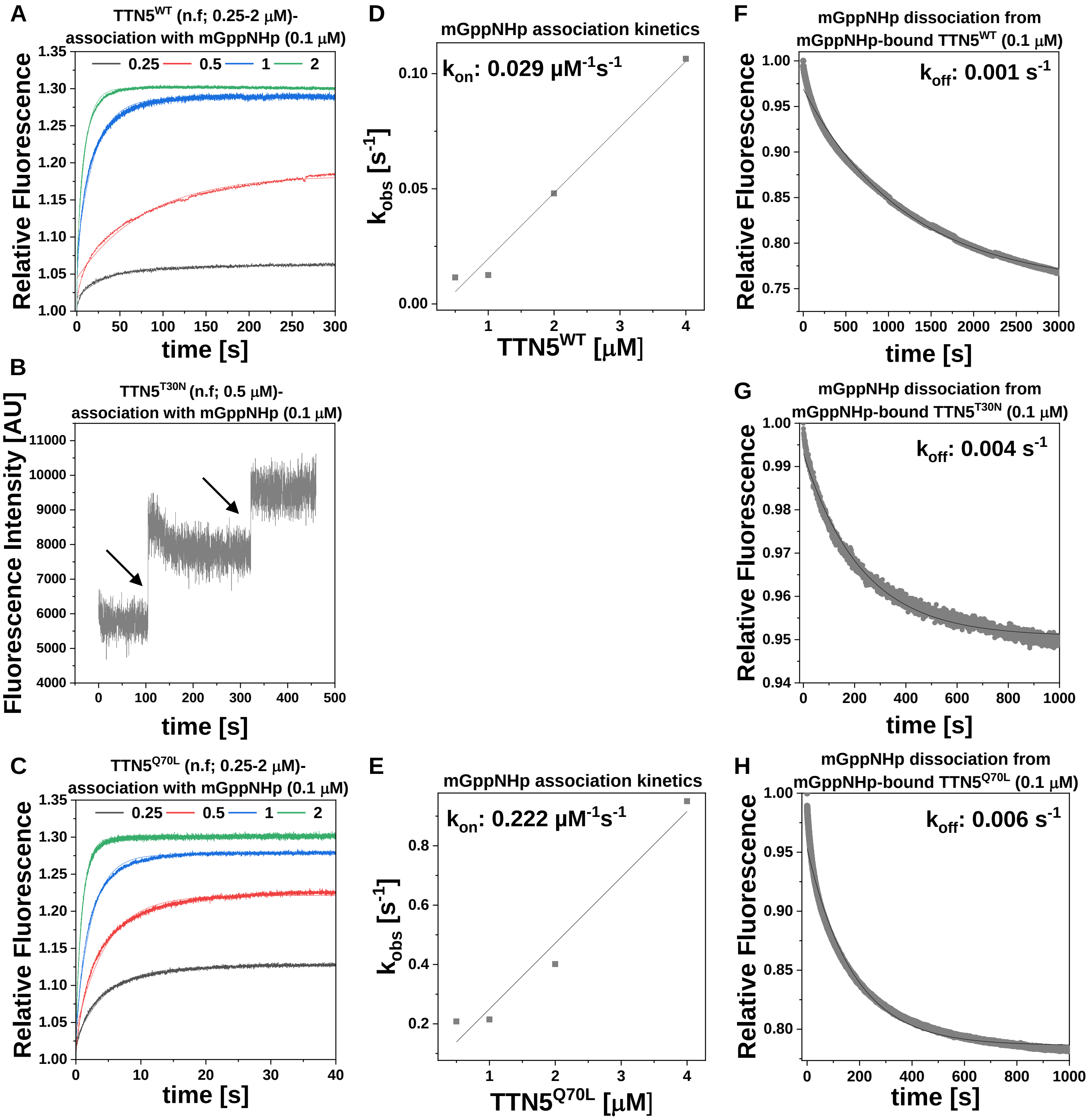

Journal of Cell Science • Supplementary information

Fig. S4. Kinetic measurements of mGppNHp interaction with TTN5 proteins. (A-E) Association kinetics. (A, C) Association of mGppNHp (0.1 µM) with increasing concentrations of TTN5^{WT} (A) and TTN5^{Q70L} (C). (B) When mixing mGppNHp with nucleotide-free TTN5^{T30N}, no binding was observed under these experimental conditions. Instead, association of mGppNHp (0.1 µM) with two different titrations of 0.5 µM TTN5^{T30N} was measured, indicated by arrows, to confirm the fast binding of TTN5^{T30N} with mGppNHp by increasing fluorescence intensity, determined by fluorimeter. (D-E) Association rate constant (k_{on}) is determined by plotting the k_{obs} values, obtained from the exponential fits of the mGppNHp association data (left panels) against the TTN5^{WT} (D) and TTN5^{Q70L} (E) protein concentrations. Note that TTN5^{T30N} did not show association with mGppNHp, therefore middle space is left empty. (F-H) Dissociation of mGppNHp from TTN5^{WT} (F), TTN5^{T30N} (G) and TTN5^{Q70L} (H) proteins (0.1 µM) is determined in the presence of excess amounts of unlabeled GppNHp (20 µM). The dissociation rates (k_{off}) are obtained from the exponential fitting of the data by Origin software. All results are shown as bar charts in Figure 2E. The principles of the assays are illustrated in Figure 2A-C.

Journal of Cell Science • Supplementary information

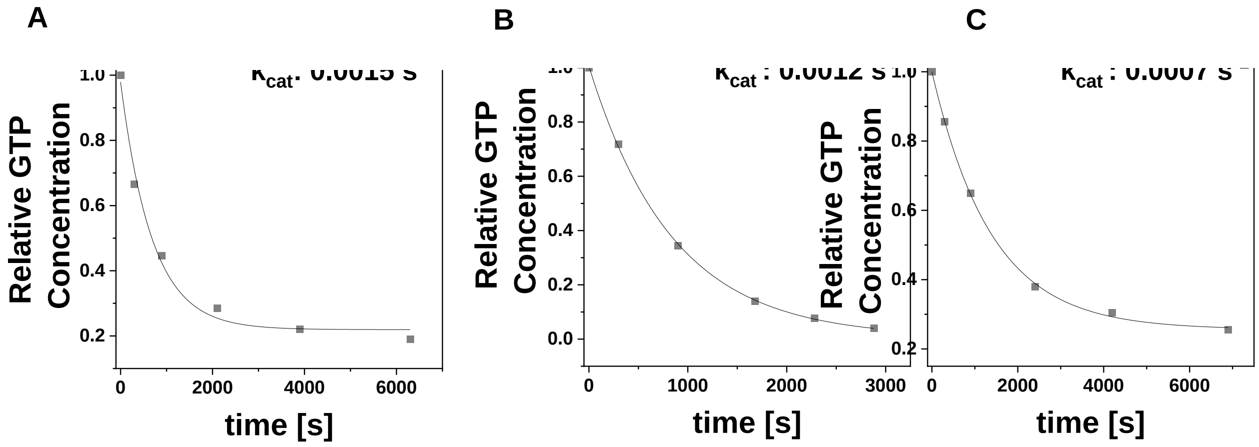

Fig. S5. GTP hydrolysis reaction rates of the TTN5 proteins. (A-C) The GTP hydrolysis reaction rates (k_{cat}) of TTN5^{WT} (A), TTN5^{T30N} (B) and TTN5^{Q70L} (C) proteins (100 µM) were measured at 25°C. Aliquots of the reaction mixture at the indicated time points were analyzed by HPLC as described in Materials and Methods. The obtained results are shown as bar charts in Figure 2G. The principle of the assay is illustrated in Figure 2F.

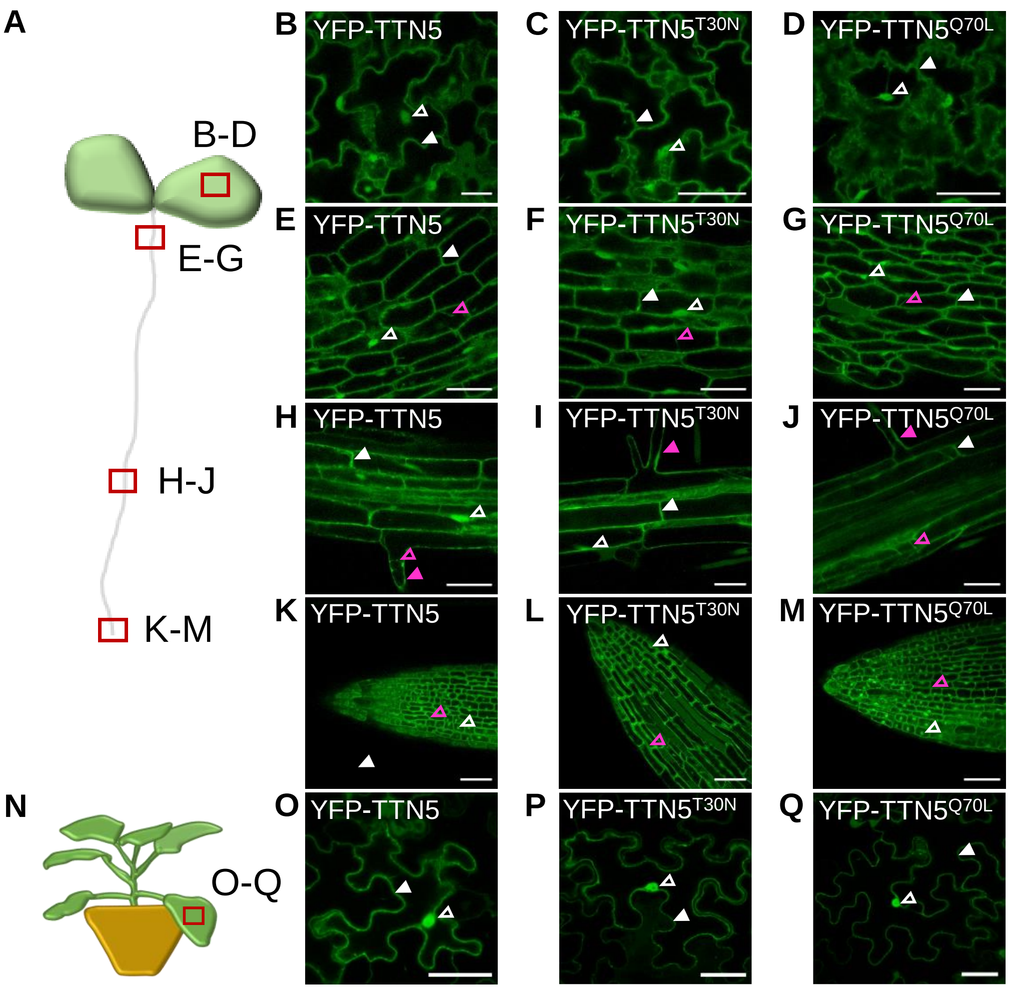

Fig. S6. YFP fluorescence signal localization in YFP-TTN5 and -TTN5 mutant Arabidopsis seedlings. Microscopic YFP fluorescence observations were made in a plane across the centers of most cells. (A) Schematic representation of an Arabidopsis seedling. Images were taken at four different positions of the seedlings and imaged areas are indicated by a red rectangle. (B-M) Fluorescent YFP signals in Arabidopsis seedlings *via* fluorescent confocal microscopy. (B–D) YFP signal localization was observed in the epidermis of cotyledons. Fluorescence signals were present in nucleus (indicated by empty white arrowhead) and cytoplasm and at or in close proximity to the plasma membrane (indicated by filled white arrowhead) in the epidermis of cotyledons of YFP-TTN5, YFP-TTN5^{T30N} and YFP-TTN5^{Q70L} seedlings. (E-G) Fluorescence localization in the hypocotyls showed clear presence in the cytoplasm (indicated by empty magenta arrowhead), next to nuclear and plasma membrane-related localization. (H-J) Fluorescence signals were present in nuclei, cytoplasm, at or close to the plasma membrane in the root hair zone and in root hairs (indicated by filled magenta arrowhead) with clear cytoplasmic localization of YFP-TTN5, YFP-TTN5^{T30N} and YFP-TTN5^{Q70L} seedlings. (K-M) Fluorescence signal localization at the root tip showed clear expression in nuclei and cytoplasm, visible due to the smaller size of the vacuoles. (N)

Journal of Cell Science • Supplementary information

Schematic representation of a *N. benthamiana* plant, used for leaf infiltration for transient expression of YFP-TTN5, YFP-TTN5^{T30N} and YFP-TTN5^{Q70L}. Imaged areas are indicated by a red rectangle. (O-Q) Fluorescent signals in *N. benthamiana* leaf epidermal cells *via* fluorescent confocal microscopy. Signals were present in nucleus and cytoplasm and at or in close proximity to the plasma membrane. Experiment with Arabidopsis lines was done once experiments in *N. benthamiana* were repeated three times. Scale bar 50 µm.

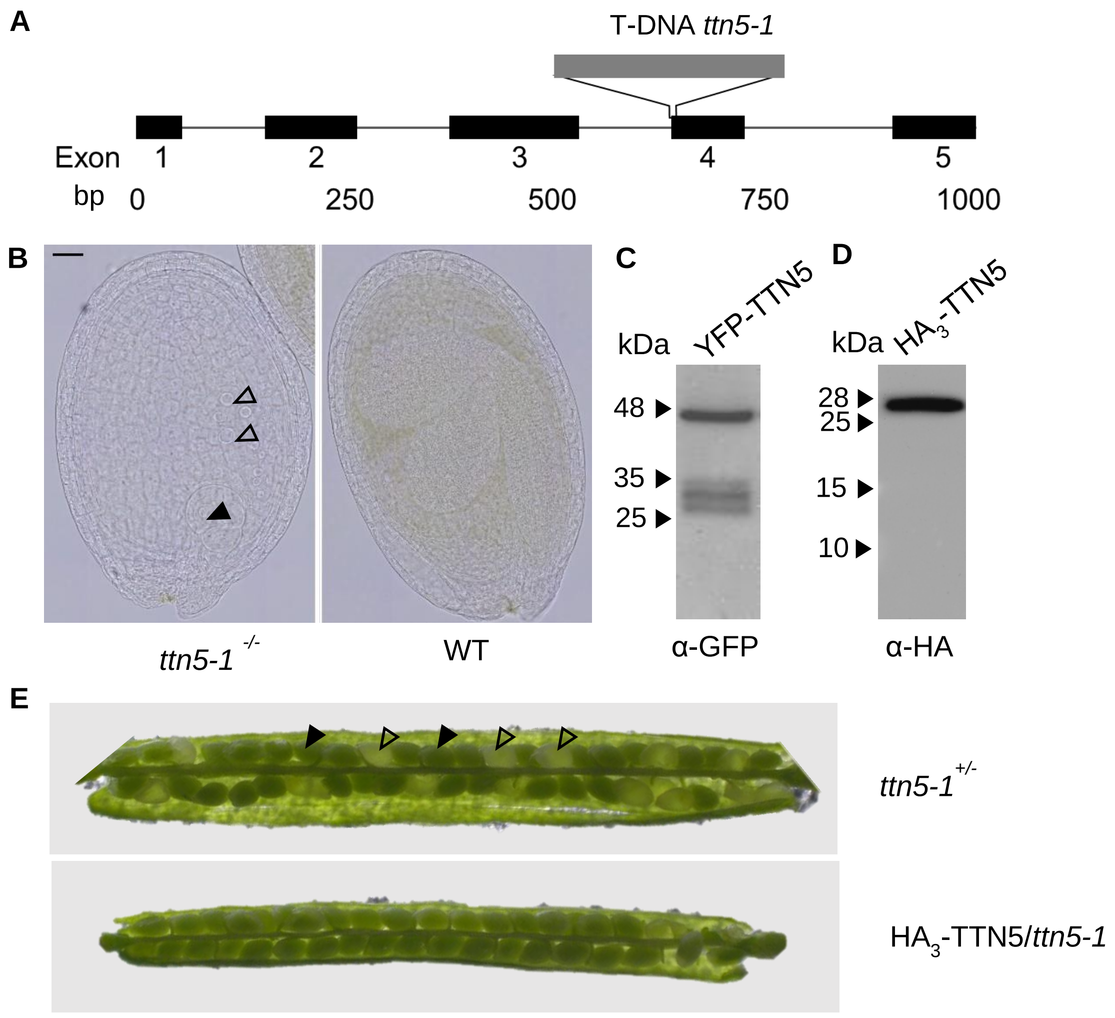

Fig. S7. Phenotypes of *ttn5-1* T-DNA insertion line and root length of HA_3-TTN5 lines. (A) Schematic representation of the *TTN5* exon-intron structure and T-DNA insertion in the *ttn5-1* allele. The numbers below the exon numbers indicate base pairs (bp). (B) Early embryo arrest phenotype of a homozygous *ttn5-1*$^{-/-}$ seed on the left in comparison with a wild type (WT) seed of the same silique on the right; arrowheads, indicate enlarged nuclei in the arrested embryo (filled) and endosperm (empty). Embryo-lethal phenotype was imaged in five seeds (n = 5). Early embryo arrest phenotypes were previously shown by (Mayer et al.

1999, McElver et al. 2000). Scale bar 1 cm. (C) Western blot control of YFP-TTN5 protein expressed in stable transgenic plant lines. α-GFP primary plus secondary antibody treatment detected a strong band at the correct size of 48 kDa for YFP-TTN5 and three additional smaller sized weak bands 26-35 kDa. Plants were grown for 6 days. (D) Western blot control of HA_3-TTN5 protein expressed in stable transgenic plant lines. One single band was detected by α-HA-HRP antibody treatment at the correct size of 28 kDa for HA_3-TTN5 protein. Plants were grown in the two-week system. Western blots were repeated three times. (E) Siliques of heterozygous *ttn5-1*$^{+/-}$ plants, containing ca. 25 % white seeds with arrested embryos (empty arrowheads) and ca. 75 % regular green seeds (filled arrowheads). HA_3-TTN5 line crossed to *ttn5-1*$^{+/-}$ was able to rescue embryo lethal seed phenotype. Phenotype was checked in seven siliques (n = 7).

Journal of Cell Science • Supplementary information

A

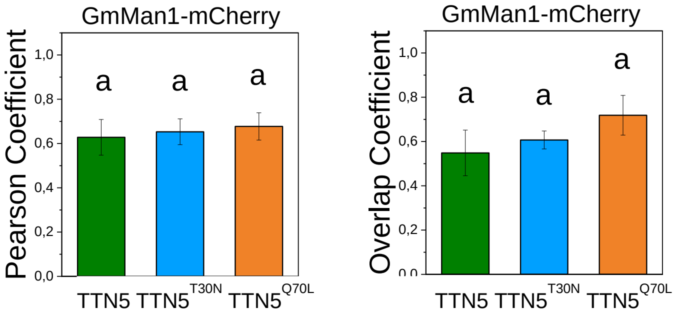

B

Channel		Colocalization	
YFP	mCherry	YFP/mCh	mCh/YFP
YFP-TTN5	GmMan1-mCherry	23.85±22.77	42.62±21.85
YFP-TTN5^{T30N}	GmMan1-mCherry	15.83±10.21	31.25±14.88
YFP-TTN5^{Q70L}	GmMan1-mCherry	14.81±6.79	47.82±35.04

C

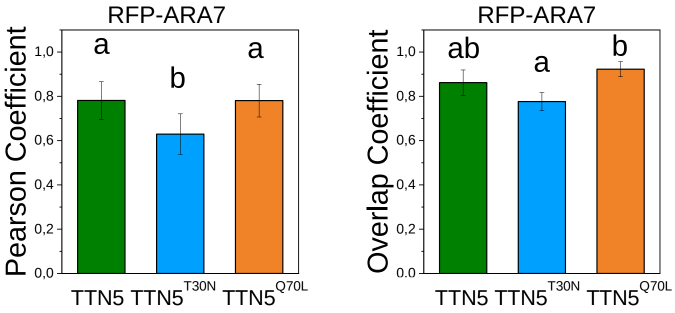

D

Channel		Colocalization	
YFP	mRFP	YFP/mRFP	mRFP/YFP
YFP-TTN5	mRFP-ARA7	58.75±15.69	28.94±11.96
YFP-TTN5^{T30N}	mRFP-ARA7	51.79±8.12	21.21±11.76
YFP-TTN5^{Q70L}	mRFP-ARA7	64.90±6.15	75.05±10.66

Journal of Cell Science • Supplementary information

Fig. S8. Colocalization analysis of YFP fluorescent signal with GmMan1-mCherry and mRFP-ARA7 signals in YFP-TTN5, YFP-TTN5^{T30N} and YFP-TTN5^{Q70L} seedlings. Colocalization analyses were conducted with specific markers using ImageJ (Schneider et al. 2012). (A) JACoP-based colocalization analysis (Bolte and Cordelières 2006). Comparison of Pearson's and Overlap coefficients for *cis*-Golgi-located GmMan1-mCherry with the YFP fluorescence signals of three YFP-TTN5 seedlings in vesicle-like structures. Fluorescent signals colocalized similarly with the *cis*-Golgi marker in YFP-TTN5, YFP-TTN5^{T30N} and YFP-TTN5^{Q70L} seedlings. (B) Object-based analysis was performed for vesicle-like structures based on distance between geometrical centers of signals. YFP fluorescent signal-positive structures overlapped most with GmMan1-mCherry-positive Golgi stacks in YFP-TTN5 compared to YFP-TTN5^{T30N} and YFP-TTN5^{Q70L} seedlings. GmMan1-mCherry-positive Golgi stacks overlapped most with YFP-TTN5^{Q70L}-positive structures followed by YFP-TTN5 and YFP-TTN5^{T30N} signals. (C) JACoP-based colocalization analysis (Bolte and Cordelières 2006). Comparison of Pearson's and Overlap coefficients for TGN/MVB-located mRFP-ARA7 with YFP fluorescence signals of YFP-TTN5, YFP-TTN5^{T30N} and YFP-TTN5^{Q70L} seedlings in vesicle-like structures. YFP fluorescence signals in YFP-TTN5 and YFP-TTN5^{Q70L} seedlings colocalized similarly with mRFP-ARA7, whereas YFP signals tended to colocalize less in YFP-TTN5^{T30N} seedlings. (D) Object-based analysis was performed for vesicle-like structures based on distance between geometrical centers of signals. More than half of signals corresponding to all YFP-TTN5-positive structures overlapped with mRFP-ARA7-positive structures, in the order YFP-TTN5^{T30N}, YFP-TTN5 and best YFP-TTN5^{Q70L}. mRFP-ARA7-positive structures overlapped most with YFP fluorescence signals in YFP-TTN5^{Q70L} seedlings, while signals were reduced for YFP-TTN5 and YFP-TTN5^{T30N} seedlings by ca. 3.5-fold and 2.5-fold, respectively. Analyses were conducted in three replicates each (n = 3). One-way ANOVA with Fisher-LSD post-hoc test was performed. Different letters indicate statistical significance ($p < 0.05$).

Table S1. Primer list.

Available for download at
https://journals.biologists.com/jcs/article-lookup/doi/10.1242/jcs.262315#supplementary-data

Journal of Cell Science • Supplementary information

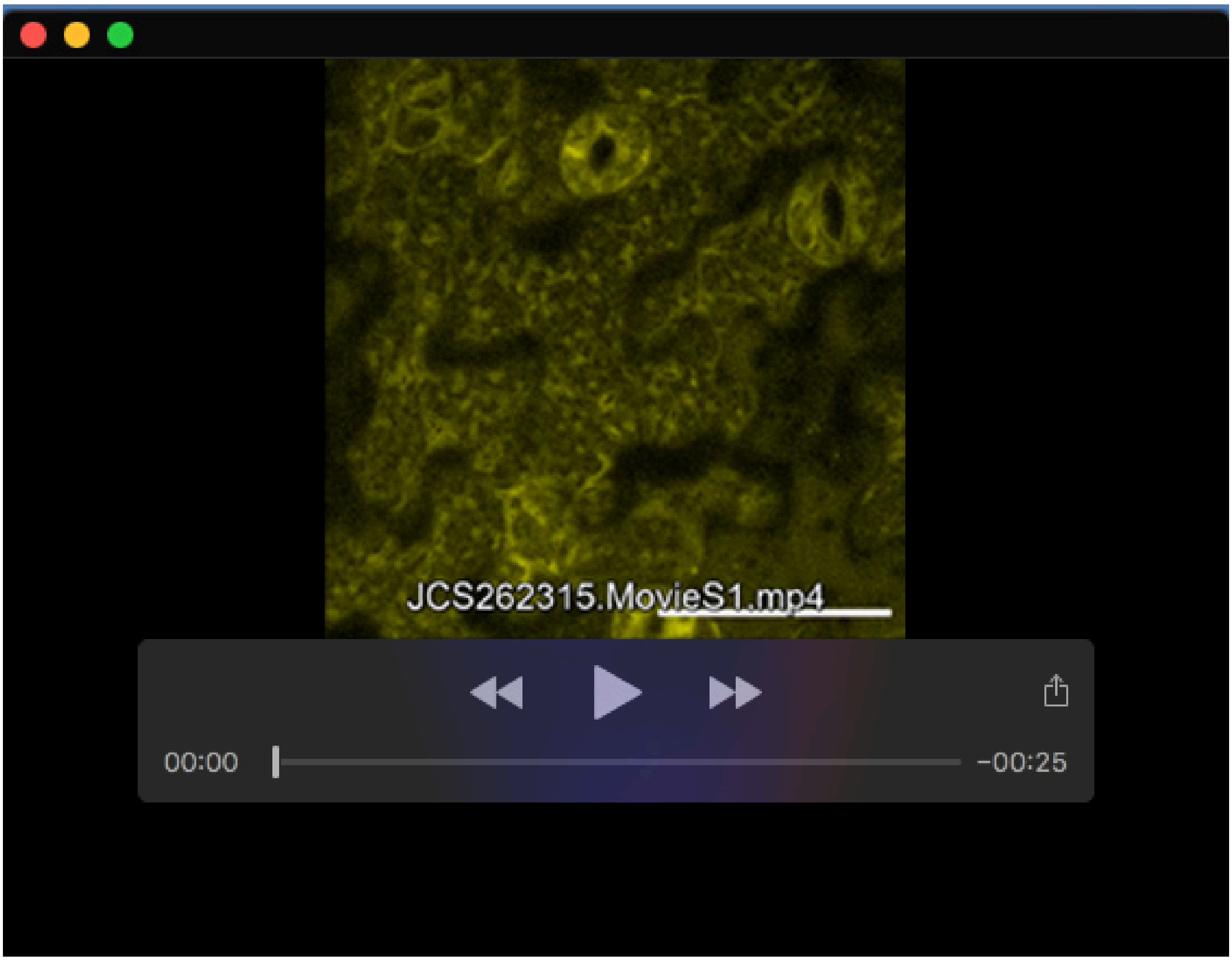

Movie 1. YFP fluorescence signals are present in dynamic vesicle-like structures in YFP-TTN5 seedlings in epidermal cells of cotyledons. Time series of YFP fluorescent signals in dynamic vesicle-like structures of YFP-TTN5 Arabidopsis seedlings recorded *via* fluorescence confocal microscopy. Fluorescence signals were present in dynamic vesicle-like structures in epidermal cotyledon cells and stomata. Experiment was performed once. Experiment was performed twice. Scale bar 50 μm.

Journal of Cell Science • Supplementary information

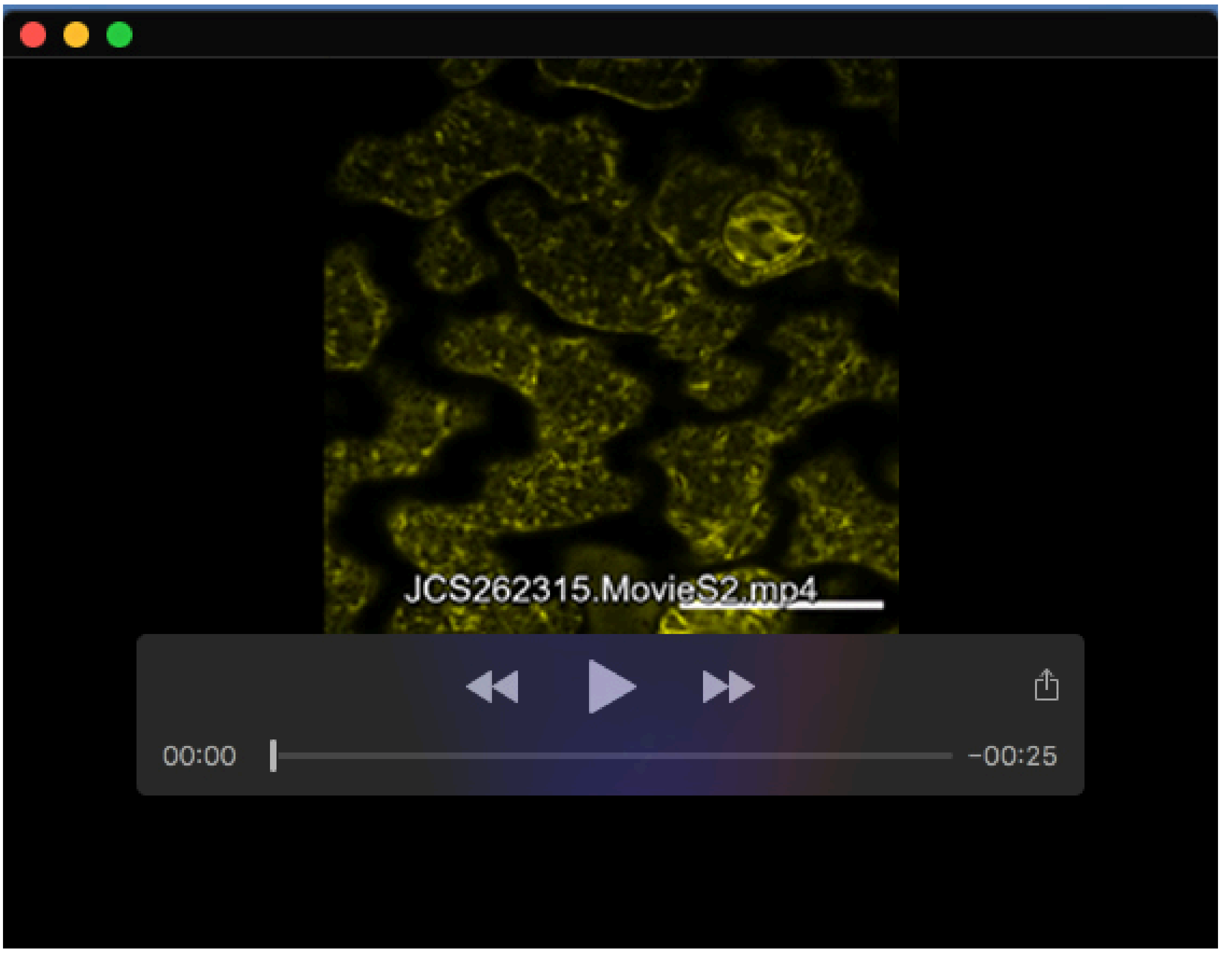

Movie 2. YFP fluorescence signals are present in dynamic vesicle-like structures in YFP-TTN5^{T30N} seedlings in epidermal cells of cotyledons. Time series of YFP fluorescent signals in dynamic vesicle-like structures of YFP-TTN5^{T30N} Arabidopsis seedlings recorded *via* fluorescence confocal microscopy. Fluorescence signals were present in dynamic vesicle-like structures in epidermal cotyledon cells and stomata. Experiment was performed once. Experiment was performed twice. Scale bar 50 µm.

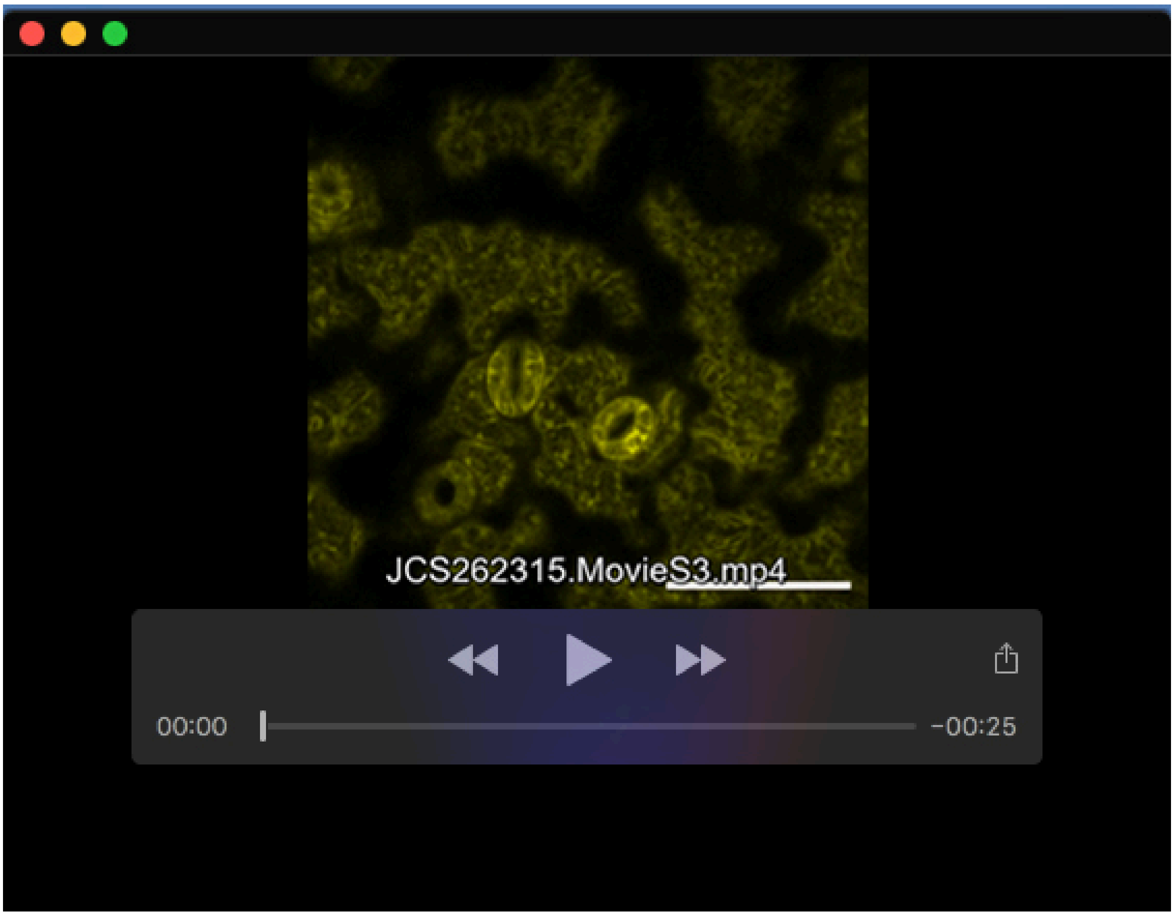

Movie 3. YFP fluorescence signals are present in dynamic vesicle-like structures in YFP-TTN5^{Q70L} seedlings in epidermal cells of cotyledons. Time series of YFP fluorescent signals in dynamic vesicle-like structures of YFP-TTN5^{Q70L} Arabidopsis seedlings recorded *via* fluorescence confocal microscopy. Fluorescence signals were present in dynamic vesicle-like structures in epidermal cotyledon cells and stomata. Experiment was performed once. Experiment was performed twice. Scale bar 50 µm.

Journal of Cell Science • Supplementary information

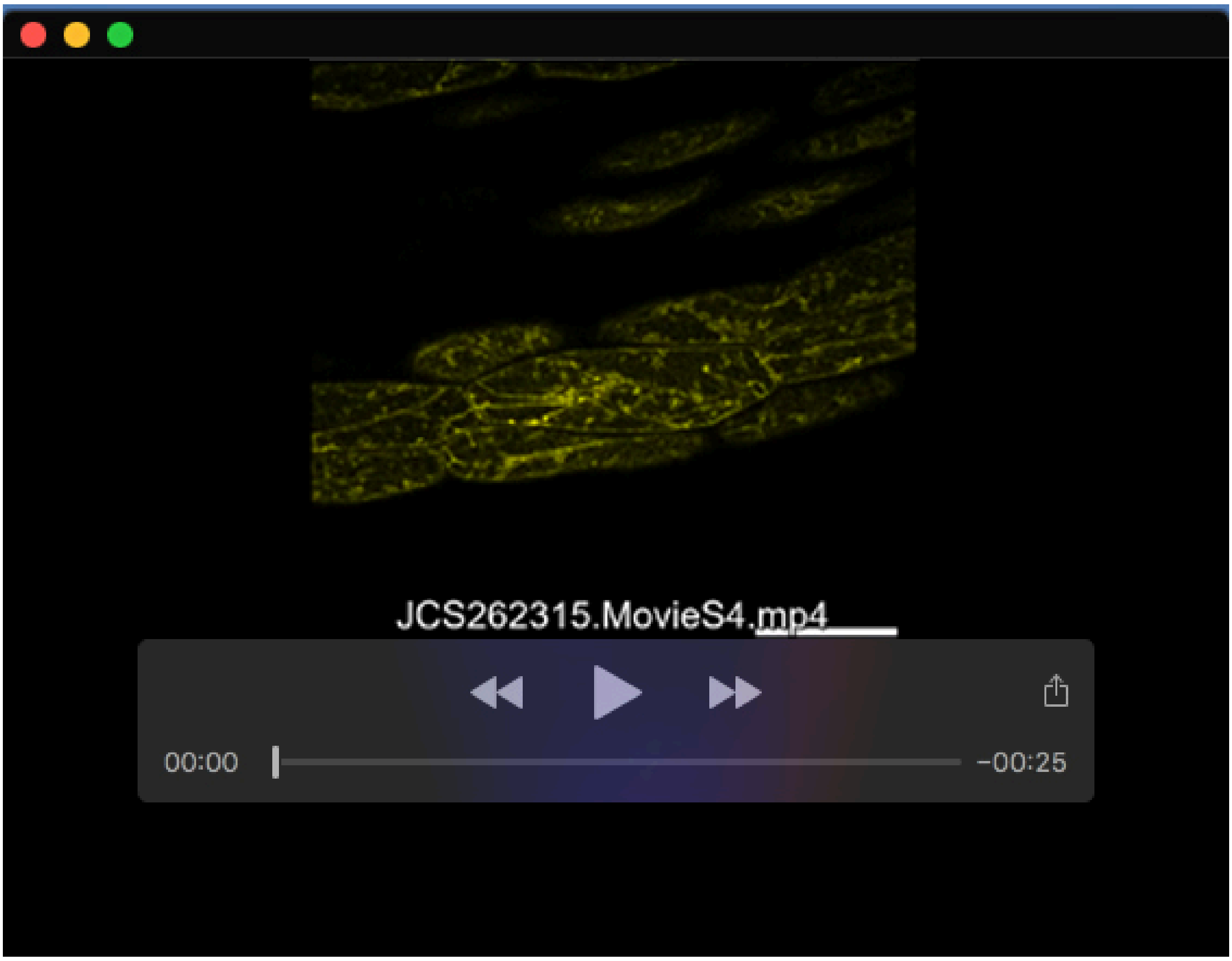

Movie 4. YFP fluorescence signals are present in dynamic vesicle-like structures in YFP-TTN5 seedlings in hypocotyls. Time series of YFP fluorescent signals in dynamic vesicle-like structures of YFP-TTN5 Arabidopsis seedlings recorded *via* fluorescence confocal microscopy. Fluorescence signals were present in dynamic vesicle-like structures in the hypocotyls. Experiment was performed once. Experiment was performed twice. Scale bar 50 µm.

Journal of Cell Science • Supplementary information

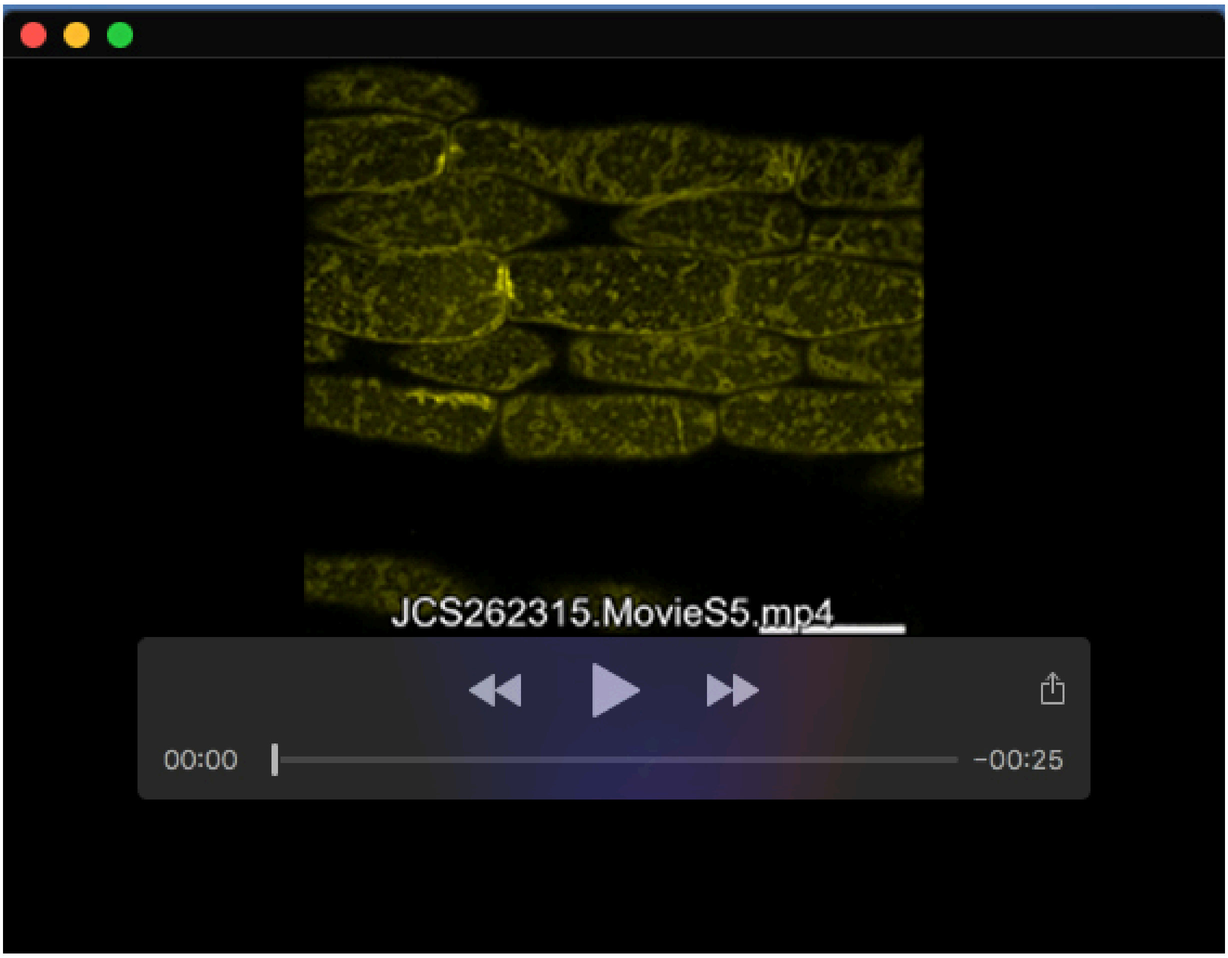

Movie 5. YFP fluorescence signals in YFP-TTN5^{T30N} seedlings in hypocotyls showed slowed down movement of dynamic vesicle-like structures. Time series of YFP fluorescent signals in dynamic vesicle-like structures of YFP-TTN5^{T30N} Arabidopsis seedlings recorded *via* fluorescence confocal microscopy. Fluorescence signals were present in dynamic vesicle-like structures in the hypocotyls. Note, that mobility of fluorescence signal of YFP-TTN5^{T30N} seedlings differed by a slower or aborted motion in half of the cells compared to YFP-TTN5 (Movie S4) and YFP-TTN5^{Q70L} (Movie S6). Experiment was performed once. Experiment was performed twice. Scale bar 50 µm.

Journal of Cell Science • Supplementary information

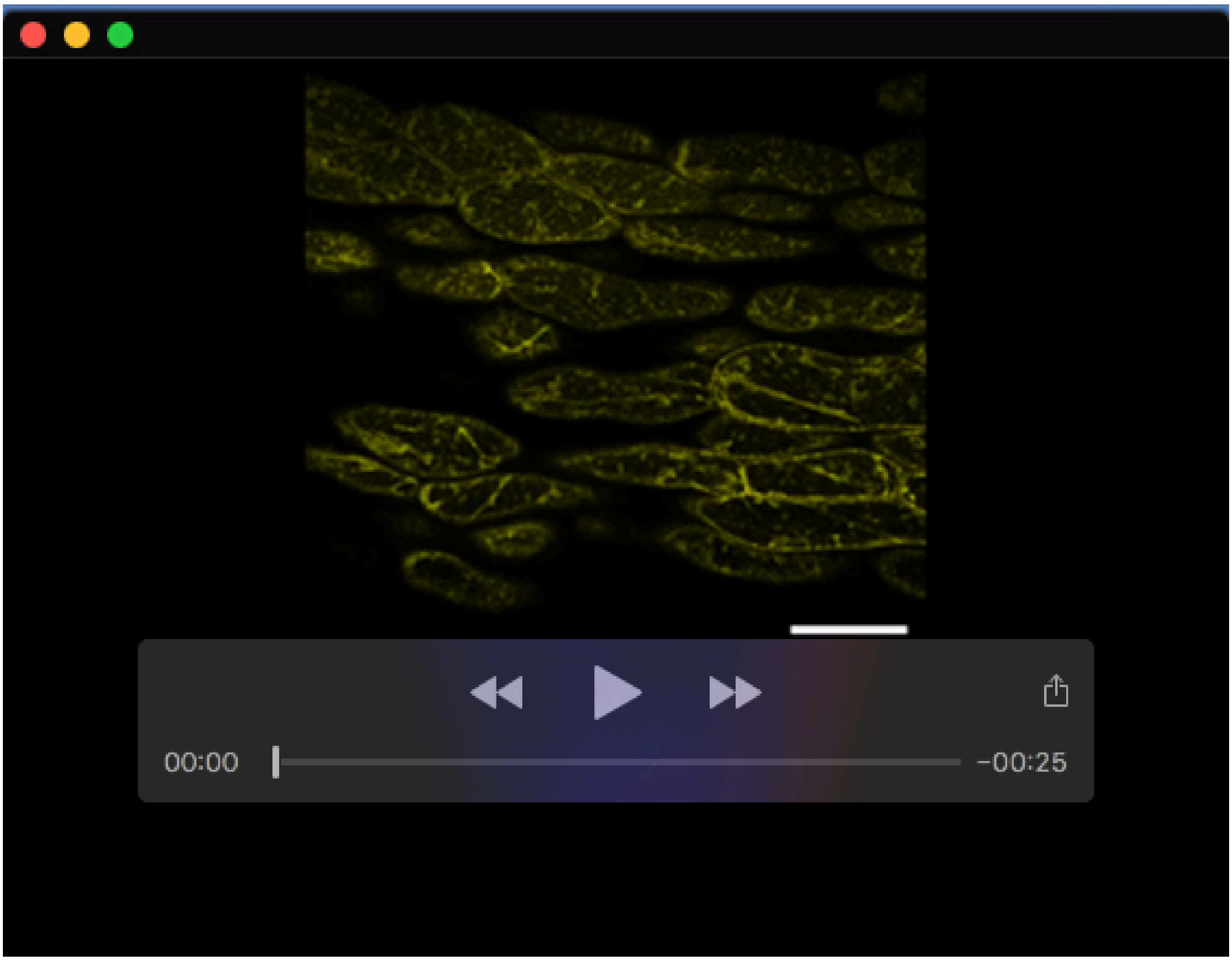

Movie 6. YFP fluorescence signals are present in dynamic vesicle-like structures in YFP-TTN5^{Q70L} seedlings in hypocotyls. Time series of YFP fluorescent signals in dynamic vesicle-like structures of YFP-TTN5^{Q70L} Arabidopsis seedlings (A-L) recorded *via* fluorescence confocal microscopy. Fluorescence signals were present in dynamic vesicle-like structures in the hypocotyls. Experiment was performed twice. Experiment was performed once. Scale bar 50 µm.

Journal of Cell Science • Supplementary information

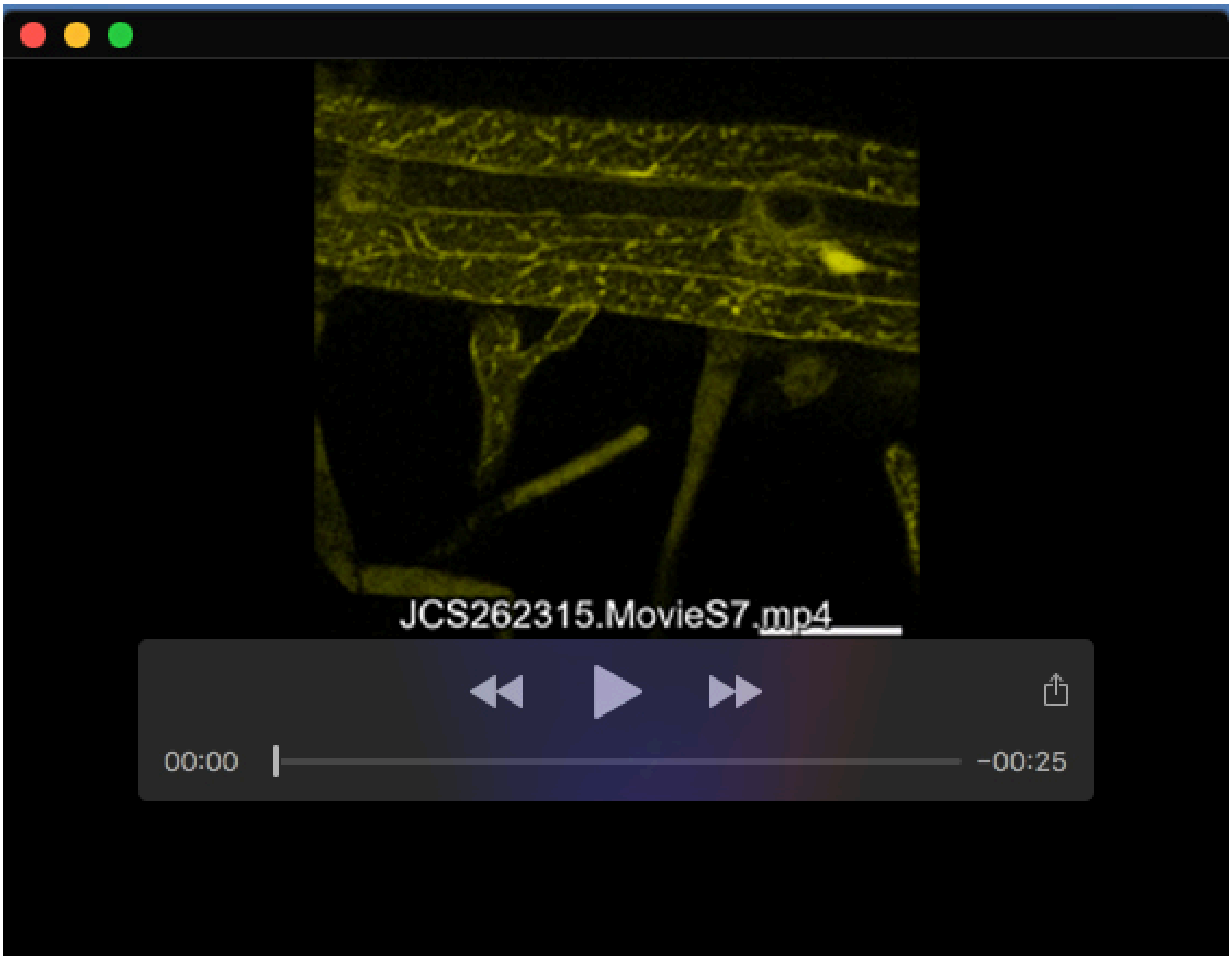

Movie 7. YFP fluorescence signals are present in dynamic vesicle-like structures in YFP-TTN5 seedlings in the root hair zone. Time series of YFP fluorescent signals in dynamic vesicle-like structures of YFP-TTN5 Arabidopsis seedlings recorded *via* fluorescence confocal microscopy. YFP fluorescence signals were present in the dynamic vesicle-like structures in the root hair zone and in root hairs as well. Experiment was performed twice. Scale bar 50 µm.

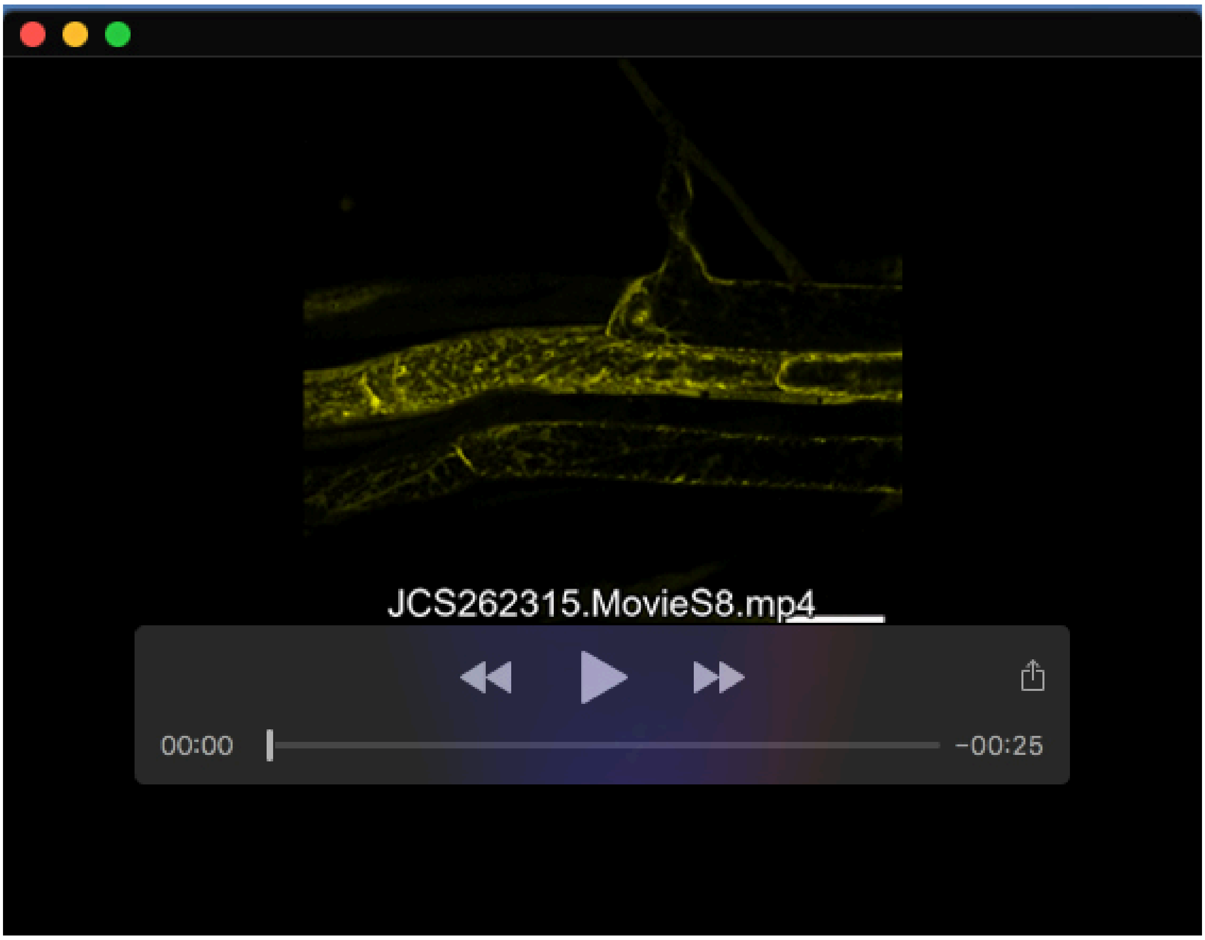

Movie 8. YFP fluorescence signals are present in dynamic vesicle-like structures in YFP-TTN5^{T30N} seedlings in the root hair zone. Time series of YFP fluorescent signals in dynamic vesicle-like structures of YFP-TTN5^{T30N} Arabidopsis seedlings recorded *via* fluorescence confocal microscopy. YFP fluorescence signals were present in the dynamic vesicle-like structures in the root hair zone and in root hairs as well. Experiment was performed once. Experiment was performed twice. Scale bar 50 µm.

Journal of Cell Science • Supplementary information

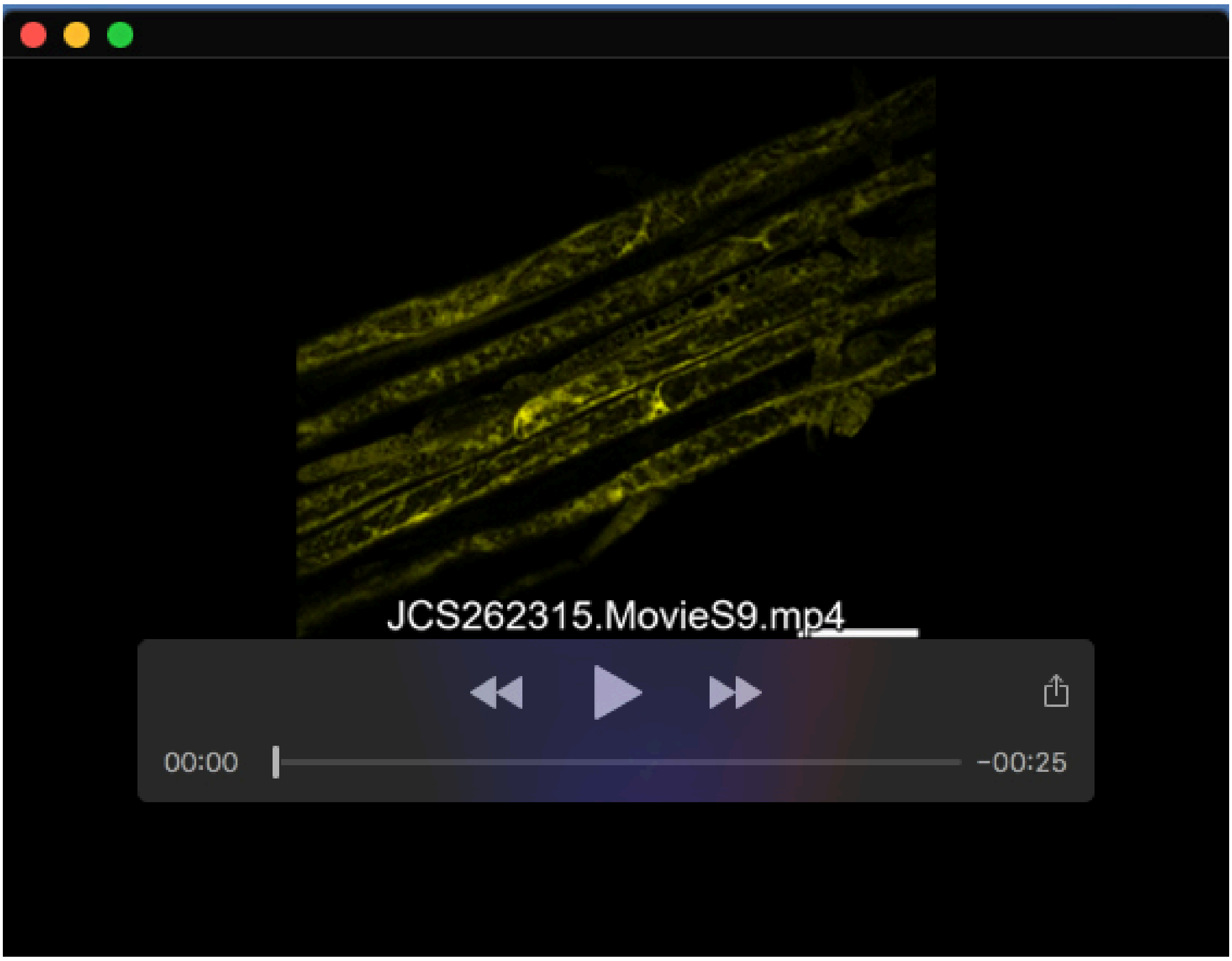

Movie 9. YFP fluorescence signals are present in dynamic vesicle-like structures in YFP-TTN5^{Q70L} seedlings in the root hair zone. Time series of YFP fluorescent signals in dynamic vesicle-like structures of YFP-TTN5^{Q70L} Arabidopsis seedlings recorded *via* fluorescence confocal microscopy. YFP fluorescence signals were present in the dynamic vesicle-like structures in the root hair zone and in root hairs as well. Experiment was performed once. Experiment was performed twice. Scale bar 50 µm.

Journal of Cell Science • Supplementary information

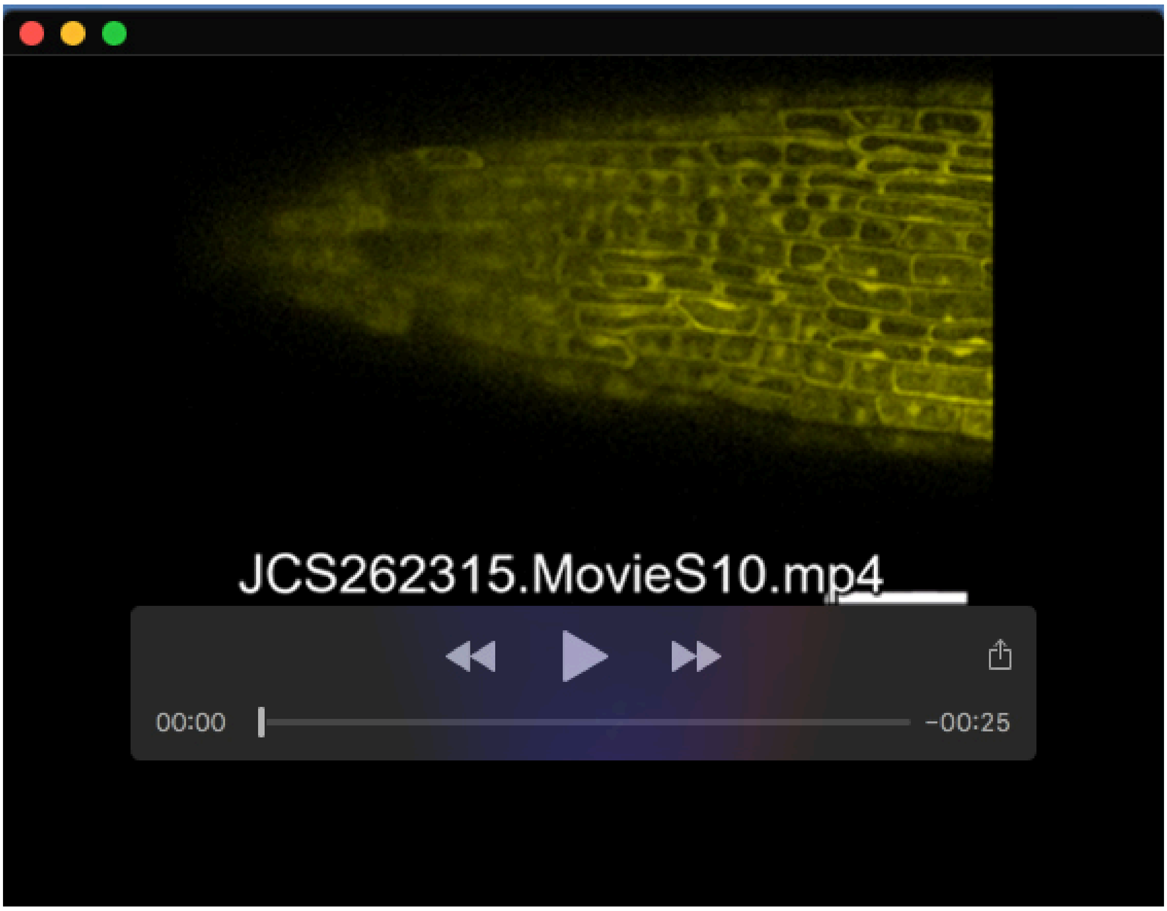

Movie 10. YFP fluorescence signals are present in dynamic vesicle-like structures in YFP-TTN5 seedlings in the root tip. Time series of YFP fluorescent signals in dynamic vesicle-like structures of YFP-TTN5 Arabidopsis seedlings recorded *via* fluorescence confocal microscopy. Fluorescence signals were present in cells of the root tip but without clear mobility or vesicle-like structures as in the cotyledons, hypocotyls and root hair zone. Experiment was performed once. Scale bar 50 µm.

Journal of Cell Science • Supplementary information

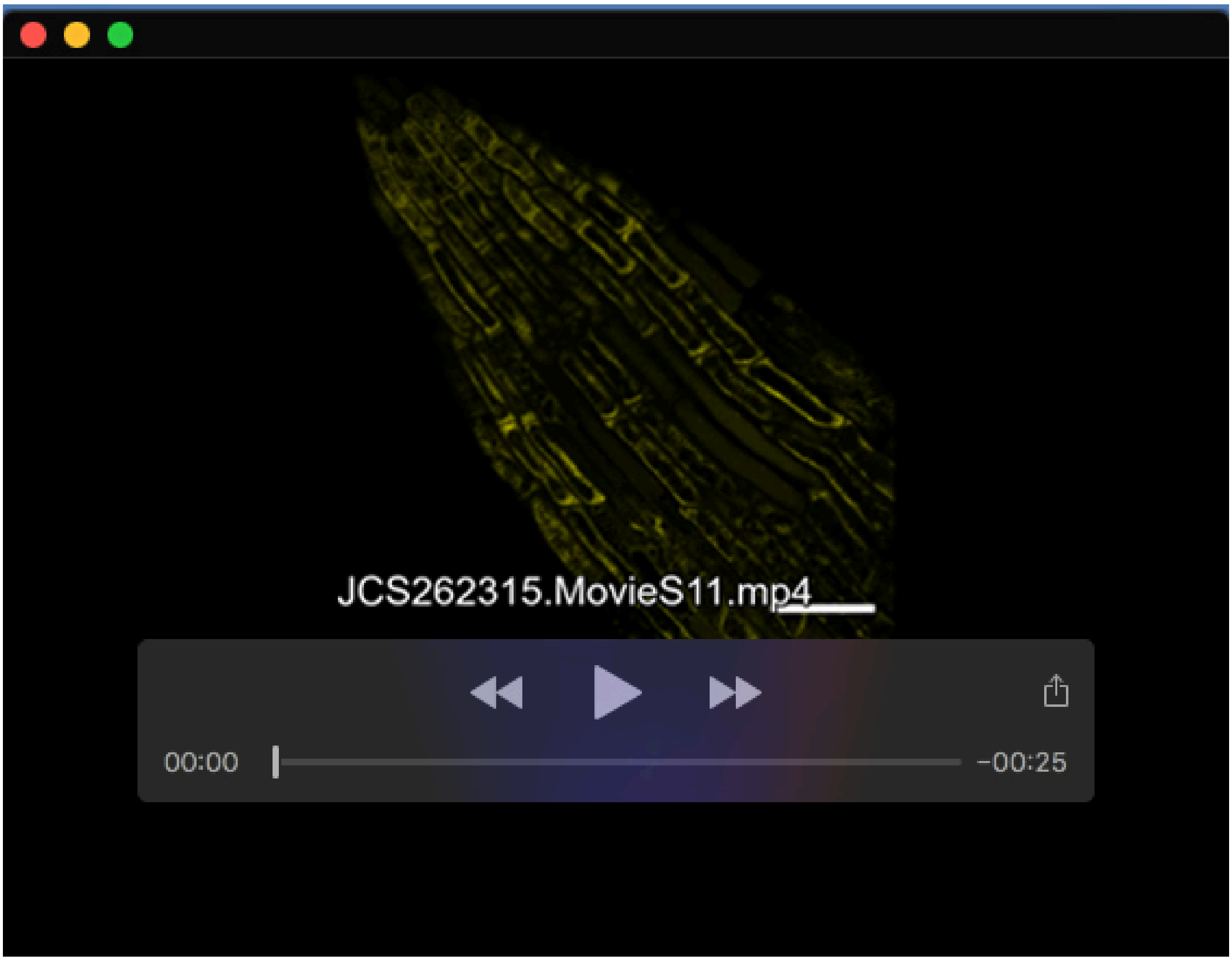

Movie 11. YFP fluorescence signals are present in dynamic vesicle-like structures in YFP-TTN5^{T30N} seedlings in the root tip. Time series of YFP fluorescent signals in dynamic vesicle-like structures of YFP-TTN5^{T30N} Arabidopsis seedlings recorded *via* fluorescence confocal microscopy. Fluorescence signals were present in cells of the root tip but without clear mobility or vesicle-like structures as in the cotyledons, hypocotyls and root hair zone. Experiment was performed once. Scale bar 50 µm.

Journal of Cell Science • Supplementary information

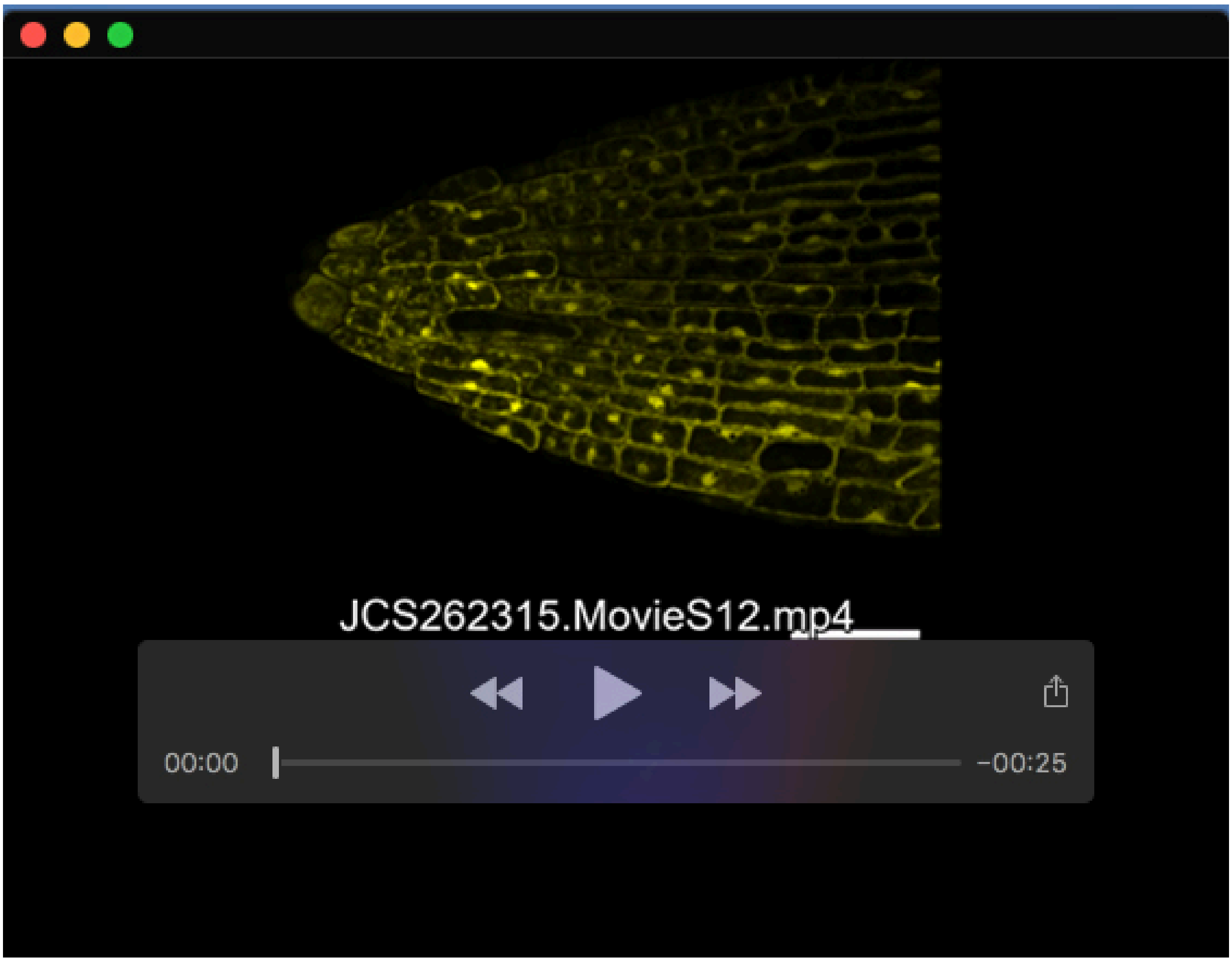

Movie 12. YFP fluorescence signals are present in dynamic vesicle-like structures in YFP-TTN5^{Q70L} seedlings in the root tip. Time series of YFP fluorescent signals in dynamic vesicle-like structures of YFP-TTN5^{Q70L} Arabidopsis seedlings recorded *via* fluorescence confocal microscopy. Fluorescence signals were present in cells of the root tip but without clear mobility or vesicle-like structures as in the cotyledons, hypocotyls and root hair zone. Experiment was performed once. Scale bar 50 µm.

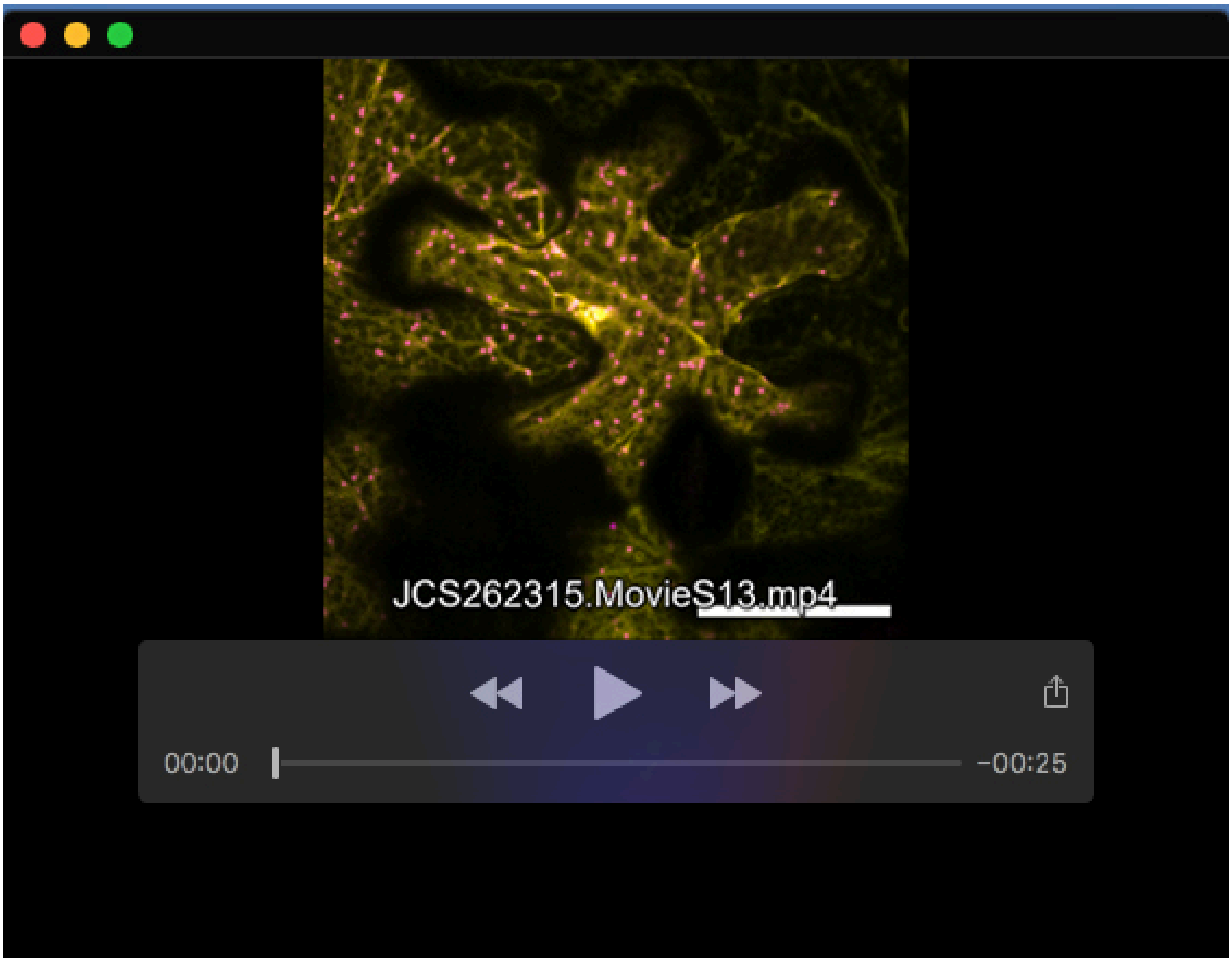

Movie 13. YFP fluorescence signals are present in dynamic vesicle-like structures in YFP-TTN5 expressing *N. benthamiana* pavement cells. Time series of YFP fluorescence signals together with *cis*-Golgi marker GmMan1-mCherry. GmMan1-positive Golgi stacks showed movement in *N. benthamiana* epidermal cells together with YFP fluorescence upon transient transformation with YFP-TTN5 construct. GmMan1 is described with a stop-and-go directed movement mediated by the actino-myosin system (Nebenführ 1999) and similarly it might be the case for YFP-TTN5 signals based on the colocalization. Hence fluorescence signals were present in a comparable manner to Arabidopsis seedlings. Experiment was performed once in three replicates (n = 3). Scale bar 50 µm.

Journal of Cell Science • Supplementary information

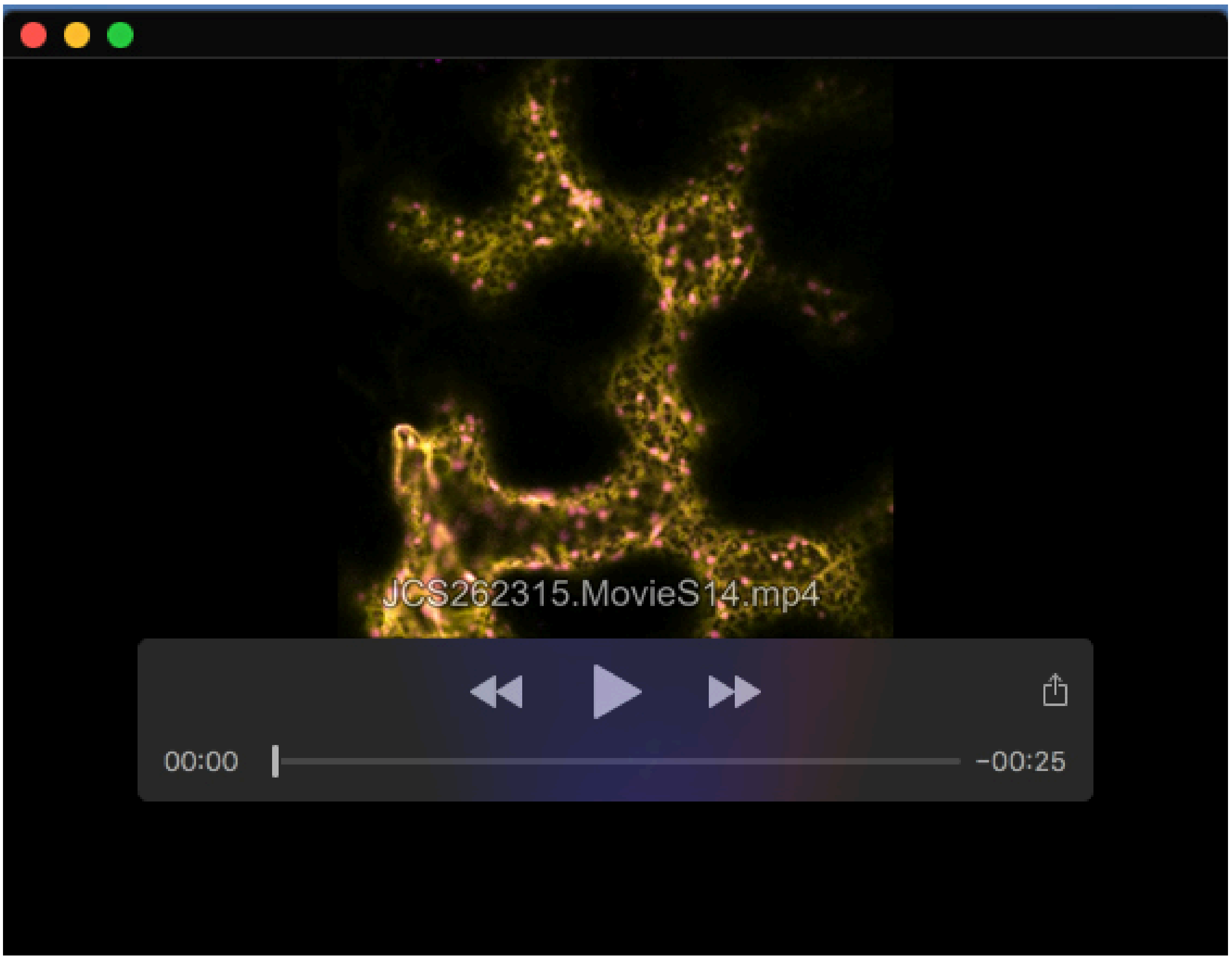

Movie 14. YFP fluorescence signals in YFP-TTN5^{T30N} expressing *N. benthamiana* pavement cells showed slowed down movement of dynamic vesicle-like structures. Time series of YFP fluorescence signals together with *cis*-Golgi marker GmMan1-mCherry. GmMan1-positive Golgi stacks showed movement in *N. benthamiana* epidermal cells together with YFP fluorescence upon transient transformation with YFP-TTN5^{T30N} construct. GmMan1 is described with a stop-and-go directed movement mediated by the actino-myosin system (Nebenführ 1999) and similarly it might be the case for YFP-TTN5^{T30N} signals based on the colocalization. Note that mobility of YFP fluorescence and GmMan1-mCherry signal in YFP-TTN5^{T30N} transformed leaf discs differed by a slower or aborted motion compared to YFP-TTN5 (Movie S13) and YFP-TTN5^{Q70L} (Movie S15), but similar as in stable YFP-TTN5^{T30N} Arabidopsis lines in the hypocotyl (Movie S5). Hence fluorescence signals were present in a comparable manner to Arabidopsis seedlings. Experiment was performed once in three replicates (n = 3). Scale bar 50 µm.

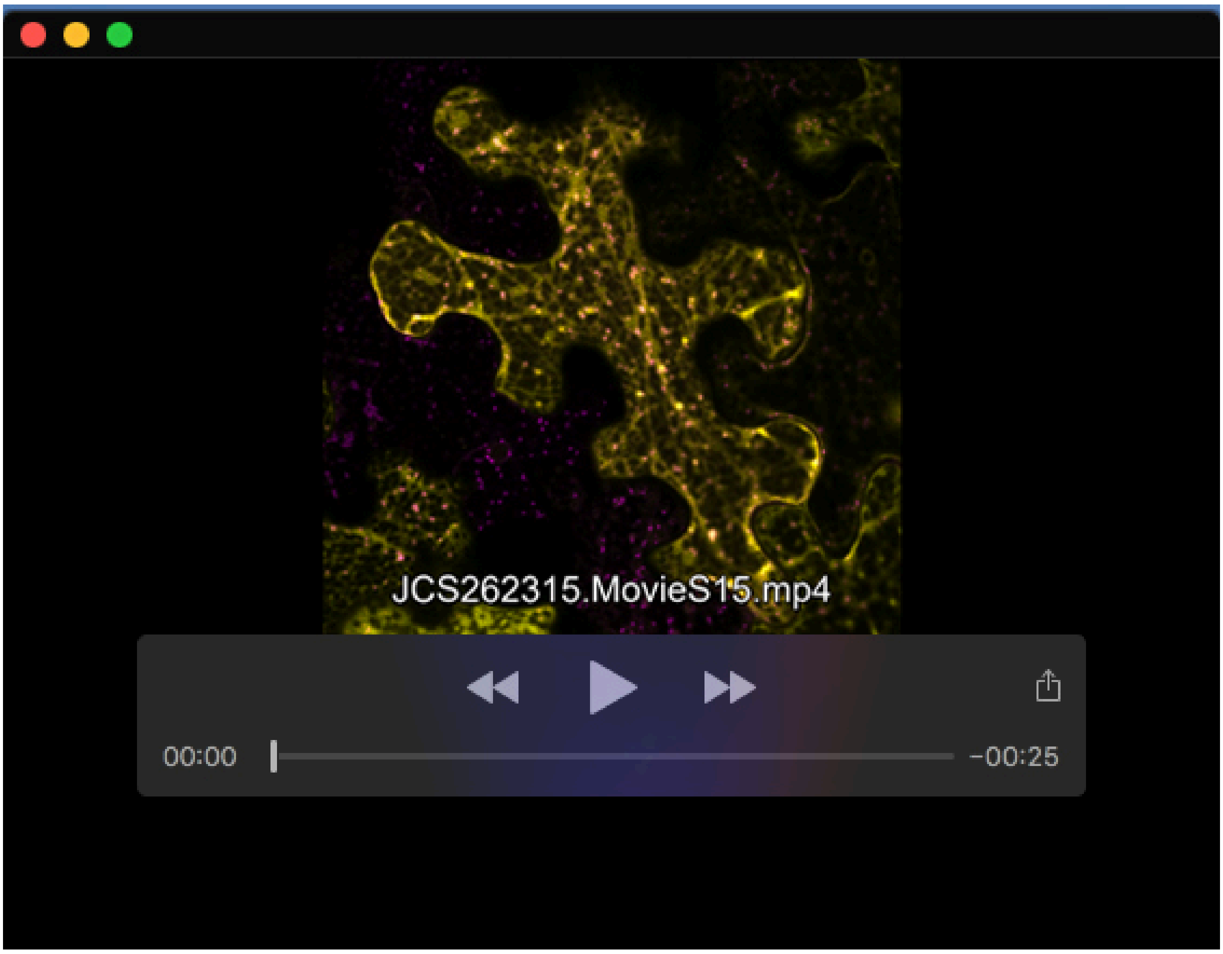

Movie 15. YFP fluorescence signals are present in dynamic vesicle-like structures in YFP-TTN5^{Q70L} expressing *N. benthamiana* pavement cells. Time series of YFP fluorescence signals together with *cis*-Golgi marker GmMan1-mCherry. GmMan1-positive Golgi stacks showed movement in *N. benthamiana* epidermal cells together with YFP fluorescence upon transient transformation with YFP-TTN5^{Q70L} construct. GmMan1 is described with a stop-and-go directed movement mediated by the actino-myosin system (Nebenführ 1999) and similarly it might be the case for YFP-TTN5 signals based on the colocalization. Hence fluorescence signals were present in a comparable manner to Arabidopsis seedlings. Experiment was performed once in two replicates (n = 2). Scale bar 50 µm.

Journal of Cell Science • Supplementary information

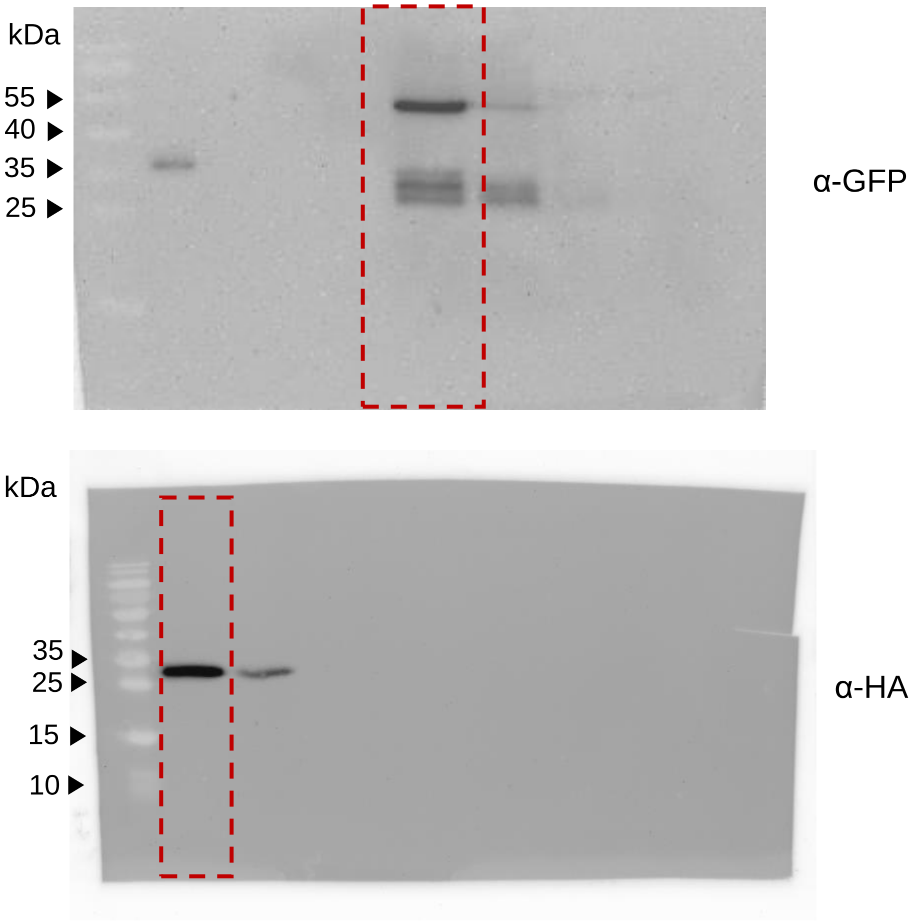

Fig. S9. Blot transparency

Journal of Cell Science • Supplementary information
