# Supplementary material for: Characterization of the small Arabidopsis thaliana GTPase and ADP-ribosylation factor-like 2 protein TITAN 5
Source: J Cell Sci. 2024 Aug 14;137(15):jcs262315. doi: 10.1242/jcs.262315 (PMC11361645; doi:10.1242/jcs.262315)
Supplement: Supplementary file 1 [file joces-137-262315_review_history.pdf]

Characterization of the small *Arabidopsis thaliana* GTPase and ADP-ribosylation factor-like 2 protein TITAN 5

Inga Mohr, Amin Mirzaiebadizi, Sibaji K. Sanyal, Pichaporn Chuenban, Mohammad R. Ahmadian, Rumen Ivanov and Petra Bauer

DOI: 10.1242/jcs.262315

Editor: Michael Way

Review timeline
Submission to Review Commons: 6 May 2023
Submission to Journal of Cell Science: 31 May 2024
Accepted: 3 June 2024

Reviewer 1

Evidence, reproducibility and clarity

The manuscript from Morh and collaborators reports the characterization of an ARF-like GTPase of Arabidopsis. Small GTPases of the ARF family play crucial role in intracellular trafficking and plant physiology. The ARF-like proteins are poorly addressed in Arabidopsis while they could reveal completely different function than the canonical known ARF proteins. Thus, the aim of the study is important and could be of interest to a wide range of plant scientists. I am impressed by the biochemical characterization of the TTN5 protein and its mutated versions, this is clearly a very nice point of the paper and allows for proper interpretations of the other results. However, I was much less convinced on the cell biology part of this manuscript and aside from the subcellular localization of the TTN5 I think the paper would benefit from a more functional angle. Below are my comments to improve the manuscript:

1. In the different pictures and movies, TTN5 is quite clearly appearing as a typical ER-like pattern. The pattern of localization further extends to dotty-like structures and structures labeled only at the periphery of the structure, with a depletion of fluorescence inside the structure. These observations raise several points. First, the ER pattern is never mentioned in the manuscript while I think it can be clearly observed. Given that the YFP-TTN5 construct is not functional (the mutant phenotype is not rescued) the ER-localization could be due to the retention at the ER due to quality control. The HA-TTN5 construct is functional but to me its localization shows a quite different pattern from the YFP version, I do not see the ER for example or the periphery-labeled structures. In this case, it will be a crucial point to perform co-localization experiments between HA-TTN5 and organelles markers to confirm that the functional TTN5 construct is labeling the Golgi and MVBs, as does the non-functional one. I am also quite sure that a co-localization between YFP-TTN5 and HA-TTN5 will not completely match... The ER is contacting so many organelles that the localization of YFP- TTN5 might not reflects the real location of the protein.
2. What are the structures with TTN5 fluorescence depleted at the center that appear in control conditions? They look different from the Golgi labeled by Man1 but similar to MVBs upon wortmannin treatment, except that in control conditions MVBs never appear like this. Are they related to any kind of vacuolar structures that would be involved in quality control- induced degradation of non-functional proteins?
3. The fluorescence at nucleus could be due to a proportion of YFP-TTN5 that is degraded and released free-GFP, a western-blot of the membrane fraction vs the cytosolic fraction could help solving this issue.

© 2024. Published by The Company of Biologists under the terms of the Creative Commons Attribution License (https://creativecommons.org/licenses/by/4.0/).

4. It is not so easy to conclude from the co-localization experiments. The confocal pictures are not always of high quality, some of them appear blurry. The Golgi localization looks convincing, but the BFA experiments are not that clear. The MVB localization is pretty convincing but the images are blurry. An issue is the quantification of the co-localizations. Several methods were employed but they do not provide consistent results. As for the object- based co-localization method, the authors employ in the text co-localization result either base on the % of YFP-labeled structures or the % of mCherry/mRFP-labeled structures, but the results are not going always in the same direction. For example, the proportion of YFP- TTN5 that co-localize with MVBs is not so different between WT and mutated version but the proportion of MVBs that co-localize with TTN5 is largely increased in the Q70L mutant. Thus it is quite difficult to interpret homogenously and in an unbiased way these results. Moreover, the results coming from the centroid-based method were presented in a table rather than a graph, I think here the authors wanted to hide the huge standard deviation of these results, what is the statistical meaning of these results?
5. The use of FM4-64 to address the vacuolar trafficking is a hazardous, FM4-64 allows the tracking of endocytosis but does not say anything on vacuolar degradation targeting and even less on the potential function of TTN5 in endosomal vacuolar targeting. Similarly, TTN5, even if localized at the Golgi, is not necessarily function in Golgi-trafficking.
6. The manuscript lacks in its present shape of functional evidences for a role of TTN5 in any trafficking steps. I understand that the KO mutant is lethal but what are the phenotypes of the Q70L and T30N mutant plants? What is the seedling phenotype, how are the Golgi and MVBs looking like in these mutants? Do the Q70L or T30N mutants perturbed the trafficking of any cargos?

Significance

In conclusion, I think this manuscript is a good biochemical description of an ARF-like protein but it would need to be strengthen on the cell biology and functional sides. Nonetheless, provided these limitations fixed, this manuscript would advance our knowledge of small GTPases in plants. The major conceptual advance of that study is to provide a non-canonical behavior of the active/inactive cycle dynamics for a small-GTPase. Of course this dynamic probably has an impact on TTN5 function and involvement in trafficking, although this remains to be fully demonstrated. Provided a substantial amount of additional experiments to support the claims of that study, this study could be of general interest for scientist working in the trafficking field.

Reviewer 2

Evidence, reproducibility and clarity

The manuscript by Mohr and colleagues characterizes the Arabidopsis predicted small GTPase TITAN5 in both biochemical and cell biology contexts using in vitro and in planta techniques. In the first half of the manuscript, the authors use in vitro nucleotide exchange assays to characterise the GTPase activity and nucleotide binding properties of TITAN5 and two mutant variants of it. The in vitro data they produce indicates that TITAN5 does indeed have general GTPase and nucleotide binding capability that would be expected for a protein predicted to be a small GTPase. Interestingly, the authors show that TITAN5 favors a GTP- bound form, which is different to many other characterized GTPases that favor GDP-binding. The authors follow their biochemical characterisation of TITAN with in planta experiments characterizing TITAN5 and its mutant variants association with the plant endomembrane system, both by stable expression in Arabidopsis and transient expression in N.benthamiana.

The strength of this manuscript is in its in vitro biochemical characterisation of TITAN5 and variants. I am not an expert on in vitro GTPase characterisation and so cannot comment specifically on the assays they have used, but generally speaking this appears to have been well done, and the authors are to be commended for it. In vitro characterisation of plant small GTPases is uncommon, and much of our knowledge is inferred for work on animal or yeast GTPases, so this will be a useful addition to the plant community in general, especially as TITAN5 is an essential gene. The in planta data that follows is sadly not as compelling as the biochemical

© 2024. Published by The Company of Biologists under the terms of the Creative Commons Attribution License (https://creativecommons.org/licenses/by/4.0/).

data, and suffers from several weaknesses. I would encourage the authors to consider trying to improve the quality of the in planta data in general. If improved and then combined with the biochemical aspects of the paper, this has the potential to make a nice addition to plant small GTPase and endomembrane literature.
The manuscript is generally well written and includes the relevant literature.

Major issues:

- The authors make use of a p35s: YFP-TTN5 construct (and its mutant variants) both stably in Arabidopsis and transiently in N.benthamiana. I know from personal experience that expressing small GTPases from non-endogenous promoters and in transient expression systems can give very different results to when working from endogenous promoters/using immunolocalization in stable expression systems. Strong over-expression could for example explain why the authors see high 'cytosolic' levels of YFP-TTN5. It is therefore questionable how much of the in planta localisation data presented using p35S and expression in tobacco is of true relevance to the biological function of TITAN5. The authors do present some immunolocalization data of HA3-TTN5 in Arabidopsis, but this is fairly limited and it is very difficult in its current form to use this to identify whether the data from YFP-TTN5 in Arabidopsis and tobacco can be corroborated. I would encourage the authors to consider expanding the immunolocalization data they present to validate their findings in tobacco.
- Many of the confocal images presented are of poor quality, particularly those from N.benthamiana.
- The authors in some places see YFP-TTN5 in cell nuclei. This could be a result of YFP-cleavage rather than genuine nuclear localisation of YFP-TTN5, but the authors do not present western blots to check for this.
- That YFP-TTN5 fails to rescue the ttn5 mutant indicates that YFP-tagged TTN5 may not be functional. If the authors cannot corroborate the YFP-TTN5 localisation pattern with that of HA3-TTN5 via immunolocalization, then the fact that YFP-TTN5 may not be functional calls into question the biological relevance of YFP-TTN5's localisation pattern.
- Without a cell wall label/dye, the plasmolysis data presented in Figure 5 is hard to visualize.

Minor issues:

- In some of the presented N.benthamiana images, it looks like YFP-TTN5 may be partially ER-localised. However, co-localisation with an ER marker is not presented.
- There is some inconsistency within the N.benthamiana images. For example, compare Figure 4C of YFP-TTN5T30N to Figure 4O of YFP-TTN5T30N. Figure 4O is presented as being significant because wortmannin-induced swollen ARA7 compartments are labelled by YFP-TTN5T30N. However, structures very similar to these can already been seen in Figure 4C, which is apparently an unrelated experiment. This, to my mind, is likely a result of the very different expression levels between different cells that can be produced by transient expression in N.benthamiana.

Referees cross-commenting

It seems that all of the reviewers have converged on the conclusion that the in planta characterisation of TTN5 is insufficient to be of substantial interest to the field, highlighting the fact that major improvements are required to strengthen this part of the manuscript and increase its relevance.

Significance

General assessment: the strengths of this work are in its in vitro characterisation of TITAN5, however, the in planta characterisation lacks depth.

Significance: the in vitro characterisation of TITAN5 is commendable as such work is lacking for plant GTPases. However, the significance of the work would be boosted substantially by better in planta characterisation, which is where most the most broad interest will lie.

My expertise: my expertise is in in planta characterisation of small GTPases and their interactors.

© 2024. Published by The Company of Biologists under the terms of the Creative Commons Attribution License (https://creativecommons.org/licenses/by/4.0/).

Reviewer 3

Evidence, reproducibility and clarity

Summary:

Cellular traffic is an important and well-studied biological process in animal and plant systems. While components involved in transport are known the mechanism by which these components control activity or destination remains to be studied. A critical step in regulating traffic is proper budding and tethering of vesicles. A critical component in determining this step is a family proteins with GTPase activity, which act as switches facilitating vesicle interaction between proteins, or cytoskeleton. The current manuscript by Mohr and colleagues have characterized a small GTPase TITAN5 (TTN5) and identified two residues Gln70 and Thr30 in the protein which they propose to have functional roles. The authors catalogue the localization, GTP hydrolytic activity, and discuss putative functions of TTN5 and the mutants.

Major comments:

The core of the manuscript, which is descriptive characterization of TTN5, lies in reliably demonstrating putative roles. While the GTP hydrolysis rates are well-quantified (though the claims need to be toned down), the microscopy data especially the association of TTN5 with different endomembrane compartments is not convincing due to the quality (low resolution) of the figures submitted. The manuscript text is difficult to navigate due to repetition and inconsistency in the order that the mutants are referred. I am requesting additional experiments which should be feasible considering the authors have all the materials required to perform the experiments and obtain high-quality images which support their claims.

1. In general the figure quality needs to be improved for all microscopy images. I would suggest that the authors highlight 1-2 individual cells to make their point and use the current images as supplementary to establish a broader spread.
- a. Fig. S1 lacks clarity.
- b. For the supplementary videos, it is difficult to determine if punctate structures are moving or is it cytoplasmic streaming? Could this be done with a co-localized marker? Considering that such markers have been used later in Fig. 4?
- c. It would be good if the speed of movement is quantified, if the authors want to retain the current claims in results and the discussion.
2. Fig.2
- a. I am not sure what the unit / scale is in Fig. 2D/E if each parameter (Kon, Koff, and Kd) are individually plotted? Could the authors please clarify/simplify this panel?
- b. Are panels D and E representing values for mdGDP and GppNHP? This is not very clear from the figure legend.
3. Fig. 3
- a. Same comments as in para above - improve resolution fo images, concentrate on a few selected cells, if required use an inset figure to zoom-in to specific compartments.
- b. Please provide the non-fluorescent channel images to understand cell topography
- c. Is the nuclear localization seen in transient expression (panel L-N) an artefact? If so, this needs to be mentioned in the text.
4. Fig. 4 - In addition to the points made for Fig. 3
- a. The authors should consider reducing gain/exposure to improve image clarity. Especially for the punctate structures, which are difficult to observe in TTN5, likely because of the cytoplasmic localization as well.
- b. Reducing Agrobacterial load could be considered. OD of 0.4 is a bit much, 0.1 or even 0.05 could be tried. If available try expression in N. tabaccum, which is more amenable to microscopy. However, this is OPTIONAL, benthamiana should suffice.
- c. A standard norm now is to establish the level of colocalization is by quantifying a pearson's or Mander's correlation. Which I believe has been done in the text, I didn't find a plot representing the same? Could the data (which the authors already have) be plotted alongwith "n"

© 2024. Published by The Company of Biologists under the terms of the Creative Commons Attribution License (https://creativecommons.org/licenses/by/4.0/).

as a table or graph?
- d. The cartoons for the action of chemicals are useful, but need a bit more clarity.
5. Fig. 5
- a. does the Q70L mutant show reduced endocytosis ?
6. The main text needs to be organized in a way that a reader can separate what is the hypothesis/assumption from actual results and conclusions (see lines #143-149).
7. The text is repeated in multiple places, while I understand that this is not plagiarism, the repetitiveness makes it difficult to read and understand the text. I highlight a couple of examples here, but please check the whole text thoroughly and edit/delete as necessary.
- a. Lines #124-125 with Lines #149-151
- b. Lines #140-143
8. Could the authors elaborate on whether there are plan homologs of TTN5? Also, have other ARF/ARLs been compared to TTN5 beyond HsARF1?
9. On a related note, a major problem I have with these kinetic values is the assumption of significance or not. For eg. Line#180 the values represent and 2 and 6-fold increase, if these numbers do not matter can a significance threshold be applied so as to understand how much fold-change is appreciable?
10. Another issue with the kinetic measurements is the significance levels. Line #198-201. The three proteins are claimed to have similar values and in the nnext line, the Q70L mutant is claimed to be high.
11. Provide data for conclusion in line#214-215
12. How were the mutants studied here identified? random mutation or was it directed based on qualified assumptions?
13. Could more simplification be provided for deifitinition of Kon/Koff values. And can these values be compared between mutants directly?
14. Data provided are not convincing to claim that both the mutant forms have lower association with the Golgi.
15. IN general the Authors should strongly consider the claims made in the manuscript. For eg. "This study lays the foundation for studying the functional relationships of this small GTPase" (line 125) is unqualified as this is true for every protein ever studied and published. Considering that TTN was not isolated/identified in this study for the first time this claim doesn't stand.
- a. Line #185 - "characterestics of a dominant-negative. " What is this based on? From the text it is not clear what are the paremeters. Considering that no complementation phenotypes have been presented, this is a far-fetched claim
- b. The claims in Line #224-227 are exaggerated. Please tone down or delete
- c. Line#488-489 - This conclusion is not really supported. At best Authors can claim that TTN5 is associated with trafficking components, but the functional relevance of this association is not determined.

Minor comments:

1. Line #95 - " This rolein vesicle." - please clarify which role?
2. Line #168 - "we did not observed" please change to "not able to measure/quantify"
3. Line#179 - ARF# is human for Arabidopsis?
4. Line #181 - compared to what is the 10-fold difference?
5. Lines #314-323 - are diffciult to understand, consider reframing. Same goes for the conclusion following these lines.
6. Authors might consider a longer BFA treatment (3-4h) to see more clearer ER-Golgi fusion (BFA bodies)

Referees cross-commenting

I agree with both my co-reviewers that the manuscript needs substantial improvement in its cell biology based experiments and conclusions thereof. I think the concensus of all reviewers points to weakness in the in-planta experiments which needs to be addressed to understand and characterize TTN5, which is the main goal of the manuscript.

Significance

© 2024. Published by The Company of Biologists under the terms of the Creative Commons Attribution License (https://creativecommons.org/licenses/by/4.0/).

The manuscript has general significance in understanding the role of small GTPases which are understudied. Although the manuscript does not advance the field of either intracellular trafficking or organization it holds significance in attempting to characterize proteins involved, which is a prerequisite for further functional studies.

Author response to reviewers' comments

Reply to reviewer comments

We extend our gratitude to the reviewers for their time and valuable feedback on our manuscript. We especially appreciate the insightful suggestions that have significantly contributed to refining our work and elucidating our findings. With the revisions made to the text and the inclusion of new experimental data, we believe our manuscript now effectively addresses all reviewer comments. We eagerly await your evaluation of our revised submission.

Small ARF-like GTPases play fundamental roles in dynamic signaling processes linked with vesicular trafficking in eukaryotes. Despite of their evolutionary conservation, there is little known about the ARF-like GTPase functions in plants. Our manuscript reports the biochemical and cell biological characterization of the small ARF-like GTPase TTN5 from the model plant Arabidopsis thaliana. *Fundamental investigations like ours are mostly lacking for ARF and ARL GTPases in Arabidopsis.*

We employed fluorescence-based enzymatic assays suited to uncover different types of the very rapid GTPase activities for TTN5. The experimental findings are now illustrated in a more comprehensive modified Figure 2 and in the form of a summary of the GTPase activities for TTN5 and its mutant variants in the NEW Figure 7A in the Discussion part. Taken together, we found that TTN5 is a non-classical GTPase based on its enzymatic kinetics. The reviewers appreciated these findings and highlighted them as being „impressive in vitro *biochemical characterization“ and “major conceptual advance”. Since such experiments are “uncommon” for being conducted with plant GTPases, reviewers regarded this analysis as “useful addition to the plant community in general”. The significance of these findings is given by the circumstance that „the ARF-like proteins are poorly addressed in Arabidopsis while they could reveal completely different function than the canonical known ARF proteins“. Reviewers saw here clearly a “strength” of the manuscript.*

With regard to the cell biological investigation and initial assessment of cell physiological roles of TTN5, we now provide requested additional evidence. First of all, we provide NEW data on the localization of TTN5 by immunolocalization using a complementing HA3-TTN5 construct, supporting our initial suggestions that TTN5 may be associated with vesicles and processes of the endomembrane system. The previous preprint version had left the reviewers „less convinced“ of cell biological data due to the lack of complementation of our YFP-TTN5 construct, lack of Western blot data and the low resolution of microscopic images. We fully agree that these points were of concern and needed to be addressed. We have therefore intensively worked on these „weaknesses“ and present now a more detailed whole-mount immunostaining series with the complementing HA3-TTN5 transgenic line (NEW Figure 4, NEW Figure 3P), Western blot data (NEW Supplementary Figures S7C and D), and we will provide all original images upon publication of our manuscript at BioImage Archives which will provide the high quality for re-analysis. BioImage Archives is an online storage for biological image data associated with a peer-reviewed publication. This way, readers will be able to inspect each image in detail. The immunolocalization data are of particular importance as they indicate that HA3-TTN5 can be associated with punctate vesicle structures and BFA bodies as seen with YFP studies of YFP-TTN5 seedlings. We have re-phrased very carefully and emphasized those localization patterns which are backed up by immunostaining and YFP fluorescence detection of YFP-TTN5 signals. To improve the comprehension, the findings are summarized in a schematic overview in NEW Figure 7B of the Discussion. We have also addressed all other comments related to the cell biological experiments to “provide the substantial improvement” that had been requested. We emphasize that we found two cell physiological phenotypes for the TTN5^{T30N} mutant. YFP-TTN5^{T30N} confers

© 2024. Published by The Company of Biologists under the terms of the Creative Commons Attribution License (https://creativecommons.org/licenses/by/4.0/).

phenotypes, which are differing mobility of the fluorescent vesicles in the epidermis of hypocotyls (see Video material and NEW Supplementary Video Material S1M-O), and a root growth phenotype of transgenic HA3-TTN5^{T30N} seedlings (NEW Figure 3O). We explain the cell physiological phenotypes in relation to enzymatic GTPase data. These findings convince us of the validity of the YFP-TTN5 analysis indicative of TTN5 localization.

We are deeply thankful to the reviewers for judging our manuscript as "generally well written", "important" and "of interest to a wide range of plant scientists" and "for scientists working in the trafficking field" as it "holds significance" and will form the basis for future functional studies of TTN5.

We prepared very carefully our revised manuscript in which we address all reviewer comments one by one. Please find our revision and our detailed rebuttal to all reviewer comments below. Changes in the revised version are highlighted by yellow and green color. In the "revised version with highlighted changes".

With these adjustments, we hope that our peer-reviewed study will receive a positive response. We are looking forward to your evaluation of our revised manuscript and thank you in advance, Sincerely

Petra Bauer and Inga Mohr on behalf of all authors

Reviewer #1 (Evidence, reproducibility and clarity (Required)):

The manuscript from Mohr and collaborators reports the characterization of an ARF-like GTPase of Arabidopsis. Small GTPases of the ARF family play crucial role in intracellular trafficking and plant physiology. The ARF-like proteins are poorly addressed in Arabidopsis while they could reveal completely different function than the canonical known ARF proteins. Thus, the aim of the study is important and could be of interest to a wide range of plant scientists. I am impressed by the biochemical characterization of the TTN5 protein and its mutated versions, this is clearly a very nice point of the paper and allows for proper interpretations of the other results. However, I was much less convinced on the cell biology part of this manuscript and aside from the subcellular localization of the TTN5 I think the paper would benefit from a more functional angle. Below are my comments to improve the manuscript:

1. In the different pictures and movies, TTN5 is quite clearly appearing as a typical ER-like pattern. The pattern of localization further extends to dotty-like structures and structures labeled only at the periphery of the structure, with a depletion of fluorescence inside the structure. These observations raise several points. First, the ER pattern is never mentioned in the manuscript while I think it can be clearly observed. Given that the YFP-TTN5 construct is not functional (the mutant phenotype is not rescued) the ER-localization could be due to the retention at the ER due to quality control. The HA-TTN5 construct is functional but to me its localization shows a quite different pattern from the YFP version, I do not see the ER for example or the periphery-labeled structures. In this case, it will be a crucial point to perform co-localization experiments between HA-TTN5 and organelles markers to confirm that the functional TTN5 construct is labeling the Golgi and MVBs, as does the non-functional one. I am also quite sure that a co-localization between YFP-TTN5 and HA-TTN5 will not completely match... The ER is contacting so many organelles that the localization of YFP-TTN5 might not reflects the real location of the protein.

Our response:

At first, we like to state that specific detection of intracellular localization of plant proteins in plant cells is generally technically very difficult, when the protein abundance is not overly high. In this revised version, we extended immunostaining analysis to different membrane compartments, including now immunostaining of complementing HA3-TTN5 in the absence and presence of BFA, along with immunodetection of ARF1 and FM4-64 labeling in roots (NEW Figure 3P, NEW Figure 4A, B). In the revised version, we focus the analysis and conclusions on the fluorescence patterns that overlap between YFP-TTN5 detection and HA3-TTN5 immunodetection. With this, we can be most confident about subcellular TTN5 localization. Please find this NEW text in the Result section

© 2024. Published by The Company of Biologists under the terms of the Creative Commons Attribution License (https://creativecommons.org/licenses/by/4.0/).

(starting Line 323):

„For a more detailed investigation of HA3-TTN5 subcellular localization, we then performed co-immunofluorescence staining with an Alexa 488-labeled antibody recognizing the Golgi and TGN marker ARF1, while detecting HA3-TTN5 with an Alexa 555-labeled antibody (Robinson et al. 2011, Singh et al. 2018) (Figure 4A). ARF1-Alexa 488 staining was clearly visible in punctate structures representing presumably Golgi stacks (Figure 4A, Alexa 488), as previously reported (Singh et al. 2018). Similar structures were obtained for HA3-TTN5-Alexa 555 staining (Figure 4A, Alexa 555). But surprisingly, colocalization analysis demonstrated that the HA3-TTN5-labeled structures were mostly not colocalizing and thus distinct from the ARF1-labeled ones (Figure 4A). Yet the HA3-TTN5- and ARF1-labeled structures were in close proximity to each other (Figure 4A). We hypothesized that the HA3-TTN5 structures can be connected to intracellular trafficking steps. To test this, we performed brefeldin A (BFA) treatment, a commonly used tool in cell biology for preventing dynamic membrane trafficking events and vesicle transport involving the Golgi. BFA is a fungal macrocyclic lactone that leads to a loss of *cis*-cisternae and accumulation of Golgi stacks, known as BFA-induced compartments, up to the fusion of the Golgi with the ER (Ritzenthaler et al. 2002, Wang et al. 2016). For a better identification of BFA bodies, we additionally used the dye FM4-64, which can emit fluorescence in a lipophilic membrane environment. FM4-64 marks the plasma membrane in the first minutes following application to the cell, then may be endocytosed and in the presence of BFA become accumulated in BFA bodies (Bolte et al. 2004). We observed BFA bodies positive for both, HA3-TTN5-Alexa 488 and FM4-64 signals (Figure 4B). Similar patterns were observed for YFP-TTN5-derived signals in YFP-TTN5-expressing roots (Figure 4C). Hence, HA3-TTN5 and YFP-TTN5 can be present in similar subcellular membrane compartments.“

We did not find evidence that HA3-TTN5 can localize at the ER using whole-mount immunostaining (NEW Figure 3P; NEW Figure 4A, B). Hence, we are careful with describing that fluorescence at the ER, as seen in the YFP-TTN5 line (Figure 3M, N) reflects TTN5 localization. We therefore do not focus the text on the ER pattern in the Result section (starting Line 295):

„Additionally, YFP signals were also detected in a net-like pattern typical for ER localization (Figure 3M, N). (...) We also found multiple YFP bands in α-GFP Western blot analysis using YFP-TTN5 Arabidopsis seedlings. Besides the expected and strong 48 kDa YFP-TTN5 band, we observed three weak bands ranging between 26 to 35 kDa (Supplementary Figure S7C). We cannot explain the presence of these small protein bands. They might correspond to free YFP, to proteolytic products or potentially to proteins produced from aberrant transcripts with perhaps alternative translation start or stop sites. On the other side, a triple hemagglutinin- tagged HA3-TTN5 driven by the 35S promoter did complement the embryo-lethal phenotype of *ttn5-1* (Supplementary Figure S7D, E). α-HA Western blot control performed with plant material from HA3-TTN5 seedlings showed a single band at the correct size, but no band that was 13 to 18 kDa smaller (Supplementary Figure S7D). (...) We did not observe any staining in nuclei or ER when performing HA3-TTN5 immunostaining (Figure 3P; Figure 4A, B), as was the case for fluorescence signals in YFP-TTN5-expressing cells. Presumably, this can indicate that either the nuclear and ER signals seen with YFP-TTN5 correspond to the smaller proteins detected, as described above, or that immunostaining was not suited to detect them. Hence, we focused interpretation on patterns of localization overlapping between the fluorescence staining with YFP-labeled TTN5 and with HA3-TTN5 immunostaining, such as the particular signal patterns in the specific punctate membrane structures.“

And we discuss in the Discussion section (starting Line 552):

„We based the TTN5 localization data on tagging approaches with two different detection methods to enhance reliability of specific protein detection. Even though YFP-TTN5 did not complement the embryo-lethality of a *ttn5* loss of function mutant, we made several observations that suggest YFP-TTN5 signals to be meaningful at various membrane sites. We do not know why YFP-TTN5 does not complement. There could be differences in TTN5 levels and interactions in some cell types, which were hindering specifically YFP-TTN5 but not HA3- TTN5. (...) Though constitutively driven, the YFP-TTN5 expression may be delayed or insufficient at the early

© 2024. Published by The Company of Biologists under the terms of the Creative Commons Attribution License (https://creativecommons.org/licenses/by/4.0/).

embryonic stages resulting in the lack of embryo-lethal complementation. On the other hand, the very fast nucleotide exchange activity may be hindered by the presence of a large YFP-tag in comparison with the small HA3-tag which is able to rescue the embryo-lethality. The lack of complementation represents a challenge for the localization of small GTPases with rapid nucleotide exchange in plants. Despite of these limitations, we made relevant observations in our data that made us believe that YFP signals in YFP-TTN5-expressing cells at membrane sites can be meaningful.“

2. What are the structures with TTN5 fluorescence depleted at the center that appear in control conditions? They look different from the Golgi labeled by Man1 but similar to MVBs upon wortmannin treatment, except that in control conditions MVBs never appear like this. Are they related to any kind of vacuolar structures that would be involved in quality control-induced degradation of non-functional proteins?

Our response:
The reviewer certainly refers to fluorescence images from N. benthamiana *leaf epidermal cells where different circularly shaped structures are visible. In these respective structures, the fluorescent circles are depleted from fluorescence in the center, e.g. in Figure 5C, YFP-fluorescent signals in TTN5^{T30N} transformed leaf discs. We suspect that these structures can be of vacuolar origin as described for similar fluorescent rings in Tichá et al., 2020 for ANNI- GFP (reference in manuscript). The reviewer certainly does not refer to swollen MVBs that are seen following wortmannin treatment, as in Figure 5N-P, which look similar in their shape but are larger in size. Please note that we always included the control conditions, namely the images recorded before the wortmannin treatment, so that we were able to investigate the changes induced by wortmannin. Hence, we can clearly say that the structures with depleted fluorescence in the center as in Figure 5C are not wortmannin-induced swollen MVBs.To make these points clear to the reader, we added an explanation into the text (Line 385-388):*

„We also observed YFP fluorescence signals in the form of circularly shaped ring structures with a fluorescence-depleted center. These structures can be of vacuolar origin as described for similar fluorescent rings in Tichá et al. (2020) for ANNI-GFP.”

3. The fluorescence at nucleus could be due to a proportion of YFP-TTN5 that is degraded and released free-GFP, a western-blot of the membrane fraction vs the cytosolic fraction could help solving this issue.

Our response:
In an a-GFP Western blot using YFP-TTN5 Arabidopsis seedlings, we detected besides the expected and strong 48 kDa YFP-TTN5 band, three additional weak bands ranging between 26 to 35 kDa (NEW Supplementary Figure S7C). We cannot explain the presence of these small protein bands. They might correspond to free YFP, to proteolytic products or potentially to proteins expressed from aberrant transcripts. a-HA Western blot controls performed with plant material from HA3-TTN5 seedlings showed a single band at the correct size (Supplementary Figure S7D). We must therefore be cautious about nuclear TTN5 localization and we rephrased the text carefully (starting Line 300):

„We also found multiple YFP bands in α-GFP Western blot analysis using YFP-TTN5 Arabidopsis seedlings. Besides the expected and strong 48 kDa YFP-TTN5 band, we observed three weak bands ranging between 26 to 35 kDa (Supplementary Figure S7C). We cannot explain the presence of these small protein bands. They might correspond to free YFP, to proteolytic products or potentially to proteins produced from aberrant transcripts with perhaps alternative translation start or stop sites. On the other side, a triple hemagglutinin- tagged HA3-TTN5 driven by the 35S promoter did complement the embryo-lethal phenotype of *ttn5-1* (Supplementary Figure S7D, E). α-HA Western blot control performed with plant material from HA3-TTN5 seedlings showed a single band at the correct size, but no band that was 13 to 18 kDa smaller (Supplementary Figure S7D). (...) We did not observe any staining in nuclei or ER when performing HA3-TTN5 immunostaining (Figure 3P; Figure 4A, B), as was the case for fluorescence signals in YFP-TTN5-expressing cells. Presumably, this can indicate that either the nuclear and ER signals seen with YFP-

© 2024. Published by The Company of Biologists under the terms of the Creative Commons Attribution License (https://creativecommons.org/licenses/by/4.0/).

TTN5 correspond to the smaller proteins detected, as described above, or that immunostaining was not suited to detect them. Hence, we focused interpretation on patterns of localization overlapping between the fluorescence staining with YFP-labeled TTN5 and with HA3-TTN5 immunostaining, such as the particular signal patterns in the specific punctate membrane structures."

4. It is not so easy to conclude from the co-localization experiments. The confocal pictures are not always of high quality, some of them appear blurry. The Golgi localization looks convincing, but the BFA experiments are not that clear. The MVB localization is pretty convincing but the images are blurry. An issue is the quantification of the co-localizations. Several methods were employed but they do not provide consistent results. As for the object-based co-localization method, the authors employ in the text co-localization result either base on the % of YFP- labeled structures or the % of mCherry/mRFP-labeled structures, but the results are not going always in the same direction. For example, the proportion of YFP-TTN5 that co-localize with MVBs is not so different between WT and mutated version but the proportion of MVBs that co- localize with TTN5 is largely increased in the Q70L mutant. Thus it is quite difficult to interpret homogenously and in an unbiased way these results. Moreover, the results coming from the centroid-based method were presented in a table rather than a graph, I think here the authors wanted to hide the huge standard deviation of these results, what is the statistical meaning of these results?

Our response:

First of all, we like to point out that, as explained above, the BFA experiments are now more clear. We performed additional BFA treatment coupled with immunostaining using HA3-TTN5-expressing Arabidopsis seedlings and coupled with fluorescence analysis using YFP-TTN5-expressing Arabidopsis plants. In both experiments, we observed the typical BFA bodies very clearly (NEW Figure 4B, C).

Second, we like to insist that we performed colocalization very carefully and quantified the data in three different manners. We like to state that there is no general standardized procedure that best suits the idea of a colocalization pattern. Results of colocalization are represented in stem diagrams and table format, including statistical analysis. Colocalization was carried out with the ImageJ plugin JACoP for Pearson's and Overlap coefficients and based on the centroid method. The plotted Pearson's and Overlap coefficients are presented in bar diagrams in Supplementary Figure S8A and C, including statistics. The obtained values by the centroid method are represented in table format in Supplementary Figure S8B and D, which can be considered a standard method (see Ivanov et al., 2014).

Colocalization of two different fluorescence signals was performed for the two channels in a specific chosen region of interest (indicating in % the overlapping signal versus the sum of signal for each channel). The differences between the YFP/mRFP and mRFP/YFP ratios indicate that a higher percentage of ARA7-RFP signal is colocalizing with YFP-TTN5^{Q70L} signal than with the TTN5^{WT} or the TTN5^{T30N} mutant form signals, while the YFP signals have a similar overlap with ARA7-positive structures. This is not a contradiction. Presumably this answers well the questions on colocalization.

Please note that upon acceptance for publication, we will upload all original colocalization data to BioImage Archive. Hence, the high-quality data can be reanalyzed by readers.

5. The use of FM4-64 to address the vacuolar trafficking is a hazardous, FM4-64 allows the tracking of endocytosis but does not say anything on vacuolar degradation targeting and even less on the potential function of TTN5 in endosomal vacuolar targeting. Similarly, TTN5, even if localized at the Golgi, is not necessarily function in Golgi-trafficking.

Our response:
Perhaps our previous description was misleading. Thank you for pointing this out. We reformulated the text and modified the schematic representation of FM4-64 in NEW Figure 6A:

"(A), Schematic representation of progressive stages of FM4-64 localization and internalization in

© 2024. Published by The Company of Biologists under the terms of the Creative Commons Attribution License (https://creativecommons.org/licenses/by/4.0/).

a cell. FM4-64 is a lipophilic substance. After infiltration, it first localizes in the plasma membrane, at later stages it localizes to intracellular vesicles and membrane compartments. This localization pattern reflects the endocytosis process (Bolte et al. 2004)."

6. The manuscript lacks in its present shape of functional evidences for a role of TTN5 in any trafficking steps. I understand that the KO mutant is lethal but what are the phenotypes of the Q70L and T30N mutant plants? What is the seedling phenotype, how are the Golgi and MVBs looking like in these mutants? Do the Q70L or T30N mutants perturbed the trafficking of any cargos?

Our response:
We agree fully that functional evidences are interesting to assign roles for TTN5 in trafficking steps. A phenotype associated with TTN5^{T30N} and TTN5^{Q70L} is clearly meaningful.

First of all, we like to emphasize that it is incorrect that the manuscript lacks functional evidences for a role of TTN5 and the two mutants. In fact, the manuscript even highlights several functional activities that are meaningful in a cellular context. These include different types of kinetic GTPase enzyme activities, subcellular localization in planta and association with different endomembrane compartments and subcellular processes such as endocytosis. We surely agree that future research can focus even more on cell physiological aspects and the physiological functions in plants to examine the proposed roles of TTN5 in intracellular trafficking steps. For such studies, our findings are the fundamental basis.

Second, we do report phenotypes for TTN5^{T30N} that are not observed for TTN5 and TTN5^{Q70L}. From the general assumption how GTPases work, it might have been suspected at first that the TTN5^{T30N} and TTN5^{Q70L} mutant forms should result in cell phenotypes or even morphological and physiological phenotypes of plants. However, when we interpreted the GTPase enzyme data, it became clear that TTN5 behaved as a non-classical GTPase with high GTP affinity, including fast GDP dissociation and slow GTP hydrolysis rates. The consequence of this is that unlike a classical GTPase, TTN5 is most likely present in GTP- bound form in the cells in the resting state. The Q70L mutation can lead to a constitutive active GTPase with an impaired hydrolysis activity in certain types of small GTPases. However, TTN5^{Q70L} showed very similar values for nucleotide dissociation and affinity compared to TTN5^{WT} and only a slightly reduced GTP hydrolysis activity. Thus, this atypical biochemical characteristic can explain why TTN5^{Q70L} mutant-expressing plants lack a visible phenotype. The T30N mutation of certain GTPases is described in literature as a dominant-negative version forming a stable GTPase-GEF complex and subsequent prevention of substrate activation. Here, we found that TTN5^{T30N} exhibited a high GDP dissociation rate and low GDP affinity, which can indicate either a dominant-negative mutation or a fast-cycling mutation. Very interestingly, we observed a cellular phenotype of TTN5^{T30N}, namely displaying reduced mobility of YFP fluorescence signals in hypocotyls (Supplementary Video material S1E, compared with high mobility in case of other TTN5 forms in hypocotyls (Supplementary Video material S1D and F). In this revised version, we included root length data, showing that HA3- TTN5^{T30N} seedlings have shorter roots than untransformed wild type, HA3-TTN5 and partly HA3-TTN5^{Q70L} seedlings (NEW Figure 3O). Again, the seedlings of HA3-TTN5^{Q70L} mutants did not have a significantly different root growth phenotype compared to HA3-TTN5 and WT which is fitting with our biochemical data, while, very interestingly, HA3-TTN5^{T30N} seedlings had shorter roots. This root phenotype along with the hypocotyl mobility phenotype may have been caused by the altered GDP kinetics of the HA3-TTN5^{T30N} variant.

Concerning the aspect of colocalization of the mutants with the markers we show in Figure 5C, D and G, H that YFP-TTN5^{T30N}- and YFP-TTN5^{Q70L}-related signals colocalize with the Golgi marker GmMan1-mCherry. Figure 5K, L and O, P show that YFP-TTN5^{T30N} and YFP-TTN5^{Q70L}- related signals can colocalize with the MVB marker, and this may affect relevant vesicle trafficking processes and plasma membrane protein regulation involved in root cell elongation.

At present, we have not yet investigated perturbed cargo trafficking. These aspects are certainly interesting but require extensive work and testing of appropriate physiological conditions and appropriate cargo targets. We discuss future perspectives in the Discussion. We agree that such functional information is of great importance, but needs to be clarified in future studies.

© 2024. Published by The Company of Biologists under the terms of the Creative Commons Attribution License (https://creativecommons.org/licenses/by/4.0/).

Reviewer #1 (Significance (Required)):

In conclusion, I think this manuscript is a good biochemical description of an ARF-like protein but it would need to be strengthen on the cell biology and functional sides. Nonetheless, provided these limitations fixed, this manuscript would advance our knowledge of small GTPases in plants. The major conceptual advance of that study is to provide a non-canonical behavior of the active/inactive cycle dynamics for a small-GTPase. Of course this dynamic probably has an impact on TTN5 function and involvement in trafficking, although this remains to be fully demonstrated. Provided a substantial amount of additional experiments to support the claims of that study, this study could be of general interest for scientist working in the trafficking field.

Our response:

We thank reviewer 1 for the very fruitful comments. We hope that with the additional experiments, NEW Figures and NEW Supplementary Figures as well as our changes in the text, all comments by the reviewer have been addressed.

Reviewer #2 (Evidence, reproducibility and clarity (Required)):

The manuscript by Mohr and colleagues characterizes the Arabidopsis predicted small GTPase TITAN5 in both biochemical and cell biology contexts using in vitro and in planta techniques. In the first half of the manuscript, the authors use in vitro nucleotide exchange assays to characterise the GTPase activity and nucleotide binding properties of TITAN5 and two mutant variants of it. The in vitro data they produce indicates that TITAN5 does indeed have general GTPase and nucleotide binding capability that would be expected for a protein predicted to be a small GTPase. Interestingly, the authors show that TITAN5 favors a GTP- bound form, which is different to many other characterized GTPases that favor GDP-binding. The authors follow their biochemical characterisation of TITAN with in planta experiments characterizing TITAN5 and its mutant variants association with the plant endomembrane system, both by stable expression in Arabidopsis and transient expression in N.benthamiana.
The strength of this manuscript is in its in vitro biochemical characterisation of TITAN5 and variants. I am not an expert on in vitro GTPase characterisation and so cannot comment specifically on the assays they have used, but generally speaking this appears to have been well done, and the authors are to be commended for it. In vitro characterisation of plant small GTPases is uncommon, and much of our knowledge is inferred for work on animal or yeast GTPases, so this will be a useful addition to the plant community in general, especially as TITAN5 is an essential gene. The in planta data that follows is sadly not as compelling as the biochemical data, and suffers from several weaknesses. I would encourage the authors to consider trying to improve the quality of the in planta data in general. If improved and then combined with the biochemical aspects of the paper, this has the potential to make a nice addition to plant small GTPase and endomembrane literature.
The manuscript is generally well written and includes the relevant literature.

Major issues:
1. The authors make use of a p35s: YFP-TTN5 construct (and its mutant variants) both stably in Arabidopsis and transiently in N.benthamiana. I know from personal experience that expressing small GTPases from non-endogenous promoters and in transient expression systems can give very different results to when working from endogenous promoters/using immunolocalization in stable expression systems. Strong over-expression could for example explain why the authors see high 'cytosolic' levels of YFP-TTN5. It is therefore questionable how much of the in planta localisation data presented using p35S and expression in tobacco is of true relevance to the biological function of TITAN5. The authors do present some immunolocalization data of HA3-TTN5 in Arabidopsis, but this is fairly limited and it is very difficult in its current form to use this to identify whether the data from YFP-TTN5 in Arabidopsis and tobacco can be corroborated. I would encourage the authors to consider expanding the immunolocalization data they present to validate their findings in tobacco.

© 2024. Published by The Company of Biologists under the terms of the Creative Commons Attribution License (https://creativecommons.org/licenses/by/4.0/).

Our response:

We are aware that endogenous promoters may be preferred over 35S promoter. However, the two types of lines we generated with endogenous promoter did both not show fluorescent signals so that we could unfortunately not use them (not shown). Besides 35S promoter- mediated expression we were also investigating inducible expression vectors for fluorescence imaging in N. benthamiana *(not shown). Both inducible and constitutive expression showed very similar expression patterns so that we chose characterizing in detail the 35S::YFP-TTN5 fluorescence in both* N. bethamiana *and Arabidopsis.*

We have expanded immunolocalization using the HA3-TTN5 line and compare it now along with YFP fluorescence signal in YFP-TTN5 seedlings (NEW Figure 3P; NEW Figure 4).

„For a more detailed investigation of HA3-TTN5 subcellular localization, we then performed co-immunofluorescence staining with an Alexa 488-labeled antibody recognizing the Golgi and TGN marker ARF1, while detecting HA3-TTN5 with an Alexa 555-labeled antibody (Robinson et al. 2011, Singh et al. 2018) (Figure 4A). ARF1-Alexa 488 staining was clearly visible in punctate structures representing presumably Golgi stacks (Figure 4A, Alexa 488), as previously reported (Singh et al. 2018). Similar structures were obtained for HA3-TTN5-Alexa 555 staining (Figure 4A, Alexa 555). But surprisingly, colocalization analysis demonstrated that the HA3-TTN5-labeled structures were mostly not colocalizing and thus distinct from the ARF1- labeled ones (Figure 4A). Yet the HA3-TTN5- and ARF1-labeled structures were in close proximity to each other (Figure 4A). We hypothesized that the HA3-TTN5 structures can be connected to intracellular trafficking steps. To test this, we performed brefeldin A (BFA) treatment, a commonly used tool in cell biology for preventing dynamic membrane trafficking events and vesicle transport involving the Golgi. BFA is a fungal macrocyclic lactone that leads to a loss of *cis*-cisternae and accumulation of Golgi stacks, known as BFA-induced compartments, up to the fusion of the Golgi with the ER (Ritzenthaler et al. 2002, Wang et al. 2016). For a better identification of BFA bodies, we additionally used the dye FM4-64, which can emit fluorescence in a lipophilic membrane environment. FM4-64 marks the plasma membrane in the first minutes following application to the cell, then may be endocytosed and in the presence of BFA become accumulated in BFA bodies (Bolte et al. 2004). We observed BFA bodies positive for both, HA3-TTN5-Alexa 488 and FM4-64 signals (Figure 4B). Similar patterns were observed for YFP-TTN5-derived signals in YFP-TTN5-expressing roots (Figure 4C). Hence, HA3-TTN5 and YFP-TTN5 can be present in similar subcellular membrane compartments."

2. Many of the confocal images presented are of poor quality, particularly those from N.benthamiana.

Our response:

All confocal images are of high quality in their original format. To make them accessible, we will upload all raw data to BioImage Archive upon acceptance of the manuscript.

3. The authors in some places see YFP-TTN5 in cell nuclei. This could be a result of YFP- cleavage rather than genuine nuclear localisation of YFP-TTN5, but the authors do not present western blots to check for this.

Our response:

As described in our response to reviewer 1, comment 3, Fluorescence signals were detected within the nuclei of root cells of YFP-TTN5 plants, while immunostaining signals of HA3-TTN5 were not detected in the nucleus. In an a-GFP Western blot using YFP-TTN5 Arabidopsis seedlings, we detected besides the expected and strong 48 kDa YFP-TTN5 band, three additional weak bands ranging between 26 to 35 kDa (NEW Supplementary Figure S7C). We cannot explain the presence of these small protein bands. They might correspond to free YFP, to proteolytic products or

© 2024. Published by The Company of Biologists under the terms of the Creative Commons Attribution License (https://creativecommons.org/licenses/by/4.0/).

potentially to proteins expressed from aberrant transcripts. α-HA Western blot controls performed with plant material from HA3-TTN5 seedlings showed a single band at the correct size (Supplementary Figure S7D). We must therefore be cautious about nuclear TTN5 localization and we rephrased the text carefully (starting Line 300):

„We also found multiple YFP bands in α-GFP Western blot analysis using YFP-TTN5 Arabidopsis seedlings. Besides the expected and strong 48 kDa YFP-TTN5 band, we observed three weak bands ranging between 26 to 35 kDa (Supplementary Figure S7C). We cannot explain the presence of these small protein bands. They might correspond to free YFP, to proteolytic products or potentially to proteins produced from aberrant transcripts with perhaps alternative translation start or stop sites. On the other side, a triple hemagglutinin- tagged HA3-TTN5 driven by the 35S promoter did complement the embryo-lethal phenotype of *ttn5-1* (Supplementary Figure S7D, E). α-HA Western blot control performed with plant material from HA3-TTN5 seedlings showed a single band at the correct size, but no band that was 13 to 18 kDa smaller (Supplementary Figure S7D). (...) We did not observe any staining in nuclei or ER when performing HA3-TTN5 immunostaining (Figure 3P; Figure 4A, B), as was the case for fluorescence signals in YFP-TTN5-expressing cells. Presumably, this can indicate that either the nuclear and ER signals seen with YFP-TTN5 correspond to the smaller proteins detected, as described above, or that immunostaining was not suited to detect them. Hence, we focused interpretation on patterns of localization overlapping between the fluorescence staining with YFP-labeled TTN5 and with HA3-TTN5 immunostaining, such as the particular signal patterns in the specific punctate membrane structures."

4. That YFP-TTN5 fails to rescue the ttn5 mutant indicates that YFP-tagged TTN5 may not be functional. If the authors cannot corroborate the YFP-TTN5 localisation pattern with that of HA3-TTN5 via immunolocalization, then the fact that YFP-TTN5 may not be functional calls into question the biological relevance of YFP-TTN5's localisation pattern.

Our response:

This refers to your comment 1, please check this comment for a detailed response. Please also see our answer to reviewer 1, comment 1.

At first, we like to state that specific detection of intracellular localization of plant proteins in plant cells is generally technically very difficult, when the protein abundance is not overly high. In this revised version, we extended immunostaining analysis to different membrane compartments, including now immunostaining of complementing HA3-TTN5 in the absence and presence of BFA, along with immunodetection of ARF1 and FM4-64 labeling in roots (NEW Figure 3P, NEW Figure 4A, B). In the revised version, we focus the analysis and conclusions on the fluorescence patterns that overlap between YFP-TTN5 detection and HA3-TTN5 immunodetection. With this, we can be most confident about subcellular TTN5 localization. Please find this NEW text in the Result section (starting Line 323):

„For a more detailed investigation of HA3-TTN5 subcellular localization, we then performed co-immunofluorescence staining with an Alexa 488-labeled antibody recognizing the Golgi and TGN marker ARF1, while detecting HA3-TTN5 with an Alexa 555-labeled antibody (Robinson et al. 2011, Singh et al. 2018) (Figure 4A). ARF1-Alexa 488 staining was clearly visible in punctate structures representing presumably Golgi stacks (Figure 4A, Alexa 488), as previously reported (Singh et al. 2018). Similar structures were obtained for HA3-TTN5-Alexa 555 staining (Figure 4A, Alexa 555). But surprisingly, colocalization analysis demonstrated that the HA3-TTN5-labeled structures were mostly not colocalizing and thus distinct from the ARF1- labeled ones (Figure 4A). Yet the HA3-TTN5- and ARF1-labeled structures were in close proximity to each other (Figure 4A). We hypothesized that the HA3-TTN5 structures can be connected to intracellular trafficking steps. To test this, we performed brefeldin A (BFA) treatment, a commonly used tool in cell biology for preventing dynamic membrane trafficking events and vesicle transport involving the Golgi. BFA is a fungal macrocyclic lactone that leads to a loss of *cis*-cisternae and accumulation of Golgi stacks,

© 2024. Published by The Company of Biologists under the terms of the Creative Commons Attribution License (https://creativecommons.org/licenses/by/4.0/).

known as BFA-induced compartments, up to the fusion of the Golgi with the ER (Ritzenthaler et al. 2002, Wang et al. 2016). For a better identification of BFA bodies, we additionally used the dye FM4-64, which can emit fluorescence in a lipophilic membrane environment. FM4-64 marks the plasma membrane in the first minutes following application to the cell, then may be endocytosed and in the presence of BFA become accumulated in BFA bodies (Bolte et al. 2004). We observed BFA bodies positive for both, HA3-TTN5-Alexa 488 and FM4-64 signals (Figure 4B). Similar patterns were observed for YFP-TTN5-derived signals in YFP-TTN5-expressing roots (Figure 4C). Hence, HA3-TTN5 and YFP-TTN5 can be present in similar subcellular membrane compartments.“

We did not find evidence that HA3-TTN5 can localize at the ER using whole-mount immunostaining (NEW Figure 3P; NEW Figure 4A, B). Hence, we are careful with describing that fluorescence at the ER, as seen in the YFP-TTN5 line (Figure 3M, N) reflects TTN5 localization. We therefore do not focus the text on the ER pattern in the Result section (starting Line 295):

„Additionally, YFP signals were also detected in a net-like pattern typical for ER localization (Figure 3M, N). (...) We also found multiple YFP bands in α-GFP Western blot analysis using YFP-TTN5 Arabidopsis seedlings. Besides the expected and strong 48 kDa YFP-TTN5 band, we observed three weak bands ranging between 26 to 35 kDa (Supplementary Figure S7C). We cannot explain the presence of these small protein bands. They might correspond to free YFP, to proteolytic products or potentially to proteins produced from aberrant transcripts with perhaps alternative translation start or stop sites. On the other side, a triple hemagglutinin- tagged HA3-TTN5 driven by the 35S promoter did complement the embryo-lethal phenotype of *ttn5-1* (Supplementary Figure S7D, E). α-HA Western blot control performed with plant material from HA3-TTN5 seedlings showed a single band at the correct size, but no band that was 13 to 18 kDa smaller (Supplementary Figure S7D). (...) We did not observe any staining in nuclei or ER when performing HA3-TTN5 immunostaining (Figure 3P; Figure 4A, B), as was the case for fluorescence signals in YFP-TTN5-expressing cells. Presumably, this can indicate that either the nuclear and ER signals seen with YFP-TTN5 correspond to the smaller proteins detected, as described above, or that immunostaining was not suited to detect them. Hence, we focused interpretation on patterns of localization overlapping between the fluorescence staining with YFP-labeled TTN5 and with HA3-TTN5 immunostaining, such as the particular signal patterns in the specific punctate membrane structures.“

And we discuss in the Discussion section (starting Line 552):

„We based the TTN5 localization data on tagging approaches with two different detection methods to enhance reliability of specific protein detection. Even though YFP-TTN5 did not complement the embryo-lethality of a *ttn5* loss of function mutant, we made several observations that suggest YFP-TTN5 signals to be meaningful at various membrane sites. We do not know why YFP-TTN5 does not complement. There could be differences in TTN5 levels and interactions in some cell types, which were hindering specifically YFP-TTN5 but not HA3- TTN5. (...) Though constitutively driven, the YFP-TTN5 expression may be delayed or insufficient at the early embryonic stages resulting in the lack of embryo-lethal complementation. On the other hand, the very fast nucleotide exchange activity may be hindered by the presence of a large YFP-tag in comparison with the small HA3-tag which is able to rescue the embryo-lethality. The lack of complementation represents a challenge for the localization of small GTPases with rapid nucleotide exchange in plants. Despite of these limitations, we made relevant observations in our data that made us believe that YFP signals in YFP-TTN5-expressing cells at membrane sites can be meaningful.“

5. Without a cell wall label/dye, the plasmolysis data presented in Figure 5 is hard to visualize.

Our response:

Figure 6E-G (previously Fig. 5) show the results of plasmolysis experiments with YFP-TTN5 and the two mutant variant constructs. It is clearly possible to observe plasmolysis when focusing on the Hechtian strands. Hechtian strands are formed due to the retraction of the protoplast as a result of the osmotic pressure by the added mannitol solution. Hechtian strands consist of PM which remained in contact with the cell wall, visible as thin filamental structures. We stained the

© 2024. Published by The Company of Biologists under the terms of the Creative Commons Attribution License (https://creativecommons.org/licenses/by/4.0/).

PM and the Hechtian strands by the PM dye FM4-64. This is similary done in Yoneda et al., 2020. We could detect in the YFP-TTN5-transformed cells, colocalization with the YFP channels and the PM dye in filamental structures between two neighbouring FM4-64- labelled PMs. Although an additional labeling of the cell wall may further indicate plasmolysis, it is not needed here.

Please consider that we will upload all original image data to BioImage Archive so that a detailed re-investigation of the images can be done.

Minor issues:

6. In some of the presented N.benthamiana images, it looks like YFP-TTN5 may be partially ER-localised. However, co-localisation with an ER marker is not presented.

Our response:

Referring to our response to comments 1 and 3 of reviewer 2 and to comment 1 of reviewer 1: We did not find evidence that HA3-TTN5 can localize at the ER using whole-mount immunostaining (NEW Figure 3P; NEW Figure 4A, B). Hence, we are careful with describing that fluorescence at the ER, as seen in the YFP-TTN5 line (Figure 3M, N) reflects TTN5 localization. We therefore do not focus the text on the ER pattern in the Result section (starting Line 295):

„Additionally, YFP signals were also detected in a net-like pattern typical for ER localization (Figure 3M, N). (...) We also found multiple YFP bands in α-GFP Western blot analysis using YFP-TTN5 Arabidopsis seedlings. Besides the expected and strong 48 kDa YFP-TTN5 band, we observed three weak bands ranging between 26 to 35 kDa (Supplementary Figure S7C). We cannot explain the presence of these small protein bands. They might correspond to free YFP, to proteolytic products or potentially to proteins produced from aberrant transcripts with perhaps alternative translation start or stop sites. On the other side, a triple hemagglutinin- tagged HA3-TTN5 driven by the 35S promoter did complement the embryo-lethal phenotype of *ttn5-1* (Supplementary Figure S7D, E). α-HA Western blot control performed with plant material from HA3-TTN5 seedlings showed a single band at the correct size, but no band that was 13 to 18 kDa smaller (Supplementary Figure S7D). (...) We did not observe any staining in nuclei or ER when performing HA3-TTN5 immunostaining (Figure 3P; Figure 4A, B), as was the case for fluorescence signals in YFP-TTN5-expressing cells. Presumably, this can indicate that either the nuclear and ER signals seen with YFP-TTN5 correspond to the smaller proteins detected, as described above, or that immunostaining was not suited to detect them. Hence, we focused interpretation on patterns of localization overlapping between the fluorescence staining with YFP-labeled TTN5 and with HA3-TTN5 immunostaining, such as the particular signal patterns in the specific punctate membrane structures."

And we discuss in the Discussion section (starting Line 552):

„We based the TTN5 localization data on tagging approaches with two different detection methods to enhance reliability of specific protein detection. Even though YFP-TTN5 did not complement the embryo-lethality of a *ttn5* loss of function mutant, we made several observations that suggest YFP-TTN5 signals to be meaningful at various membrane sites. We do not know why YFP-TTN5 does not complement. There could be differences in TTN5 levels and interactions in some cell types, which were hindering specifically YFP-TTN5 but not HA3-TTN5. (...) Though constitutively driven, the YFP-TTN5 expression may be delayed or insufficient at the early embryonic stages resulting in the lack of embryo-lethal complementation. On the other hand, the very fast nucleotide exchange activity may be hindered by the presence of a large YFP-tag in comparison with the small HA3-tag which is able to rescue the embryo-lethality. The lack of complementation represents a challenge for the localization of small GTPases with rapid nucleotide exchange in plants. Despite of these limitations, we made relevant observations in our data that made us believe that YFP signals in YFP-TTN5-expressing cells at membrane sites can be meaningful."

© 2024. Published by The Company of Biologists under the terms of the Creative Commons Attribution License (https://creativecommons.org/licenses/by/4.0/).

7. There is some inconsistency within the N.benthamiana images. For example, compare Figure 4C of YFP-TTN5T30N to Figure 4O of YFP-TTN5T30N. Figure 4O is presented as being significant because wortmannin-induced swollen ARA7 compartments are labelled by YFP-TTN5T30N. However, structures very similar to these can already been seen in Figure 4C, which is apparently an unrelated experiment. This, to my mind, is likely a result of the very different expression levels between different cells that can be produced by transient expression in N.benthamiana.

Our response:

Former Figure 4 is now Figure 5. As detailed in our response to comment 2 of reviewer 1:
The reviewer certainly refers to fluorescence images from N. benthamiana *leaf epidermal cells where different circularly shaped structures are visible. In these respective structures, the fluorescent circles are depleted from fluorescence in the center, e.g. in Figure 5C, YFP-fluorescent signals in TTN5^{T30N} transformed leaf discs. We suspect that these structures can be of vacuolar origin as described for similar fluorescent rings in Tichá et al., 2020 for ANNI- GFP (reference in manuscript). The reviewer certainly does not refer to swollen MVBs that are seen following wortmannin treatment, as in Figure 5N-P, which look similar in their shape but are larger in size. Please note that we always included the control conditions, namely the images recorded before the wortmannin treatment, so that we were able to investigate the changes induced by wortmannin. Hence, we can clearly say that the structures with depleted fluorescence in the center as in Figure 5C are not wortmannin-induced swollen MVBs.To make these points clear to the reader, we added an explanation into the text (Line 385-388):*

„We also observed YFP fluorescence signals in the form of circularly shaped ring structures with a fluorescence-depleted center. These structures can be of vacuolar origin as described for similar fluorescent rings in Tichá et al. (2020) for ANNI-GFP."

****Referees cross-commenting****

It sems that all of the reviewers have converged on the conclusion that the in planta characterisation of TTN5 is insufficient to be of substantial interest to the field, highlighting the fact that major improvements are required to strengthen this part of the manuscript and increase its relevance.

Reviewer #2 (Significance (Required)):

General assessment: the strengths of this work are in its in vitro characterisation of TITAN5, however, the in planta characterisation lacks depth.

Significance: the in vitro characterisation of TITAN5 is commendable as such work is lacking for plant GTPases. However, the significance of the work would be boosted substantially by better in planta characterisation, which is where most the most broad interest will lie.

My expertise: my expertise is in in planta characterisation of small GTPases and their interactors.

Our response:
We thank the reviewer for the kind evaluation of our manuscript. We are confident that the changes in the text and NEW Figures and NEW Supplementary Figures will be convincing to consider our work.

Reviewer #3 (Evidence, reproducibility and clarity (Required)):

Summary:

Cellular traffic is an important and well-studied biological process in animal and plant systems. While components involved in transport are known the mechanism by which these components control activity or destination remains to be studied. A critical step in regulating traffic is proper budding and tethering of vesicles. A critical component in determining this step is a family proteins with GTPase activity, which act as switches facilitating vesicle interaction between

© 2024. Published by The Company of Biologists under the terms of the Creative Commons Attribution License (https://creativecommons.org/licenses/by/4.0/).

proteins, or cytoskeleton. The current manuscript by Mohr and colleagues have characterized a small GTPase TITAN5 (TTN5) and identified two residues Gln70 and Thr30 in the protein which they propose to have functional roles. The authors catalogue the localization, GTP hydrolytic activity, and discuss putative functions of TTN5 and the mutants.

Major comments:

The core of the manuscript, which is descriptive characterization of TTN5, lies in reliably demonstrating putative roles. While the GTP hydrolysis rates are well-quantified (though the claims need to be toned down), the microscopy data especially the association of TTN5 with different endomembrane compartments is not convincing due to the quality (low resolution) of the figures submitted. The manuscript text is difficult to navigate due to repetition and inconsistency in the order that the mutants are referred. I am requesting additional experiments which should be feasible considering the authors have all the materials required to perform the experiments and obtain high-quality images which support their claims.
1. In general the figure quality needs to be improved for all microscopy images. I would suggest that the authors highlight 1-2 individual cells to make their point and use the current images as supplementary to establish a broader spread.

Our response:

We have worked substantially on the text and figures to make the content well comprehensive. The mutants are referred to in a consistent manner in the text and figures. We have addressed requested experiments.
As we pointed out in the cover letter and our responses to reviewers 1 and 2, we will upload all raw image data to BioImage Archive upon acceptance of the manuscript so that they can be re-examined without any reduction of resolution. Furthermore, we have conducted new experiments on immunolocalization of HA3-TTN5 (NEW Figure 3P, NEW Figure 4A, B). The text has been improved in several places (see highlighted changes in the manuscript and as detailed in the responses to reviewer 1. We think, this addresses well the reviewers' concerns.

a. Fig. S1 lacks clarity.

Our response:

Supplementary Figure S1 shows TTN5 *gene expression in different organs and growing stages as revealed by transcriptomic data, made available through the AtGenExpress eFB tool of the Bio-Analytic Resource for Plant Biology (BAR). The figure visualizes that* TTN5 *is ubiquitously expressed in different plant organs and tissues, e.g. the epidermis layers that we investigated here, and throughout development including embryo development. In accordance with the embryo-lethal phenotype, this highlights well that* TTN5 *is needed throughout for plant growth and it emphasizes that our investigation of TTN5 localization in epidermis cells is valid.*

We have added a better description to the figure legend. We now also mention the respective publications from which the transcriptome data-sets are derived. The modified figure legend is:

"**Supplementary Figure S1. Visualization of *TTN5* gene expression levels during plant development based on transcriptome data.** Expression levels in (A), different types of aerial organs at different developmental stages; from left to right and bottom to top are represented different seed and plant growth stages, flower development stages, different leaves, vegetative to inflorescence shoot apex, embryo and silique development stages; (B), seedling root tissues based on single cell analysis represented in form of a uniform manifold approximation and projection plot; (C), successive stages of embryo development. As shown in (A) to (C), *TTN5* is ubiquitously expressed in these different plant organs and tissues. In particular, it should be noted that *TTN5* transcripts were detectable in the epidermis cell layer of roots that we used for localization of tagged TTN5 protein in this study. In accordance with the embryo-lethal phenotype, the ubiquitous expression of *TTN5* highlights its importance for plant growth. Original data were derived from (Nakabayashi et al. 2005, Schmid et al. 2005) (A); (Ryu et al. 2019) (B); (Waese et al. 2017) (C). Gene expression levels are indicated by local maximum color code, ranging from the

© 2024. Published by The Company of Biologists under the terms of the Creative Commons Attribution License (https://creativecommons.org/licenses/by/4.0/).

minimum (no expression) in yellow to the maximum (highest expression) in red."

b. For the supplementary videos, it is difficult to determine if punctate structures are moving or is it cytoplasmic streaming? Could this be done with a co-localized marker? Considering that such markers have been used later in Fig. 4?

Our response:
We had detected movement of YFP fluorescent structures in all analyzed YFP-TTN5 plant parts except the root tip. Movement of fluorescence signals in YFP-TTN5^{T30N} seedlings was slowed in hypocotyl epidermis cells. To answer the reviewer comment, we added three NEW supplemental videos (NEW Supplementary Video Material S1M-O) generated with all the three YFP-TTN5 constructs imaged over time in N. benthamiana *leaf epidermal cells upon colocalization with the* cis-*Golgi marker GmMan1-mCherry as requested by the reviewer. In these NEW videos, some of the YFP fluorescent spots seem to move together with the Golgi stacks. GmMan1 is described with a stop-and-go directed movement mediated by the actino- myosin system (Nebenführ 1999) and similarly it might be the case for YFP-TTN5 signals based on the colocalization.*

c. It would be good if the speed of movement is quantified, if the authors want to retain the current claims in results and the discussion.

Our response:
We describe a difference in the movement of YFP fluorescent signal for the YFP-TTN5^{T30N} variant in the hypocotyl compared to YFP-TTN5 and YFP-TTN5^{Q70L}. In hypocotyl cells, we could observe a slowed down or arrested movement specifically of YFP-TTN5^{T30N} fluorescent structures, and we describe this in the Results section (Line 278-291).

"Interestingly, the mobility of these punctate structures differed within the cells when the mutant YFP-TTN5^{T30N} was observed in hypocotyl epidermis cells, but not in the leaf epidermis cells (Supplementary Video Material S1E, compare with S1B) nor was it the case for the YFP- TTN5^{Q70L} mutant (Supplementary Video Material S1F, compare with S1E)."

The slowed movement in the YFP-TTN5^{T30N} mutant is well visible even without quantification. We checked that the manuscript text does not contain overstatements in this regard.

2. Fig.2

a. I am not sure what the unit / scale is in Fig. 2D/E if each parameter (Kon, Koff, and Kd) are individually plotted? Could the authors please clarify/simplify this panel?
Our response:

We presented kinetics for nucleotide association (k_{on}) and dissociation (k_{off}) and the dissociation constant (K_d) in a bar diagram for each nucleotide, mdGDP (Figure 2D) and mGppNHp (Figure 2E). We modified and relabeled the bar diagram representation. It should be now very clear which are the parameters and units. Please see also the other modified figures (NEW modified Figure 2A-H). We also modified the legend of Figure 2D and E:

"(D-E), Kinetics of association and dissociation of fluorescent nucleotides mdGDP (D) or mGppNHp (E) with TTN5 proteins (WT, TTN5^{T30N}, TTN5^{Q70L}) are illustrated as bar charts. The association of mdGDP (0.1 µM) or mGppNHp (0.1 µM) with increasing concentration of TTN5^{WT}, TTN5^{T30N} and TTN5^{Q70L} was measured using a stopped-flow device (see A, B; data see Supplementary Figure S3A-F, S4A-E). Association rate constants (k_{on} in $\mu M^{-1}s^{-1}$) were determined from the plot of increasing observed rate constants (k_{obs} in s^{-1}) against the corresponding concentrations of the TTN5 proteins. Intrinsic dissociation rates (k_{off} in s^{-1}) were determined by rapidly mixing 0.1 µM mdGDP-bound or mGppNHp-bound TTN5 proteins with the excess amount of unlabeled GDP (see A, C, data see Supplementary Figure S3G-I, S4F- H). The nucleotide affinity (dissociation constant or K_d in µM) of the corresponding TTN5 proteins was calculated by dividing k_{off} by k_{on}. When mixing mGppNHp with nucleotide-free TTN5^{T30N}, no binding was observed (n.b.o.) under these

© 2024. Published by The Company of Biologists under the terms of the Creative Commons Attribution License (https://creativecommons.org/licenses/by/4.0/).

experimental conditions."

b. Are panels D and E representing values for mdGDP and GppNHP? This is not very clear from the figure legend.

Our response:

Yes, Figure 2D and E represent the k_{on}, k_{off} and K_d values for mdGDP (Figure 2D) and mGppNHP (Figure 2E). As detailed in our previous response to comment 2a, we modified figure and figure legend to make the representation more clear.

3. Fig. 3

Same comments as in para above - improve resolution fo images, concentrate on a few selected cells, if required use an inset figure to zoom-in to specific compartments.

Our response:

As detailed in our responses to reviewers 1 and 2, we will upload all original image data to BioImage Archive upon acceptance of the manuscript, so that a detailed investigation of all our images is possible without any reduction of resolution.

b. Please provide the non-fluorescent channel images to understand cell topography

Our response:

We presented our microscopic images with the respective fluorescent channel and for colocalization with an additional merge. We did not present brightfield images as the cell topography was already well visible by fluorescent signal close to the PM. Therefore, brightfield images would not provide any benefit. Since we will upload all original data to BioImage Archive for a detailed investigation of all our images, the data can be obtained if needed.

c. Is the nuclear localization seen in transient expression (panel L-N) an artefact? If so, this needs to be mentioned in the text.

Our response:

As explained in our responses to reviewers 1 and 2, fluorescence signals were detected within the nuclei of root cells of YFP-TTN5 plants, while immunostaining signals of HA3-TTN5 were not detected in the nucleus.

In an a-GFP Western blot using YFP-TTN5 Arabidopsis seedlings, we detected besides the expected and strong 48 kDa YFP-TTN5 band, three additional weak bands ranging between 26 to 35 kDa (NEW Supplementary Figure S7C). We cannot explain the presence of these small protein bands. They might correspond to free YFP, to proteolytic products or potentially to proteins expressed from aberrant transcripts. a-HA Western blot controls performed with plant material from HA3-TTN5 seedlings showed a single band at the correct size (Supplementary Figure S7D). We must therefore be cautious about nuclear TTN5 localization and we rephrased the text carefully (starting Line 300):

„We also found multiple YFP bands in α-GFP Western blot analysis using YFP-TTN5 Arabidopsis seedlings. Besides the expected and strong 48 kDa YFP-TTN5 band, we observed three weak bands ranging between 26 to 35 kDa (Supplementary Figure S7C). We cannot explain the presence of these small protein bands. They might correspond to free YFP, to proteolytic products or potentially to proteins produced from aberrant transcripts with perhaps alternative translation start or stop sites. On the other side, a triple hemagglutinin- tagged HA3-TTN5 driven by the 35S promoter did complement the embryo-lethal phenotype of *ttn5-1* (Supplementary Figure S7D, E). α-HA Western blot control performed with plant material from HA3-TTN5 seedlings showed a single band at the correct size, but no band that was 13 to 18 kDa smaller (Supplementary Figure S7D). (...) We did not observe any staining in nuclei or ER when performing HA3-TTN5 immunostaining (Figure 3P; Figure 4A, B), as was the case for fluorescence signals in YFP-TTN5-

© 2024. Published by The Company of Biologists under the terms of the Creative Commons Attribution License (https://creativecommons.org/licenses/by/4.0/).

expressing cells. Presumably, this can indicate that either the nuclear and ER signals seen with YFP-TTN5 correspond to the smaller proteins detected, as described above, or that immunostaining was not suited to detect them. Hence, we focused interpretation on patterns of localization overlapping between the fluorescence staining with YFP-labeled TTN5 and with HA3-TTN5 immunostaining, such as the particular signal patterns in the specific punctate membrane structures."

4. Fig. 4 - In addition to the points made for Fig. 3

a. The authors should consider reducing gain/exposure to improve image clarity. Especially for the punctate structures, which are difficult to observe in TTN5, likely because of the cytoplasmic localization as well.

Our response:

Thank you for this comment. We record image z-stacks and represent in single z-planes. Reducing the gain to decrease the cytoplasmic signal does not increase the clarity of the punctate structures as the signal strength will become weak.. As mentioned above, we will upload all original image data to BioImage Archive for a detailed investigation of all our images without any reduction of resolution.

b. Reducing Agrobacterial load could be considered. OD of 0.4 is a bit much, 0.1 or even 0.05 could be tried. If available try expression in N. tabaccum, which is more amenable to microscopy. However, this is OPTIONAL, benthamiana should suffice.

Our response:

Thank you for the suggestion. We are routinely using N. benthamiana *leaf infiltration. When setting up this method at first, we did not observe different localization results by using different ODs of bacterial cultures. Hence, an OD_{600} of 0.4 is routinely used in our institute. This value is comparable with the literature although some literature reports even higher OD values for infiltration (Norkunas et al., 2018; Drapal et al., 2021; Zhang et al., 2020, Davis et al., 2020; Stephenson et al., 2018).*

c. A standard norm now is to establish the level of colocalization is by quantifying a pearson's or Mander's correlation. Which I believe has been done in the text, I didn't find a plot representing the same? Could the data (which the authors already have) be plotted alongwith "n" as a table or graph?

Our response:

Please check our response to reviewer 1, comment 4.

We like to insist that we performed colocalization very carefully and quantified the data in three different manners. We like to state that there is no general standardized procedure that best suits the idea of a colocalization pattern. Results of colocalization are represented in stem diagrams and table format, including statistical analysis. Colocalization was carried out with the ImageJ plugin JACoP for Pearson's and Overlap coefficients and based on the centroid method. The plotted Pearson's and Overlap coefficients are presented in bar diagrams in Supplementary Figure S8A and C, including statistics. The obtained values by the centroid method are represented in table format in Supplementary Figure S8B and D, which can be considered a standard method (see Ivanov et al., 2014).

Colocalization of two different fluorescence signals was performed for the two channels in a specific chosen region of interest (indicating in % the overlapping signal versus the sum of signal for each channel). The differences between the YFP/mRFP and mRFP/YFP ratios indicate that a higher percentage of ARA7-RFP signal is colocalizing with YFP-TTN5^{Q70L} signal than with the TTN5^{WT} or the TTN5^{T30N} mutant form signals, while the YFP signals have a similar overlap with

© 2024. Published by The Company of Biologists under the terms of the Creative Commons Attribution License (https://creativecommons.org/licenses/by/4.0/).

ARA7-positive structures. This is not a contradiction. Presumably this answers well the questions on colocalization.

Please note that upon acceptance for publication, we will upload all original colocalization data to BioImage Archive. Hence, the high-quality data can be reanalyzed by readers.

d. The cartoons for the action of chemicals are useful, but need a bit more clarity.

Our response:

The schematic explanations of pharmacological treatments and expected outcomes are useful to readers. For a better understanding, we added additional explaining sentences to the figure legends (Figure 5E, M; Figure 6A). We also modified Figure 6A and the corresponding legend.

"(E), Schematic representation of GmMan1 localization at the ER upon brefeldin A (BFA) treatment. BFA blocks ARF-GEF proteins which leads to a loss of Golgi *cis*-cisternae and the formation of BFA-induced compartments due to an accumulation of Golgi stacks up to a redistribution of the Golgi to the ER by fusion of the Golgi with the ER (Renna and Brandizzi 2020)."

"(M), Schematic representation of ARA7 localization in swollen MVBs upon wortmannin treatment. Wortmannin inhibits phosphatidylinositol-3-kinase (PI3K) function leading to the fusion of TGN/EE to swollen MVBs (Renna and Brandizzi 2020)."

"(A), Schematic representation of progressive stages of FM4-64 localization and internalization in a cell. FM4-64 is a lipophilic substance. After infiltration, it first localizes in the plasma membrane, at later stages it localizes to intracellular vesicles and membrane compartments. This localization pattern reflects the endocytosis process (Bolte et al. 2004)."

5. Fig. 5

a. does the Q70L mutant show reduced endocytosis ?

Our response:

We have not investigated this question. As detailed in our response to reviewer 1, we like to emphasize that we agree fully that functional evidences are interesting to assign role for TTN5 in trafficking steps. A phenotype associated with TTN5^{T30N} and TTN5^{Q70L} would be clearly meaningful.

We do report phenotypes for TTN5^{T30N} that are not observed for TTN5 and TTN5^{Q70L}. From the general assumption how GTPases work, it might have been suspected at first that the TTN5^{T30N} and TTN5^{Q70L} mutant forms should result in cell phenotypes or even morphological and physiological phenotypes of plants. However, when we interpreted the GTPase enzyme data, it became clear that TTN5 behaved as a non-classical GTPase with high GTP affinity, including fast GDP dissociation and slow GTP hydrolysis rates. The consequence of this is that unlike a classical GTPase, TTN5 is most likely present in GTP-bound form in the cells in the resting state. The Q70L mutation can lead to a constitutive active GTPase with an impaired hydrolysis activity in certain types of small GTPases. However, TTN5^{Q70L} showed very similar values for nucleotide dissociation and affinity compared to TTN5^{WT} and only a slightly reduced GTP hydrolysis activity. Thus, this atypical biochemical characteristic can explain why TTN5^{Q70L} mutant-expressing plants lack a visible phenotype. The T30N mutation of certain GTPases is described in literature as a dominant-negative version forming a stable GTPase-GEF complex and subsequent prevention of substrate activation. Here, we found that TTN5^{T30N} exhibited a high GDP dissociation rate and low GDP affinity, which can indicate either a dominant-negative mutation or a fast-cycling mutation. Very interestingly, we observed a cellular phenotype of TTN5^{T30N}, namely displaying reduced mobility of YFP fluorescence signals in hypocotyls (Supplementary Video material S1E, compared with high mobility in case of other TTN5 forms in hypocotyls (Supplementary Video material S1D and F). In this revised version, we included root length data, showing that HA3-TTN5^{T30N} seedlings have shorter roots than untransformed wild type, HA3-TTN5 and partly HA3-TTN5^{Q70L} seedlings (NEW Figure 3O). Again, the seedlings of HA3-TTN5^{Q70L} mutants did not have a significantly different root growth phenotype compared to HA3-TTN5 and WT which is fitting with our biochemical data,

© 2024. Published by The Company of Biologists under the terms of the Creative Commons Attribution License (https://creativecommons.org/licenses/by/4.0/).

while, very interestingly, HA3-TTN5^{T30N} seedlings had shorter roots. This root phenotype along with the hypocotyl mobility phenotype may have been caused by the altered GDP kinetics of the HA3- TTN5^{T30N} variant.

Concerning the aspect of colocalization of the mutants with the markers we show in Figure 5C, D and G, H that YFP-TTN5^{T30N}- and YFP-TTN5^{Q70L}-related signals colocalize with the Golgi marker GmMan1-mCherry. Figure 5K, L and O, P show that YFP-TTN5^{T30N} and YFP-TTN5^{Q70L}- related signals can colocalize with the MVB marker, and this may affect relevant vesicle trafficking processes and plasma membrane protein regulation involved in root cell elongation.

At present, we have not yet investigated perturbed cargo trafficking. These aspects are certainly interesting but require extensive work and testing of appropriate physiological conditions and appropriate cargo targets. We discuss future perspectives in the Discussion. We agree that such functional information is of great importance, but needs to be clarified in future studies.

6. The main text needs to be organized in a way that a reader can separate what is the hypothesis/assumption from actual results and conclusions (see lines #143-149).

Our response:

Thank you for this comment. We reformulated text throughout the manuscript.

7. The text is repeated in multiple places, while I understand that this is not plagiarism, the repetitiveness makes it difficult to read and understand the text. I highlight a couple of examples here, but please check the whole text thoroughly and edit/delete as necessary.
a. Lines #124-125 with Lines #149-151
b. Lines #140-143

Our response:

We checked the text and removed unnecessary repetitions.

8. Could the authors elaborate on whether there are plan homologs of TTN5? Also, have other ARF/ARLs been compared to TTN5 beyond HsARF1?

Our response:

Phylogenetic trees of the ARF family in Arabidopsis in comparison to human ARF family were already published by Vernoud et al. (2003). In this phylogenetic tree ARF, ARL and SAR proteins of Arabidopsis are compared with the members in humans and S. cervisiae. *It is difficult to deduce whether the proteins are homologs or orthologs. In this setting, an ortholog of TTN5 may be HsARL2 followed by HsARL3. In Figure 1A we represented some human GTPases as closely related in sequence to TTN5, these are HsARL2, HsARF1 and AtARF1 since they are the best studied ARF GTPases. HRAS is a well-known member of the RAS superfamily which we used for kinetic comparison in Figure 2. We additionally compared published kinetics of RAC1, HsARF3, CDC42, RHOA, ARF6, RAD, GEM, and RAS GTPases.*

9. On a related note, a major problem I have with these kinetic values is the assumption of significance or not. For eg. Line#180 the values represent and 2 and 6-fold increase, if these numbers do not matter can a significance threshold be applied so as to understand how much fold-change is appreciable?

Our response:

The kinetics of TTN5 and its two mutant variants can be compared with those of other studied GTPases. To provide a basis for the statements about differences in GTPase activities, we modified the text and added respective references in the text for comparisons of fold changes.

© 2024. Published by The Company of Biologists under the terms of the Creative Commons Attribution License (https://creativecommons.org/licenses/by/4.0/).

The new text is now as follows Line 175-231):

„ We next measured the dissociation (k_{off}) of mdGDP and mGppNHp from the TTN5 proteins in the presence of excess amounts of GDP and GppNHp, respectively (Figure 2C) and found interesting differences (Figure 2D, E; Supplementary Figures S3G-I, S4F-H). First, TTN5^{WT} showed a k_{off} value (0.012 s^{-1} for mGDP) (Figure 2D; Supplementary Figure S3G), which was 100-fold faster than those obtained for classical small GTPases, including RAC1 (Haeusler et al. 2006) and HRAS (Gremer et al. 2011), but very similar to the k_{off} value of HsARF3 (Fasano et al. 2022). Second, the k_{off} values for mGDP and mGppNHp, respectively, were in a similar range between TTN5^{WT} (0.012 s^{-1} mGDP and 0.001 s^{-1} mGppNHp) and TTN5^{Q70L} (0.025 s^{-1} mGDP and 0.006 s^{-1} mGppNHp), respectively, but the k_{off} values differed 10-fold between the two nucleotides mGDP and mGppNHp in TTN5^{WT} (k_{off} = 0.012 s^{-1} versus k_{off} = 0.001 s^{-1}; Figure 2D, E; Supplementary Figure S3G, I, S4F, H). Thus, mGDP dissociated from proteins 10-fold faster than mGppNHp. Third, the mGDP dissociation from TTN5^{T30N} (k_{off} = 0.149 s^{-1}) was 12.5- fold faster than that of TTN5^{WT} and 37-fold faster than the mGppNHp dissociation of TTN5^{T30N} (k_{off} = 0.004 s^{-1}) (Figure 2D, E; Supplementary Figure S3H, S4G). Mutants of CDC42, RAC1, RHOA, ARF6, RAD, GEM and RAS GTPases, equivalent to TTN5^{T30N}, display decreased nucleotide binding affinity and therefore tend to remain in a nucleotide-free state in a complex with their cognate GEFs (Erickson et al. 1997, Ghosh et al. 1999, Radhakrishna et al. 1999, Jung and Rösner 2002, Kuemmerle and Zhou 2002, Wittmann et al. 2003, Nassar et al. 2010, Huang et al. 2013, Chang and Colecraft 2015, Fisher et al. 2020, Shirazi et al. 2020). Since TTN5^{T30N} exhibits fast guanine nucleotide dissociation, these results suggest that TTN5^{T30N} may also act in either a dominant-negative or fast-cycling manner as reported for other GTPase mutants (Fiegen et al. 2004, Wang et al. 2005, Fidyk et al. 2006, Klein et al. 2006, Soh and Low 2008, Sugawara et al. 2019, Aspenström 2020).

The dissociation constant (K_d) is calculated from the ratio k_{off}/k_{on}, which inversely indicates the affinity of the interaction between proteins and nucleotides (the higher K_d, the lower affinity). Interestingly, TTN5^{WT} binds mGppNHp (K_d = 0.029 µM) 10-fold tighter than mGDP (K_d = 0.267 µM), a difference, which was not observed for TTN5^{Q70L} (K_d for mGppNHp = 0.026 µM, K_d for mGDP = 0.061 µM) (Figure 2D, E). The lower affinity of TTN5^{WT} for mdGDP compared to mGppNHp brings us one step closer to the hypothesis that classifies TTN5 as a non- classical GTPase with a tendency to accumulate in the active (GTP-bound) state (Jaiswal et al. 2013). The K_d value for the mGDP interaction with TTN5^{T30N} was 11.5-fold higher (3.091 µM) than for TTN5^{WT}, suggesting that this mutant exhibited faster nucleotide exchange and lower affinity for nucleotides than TTN5^{WT}. Similar as other GTPases with a T30N exchange, TTN5^{T30N} may behave in a dominant-negative manner in signal transduction (Vanoni et al. 1999).

To get hints on the functionalities of TTN5 during the complete GTPase cycle, it was crucial to determine its ability to hydrolyze GTP. Accordingly, the catalytic rate of the intrinsic GTP hydrolysis reaction, defined as k_{cat}, was determined by incubating 100 µM GTP-bound TTN5 proteins at 25°C and analyzing the samples at various time points using a reversed-phase HPLC column (Figure 2F; Supplementary Figure S5). The determined k_{cat} values were quite remarkable in two respects (Figure 2G). First, all three TTN5 proteins, TTN5^{WT}, TTN5^{T30N} and TTN5^{Q70L}, showed quite similar k_{cat} values (0.0015 s^{-1}, 0.0012 s^{-1}, 0.0007 s^{-1}; Figure 2G; Supplementary Figure S5). The GTP hydrolysis activity of TTN5^{Q70L} was quite high (0.0007 s^{-}corresponding position drastic impair hydrolysis, resulting in a constitutively active GTPase in cells (Hodge et al. 2020, Matsumoto et al. 2021). Second, the k_{cat} value of TTN5^{WT} (0.0015 s^{-1}) although quite low as compared to other GTPases (Jian et al. 2012, Esposito et al. 2019), was 8-fold lower than the determined k_{off} value for mGDP dissociation (0.012 s^{-1}) (Figure 2E). This means that a fast intrinsic GDP/GTP exchange versus a slow GTP hydrolysis can have drastic effects on TTN5 activity in resting cells, since TTN5 can accumulate in its GTP-bound form, unlike the classical GTPase (Jaiswal et al. 2013). To investigate this scenario, we pulled down GST-TTN5 protein from bacterial lysates in the presence of an excess amount of GppNHp in the buffer using glutathione beads and measured the nucleotide-bound form of GST-TTN5 using HPLC. As shown in Figure 2H, isolated GST-TTN5 increasingly bonds GppNHp, indicating that the bound nucleotide is rapidly exchanged for free nucleotide (in this case GppNHp). This is not the case for classical GTPases, which remain in their inactive GDP- bound forms under the same experimental conditions (Walsh et

© 2024. Published by The Company of Biologists under the terms of the Creative Commons Attribution License (https://creativecommons.org/licenses/by/4.0/).

al. 2019, Hodge et al. 2020)."

10 Another issue with the kinetic measurements is the significance levels. Line #198-201. The three proteins are claimed to have similar values and in the nnext line, the Q70L mutant is claimed to be high.

Our response:
Please see our response and changes in the text according in our response to the previous comment 9. We have provided extra explanations and references to clarify why the kinetic behavior of TTN5 is unusual in several respects (Line 215-220).

„First, all three TTN5 proteins, $TTN5^{WT}$, $TTN5^{T30N}$ and $TTN5^{Q70L}$, showed quite similar kcat values (0.0015 s^{-1}, 0.0012 s^{-1}, 0.0007 s^{-1}; Figure 2G; Supplementary Figure S5). The GTP hydrolysis activity of $TTN5^{Q70L}$ was quite high (0.0007 s^{-1}). This was unexpected because, as with most other GTPases, the glutamine mutations at the corresponding position drastic impair hydrolysis, resulting in a constitutively active GTPase in cells (Hodge et al. 2020, Matsumoto et al. 2021)."

11. Provide data for conclusion in line#214-215

Our response:

We agree that a reference should be added after this sentence to make this sentence clearer (Line 228-231).

"As shown in Figure 2H, isolated GST-TTN5 increasingly bonds GppNHp, indicating that the bound nucleotide is rapidly exchanged for free nucleotide (in this case GppNHp). This is not the case for classical GTPases, which remain in their inactive GDP-bound forms under the same experimental conditions (Walsh et al. 2019, Hodge et al. 2020)."

12. How were the mutants studied here identified? random mutation or was it directed based on qualified assumptions?

Our response:

We used the T30N and the Q70L point mutations as such types of mutants had been reported to confer specific phenotypes in these well-conserved amino acid positions in multiple other small GTPases (Erickson et al. 1997, Ghosh et al. 1999, Radhakrishna et al. 1999, Jung and Rösner 2002, Kuemmerle and Zhou 2002, Wittmann et al. 2003, Nassar et al. 2010, Huang et al. 2013, Chang and Colecraft 2015, Fisher et al. 2020, Shirazi et al. 2020). In particular, these positions affect the interaction between small GTPases and their respective guanine nucleotide exchange factor (GEF; T30N) or on GTP hydrolysis (Q70L). We introduced the mutants and described their potential effect on the GTPase cycle in the introduction and cited exemplary literature. Please see also our response to comment 6 and the proposed text changes (Line 142-151).

„In this study, we characterized the nucleotide binding and GTP hydrolysis properties of $TTN5^{WT}$ and two of its mutants, $TTN5^{T30N}$ and $TTN5^{Q70L}$, using heterologously expressed and purified proteins and *in vitro* biochemical assays, as previously established for human GTPases (Eberth and Ahmadian 2009). The experimental workflow is illustrated in Supplementary Figure S2A-E. The dominant-negative $TTN5^{T30N}$ is assumed to preferentially bind GEFs, sequestering them from their proper context, while the constitutively active $TTN5^{Q70L}$ is thought to be defective in hydrolyzing GTP. Equivalent mutants have been frequently used and characterized in previous studies (Scheffzek et al. 1997, Zhou et al. 2006, Newman et al. 2014)."

13. Could more simplification be provided for deifitinition of Kon/Koff values. And can these values be compared between mutants directly?

© 2024. Published by The Company of Biologists under the terms of the Creative Commons Attribution License (https://creativecommons.org/licenses/by/4.0/).

Our response:

We introduce k_{on} and k_{off} in the modified Figure 2D, E, and they are described in the figure legends. Moreover, we present the data for calculations in Supplementary Figures S3, 4, where again we define the values in the respective figure legends.

14. Data provided are not convincing to claim that both the mutant forms have lower association with the Golgi.

Our response:
Our conclusion is that both YFP-TTN5 and YFP-TTN5^{Q70L} fluorescence signals tend to colocalize more with the Golgi-marker signals compared to YFP-TTN5^{T30N} signals as deduced from the centroid-based colocalization method (Line 404-405).

"Hence, the GTPase-active TTN5 forms are likely more present at *cis*-Golgi stacks compared to TTN5^{T30N}."

The Pearson coefficients of all three YFP-TTN5 constructs were nearly identical, but we could identify differences in overlapping centers between the YFP and mCherry channel. 48 % of the GmMan1-mCherry fluorescent cis-*Golgi stacks were overlapping with signal of YFP-TTN5^{Q70L}, while for YFP-TTN5^{T30N} an overlap of only 31 % was detected. This means that less* cis-*Golgi stacks colocalized with signals in the YFP-TTN5^{T30N} mutant than in YFP-TTN5^{Q70L}, which is the statement in our manuscript.*

15. IN general the Authors should strongly consider the claims made in the manuscript. For eg. "This study lays the foundation for studying the functional relationships of this small GTPase" (line 125) is unqualified as this is true for every protein ever studied and published. Considering that TTN was not isolated/identified in this study for the first time this claim doesn't stand.

Our response:

We reformulated the sentence (Line 123-124).

"This study paves the way towards future investigation of the cellular and physiological contexts in which this small GTPase is functional."

a. Line #185 - "characterestics of a dominant-negative...." What is this based on? From the text it is not clear what are the paremeters. Considering that no complementation phenotypes have been presented, this is a far-fetched claim

Our response:
Small GTPases in general are a well studied protein family and the here used mutations T30N and Q70L are conserved amino acids and commonly used for the characterization of the Ras superfamily members. We added explaining sentences with references to the text. The characteristics referred to in the above paragraph is based on the kinetic study.

We modified the text as follows (Line 186-197):

„Third, the mGDP dissociation from TTN5^{T30N} (k_{off} = 0.149 s^{-1}) was 12.5-fold faster than that of TTN5^{WT} and 37-fold faster than the mGppNHp dissociation of TTN5^{T30N} (k_{off} = 0.004 s^{-1}) (Figure 2D, E; Supplementary Figure S3H, S4G). Mutants of CDC42, RAC1, RHOA, ARF6, RAD, GEM and RAS GTPases, equivalent to TTN5^{T30N}, display decreased nucleotide binding affinity and therefore tend to remain in a nucleotide-free state in a complex with their cognate GEFs (Erickson et al. 1997, Ghosh et al. 1999, Radhakrishna et al. 1999, Jung and Rösner 2002, Kuemmerle and Zhou 2002, Wittmann et al. 2003, Nassar et al. 2010, Huang et al. 2013, Chang and Colecraft 2015, Fisher et al. 2020, Shirazi et al. 2020). Since TTN5^{T30N} exhibits fast guanine nucleotide dissociation, these results suggest that TTN5^{T30N} may also act in either a dominant-negative or

© 2024. Published by The Company of Biologists under the terms of the Creative Commons Attribution License (https://creativecommons.org/licenses/by/4.0/).

fast-cycling manner as reported for other GTPase mutants (Fiegen et al. 2004, Wang et al. 2005, Fidyk et al. 2006, Klein et al. 2006, Soh and Low 2008, Sugawara et al. 2019, Aspenström 2020)."

b. The claims in Line #224-227 are exaggerated. Please tone down or delete

Our response:

We rephrased the sentence (Line 240-243).

"Therefore, we propose that TTN5 exhibits the typical functions of a small GTPase based on *in vitro* biochemical activity studies, including guanine nucleotide association and dissociation, but emphasizes its divergence among the ARF GTPases by its kinetics."

c. Line#488-489 - This conclusion is not really supported. At best Authors can claim that TTN5 is associated with trafficking components, but the functional relevance of this association is not determined.

Our response:

We toned down our statement (Line 604-608).

„The colocalization of FM4-64-labeled endocytosed vesicles with fluorescence in YFP-TTN5-expressing cells may indicate that TTN5 is involved in endocytosis and the possible degradation pathway into the vacuole. Our data on colocalization with the different markers support the hypothesis that TTN5 may have functions in vesicle trafficking."

Minor comments:

1. Line #95 - " This rolein vesicle. . . . . " - please clarify which role?

Our response:

We rephrased the sentence (Line 96-99).

„These roles of ARF1 and SAR1 in COPI and II vesicle formation within the endomembrane system are well conserved in eukaryotes which raises the question of whether other plant ARF members are also involved in functioning of the endomembrane system."

2. Line #168 - "we did not observed" please change to "not able to measure/quantify"

Our response:

We changed the text accordingly (Line 169-171).

„A remarkable observation was that we were not able to monitor the kinetics of mGppNHp association with $TTN5^{T30N}$ but observed its dissociation (k_{off} = 0.026 s^{-1}; Figure 2E)."

3. Line#179 - ARF# is human for Arabidopsis?

Our response:

The study of Fasano et al., 2022 is based on human ARF3 and we added the information to the text (Line 180-181)

"(...) very similar to the k_{off} value of HsARF3 (Fasano et al. 2022)."

4. Line #181 - compared to what is the 10-fold difference?

Our response:

The 10-fold difference is between the nucleotides mGDP and mGppNHp, for both $TTN5^{WT}$ and $TTN5^{Q70L}$. We added the information on specific nucleotides to this sentence for a better

© 2024. Published by The Company of Biologists under the terms of the Creative Commons Attribution License (https://creativecommons.org/licenses/by/4.0/).

understanding (Line 181-185).

„Second, the koff values for mGDP and mGppNHp, respectively, were in a similar range between $TTN5^{WT}$ (0.012 s^{-1} mGDP and 0.001 s^{-1} mGppNHp) and $TTN5^{Q70L}$ (0.025 s^{-1} mGDP and 0.006 s^{-1} mGppNHp), respectively, but the koff values differed 10-fold between the two nucleotides mGDP and mGppNHp in $TTN5^{WT}$ (koff = 0.012 s^{-1} versus koff = 0.001 s^{-1}; Figure 2D, E; Supplementary Figure S3G, I, S4F, H).“

5. Lines #314-323 - are diffciult to understand, consider reframing. Same goes for the conclusion following these lines.

Our response:

We added an explanation to these sentences for a better understanding (Line 392-405).

„We performed an additional object-based analysis to compare overlapping YFP fluorescence signals in YFP-TTN5-expressing leaves with GmMan1-mCherry signals (YFP/mCherry ratio) and *vice versa* (mCherry/YFP ratio). We detected 24 % overlapping YFP- fluorescence signals for TTN5 with Golgi stacks, while in YFP-$TTN5^{T30N}$ and YFP-$TTN5^{Q70L}$-expressing leaves, signals only shared 16 and 15 % overlap with GmMan1-mCherry-positive Golgi stacks (Supplementary Figure S8B). Some YFP-signals did not colocalize with the GmMan1 marker. This effect appeared more prominent in leaves expressing YFP-$TTN5^{T30N}$ and less for YFP- $TTN5^{Q70L}$, compared to YFP-TTN5 (Figure 5B-D). Indeed, we identified 48 % GmMan1- mCherry signal overlapping with YFP-positive structures in YFP-$TTN5^{Q70L}$ leaves, whereas 43 and only 31 % were present with YFP fluorescence signals in YFP-TTN5 and YFP-$TTN5^{T30N}$- expressing leaves, respectively (Supplementary Figure S8B), indicating a smaller amount of GmMan1-positive Golgi stacks colocalizing with YFP signals for YFP-$TTN5^{T30N}$. Hence, the GTPase-active TTN5 forms are likely more present at *cis*-Golgi stacks compared to $TTN5^{T30N}$.“

6. Authors might consider a longer BFA treatment (3-4h) to see more clearer ER-Golgi fusion (BFA bodies)

Our response:
We perfomed addtional BFA treatments for HA3-TTN5-expressing Arabidopsis seedlings followed by whole-mount immunostaining and for YFP-TTN5-expressing Arabidopsis lines. In both experiments we could obtain the typical BFA bodies. We included the NEW data in NEW Figure 4B, C

****Referees cross-commenting****
I agree with both my co-reviewers that the manuscript needs substantial improvement in its cell biology based experiments and conclusions thereof. I think the concensus of all reviewers points to weakness in the in-planta experiments which needs to be addressed to understand and characterize TTN5, which is the main goal of the manuscript.

Reviewer #3 (Significance (Required)):

Significance:
The manuscript has general significance in understanding the role of small GTPases which are understudied. Although the manuscript does not advance the field of either intracellular trafficking or organization it holds significance in attempting to characterize proteins involved, which is a prerequisite for further functional studies.

Our response:
Thank you for your detailed analysis of our manuscript and positive assessment. Our study is an advance in the plant vesicle trafficking field.

© 2024. Published by The Company of Biologists under the terms of the Creative Commons Attribution License (https://creativecommons.org/licenses/by/4.0/).

Original submission

First decision letter

MS ID#: JOCES/2024/262315

MS TITLE: Characterization of the small Arabidopsis thaliana GTPase and ADP-ribosylation factor-like 2 protein TITAN5

AUTHORS: Inga Mohr, Amin Mirzaiebadizi, Sibaji Sanyal, Pichaporn Chuenban, Mohammad Reza Ahmadian, Rumen Ivanov, and Petra Bauer

Thank you for sending your manuscript to Journal of Cell Science through Review Commons.

I am happy to tell you that your manuscript has been accepted for publication in Journal of Cell Science, pending standard ethics checks. Where referee reports on this version are available, they are appended below.

© 2024. Published by The Company of Biologists under the terms of the Creative Commons Attribution License (https://creativecommons.org/licenses/by/4.0/).
